# Conserving, Distributing and Managing Genetically Modified Mouse Lines by Sperm Cryopreservation

**DOI:** 10.1371/journal.pone.0002792

**Published:** 2008-07-30

**Authors:** G. Charles Ostermeier, Michael V. Wiles, Jane S. Farley, Robert A. Taft

**Affiliations:** 1 Technology Evaluation and Development, The Jackson Laboratory, Bar Harbor, Maine, United States of America; 2 Reproductive Sciences, The Jackson Laboratory, Bar Harbor, Maine, United States of America; Cairo University, Egypt

## Abstract

**Background:**

Sperm from C57BL/6 mice are difficult to cryopreserve and recover. Yet, the majority of genetically modified (GM) lines are maintained on this genetic background.

**Methodology/Principal Findings:**

Reported here is the development of an easily implemented method that consistently yields fertilization rates of 70±5% with this strain. This six-fold increase is achieved by collecting sperm from the vas deferens and epididymis into a cryoprotective medium of 18% raffinose (w/v), 3% skim milk (w/v) and 477 µM monothioglycerol. The sperm suspension is loaded into 0.25 mL French straws and cooled at 37±1°C/min before being plunged and then stored in LN_2_. Subsequent to storage, the sperm are warmed at 2,232±162°C/min and incubated in *in vitro* fertilization media for an hour prior to the addition of oocyte cumulus masses from superovulated females. Sperm from 735 GM mouse lines on 12 common genetic backgrounds including C57BL/6J, BALB/cJ, 129S1/SvImJ, FVB/NJ and NOD/ShiLtJ were cryopreserved and recovered. C57BL/6J and BALB/cByJ fertilization rates, using frozen sperm, were slightly reduced compared to rates involving fresh sperm; fertilization rates using fresh or frozen sperm were equivalent in all other lines. Developmental capacity of embryos produced using cryopreserved sperm was equivalent, or superior to, cryopreserved IVF-derived embryos.

**Conclusions/Significance:**

Combined, these results demonstrate the broad applicability of our approach as an economical and efficient option for archiving and distributing mice.

## Introduction

Embryo cryopreservation is an effective strategy for managing mouse lines. Its adoption has been limited by the cost, time and the number of animals required. This is especially true for those lines where embryo yields are low, e.g. BALB/c. Cryopreserving sperm is an attractive alternative. However, its widespread use has been limited by the challenge of efficiently recovering cryopreserved sperm from some commonly used inbred strains[1]. In our experience (**Table 1**) and in that of others[2]–[5], the impaired fertility associated with cryopreserved mouse sperm is dependent on genetic background, with sperm from the C57BL/6 backgrounds being particularly sensitive. Yet, this strain is one of the most commonly used for creating and maintaining genetically modified (GM) lines. More than 75% of the 670 mouse lines submitted to The Jackson Laboratory's Repository from January 2004 to January 2006 were maintained on a predominantly C57BL/6J background. Further, The National Institutes of Health are using C57BL/6 embryonic stem (ES) cells to create a resource containing null mutations in every gene in the mouse genome[6]. Thus, it is critical that an effective and efficient method of cryopreserving and recovering C57BL/6 sperm be developed.

**Table 1:** Dependency of impaired fertility on the genetic background of cryopreserved mouse sperm.

Genetic background of sperm donor line	# of GM lines	# females	# IVF oocytes	% of oocytes to 2-cell±StdErr
129S1/SvImJ	13	355	8,760	3±1
C57BL/6J	311	7,173	207,318	6±0
BALB/cByJ	5	71	1,261	8±3
BALB/cJ	12	159	1,937	15±3
B6129SF1/J	67	1,057	29,071	17±2
NOD/ShiLtJ	6	153	2,976	34±11
FVB/NJ	25	681	8,759	25±4
DBA/2J	9	96	2,518	70±7
**Overall**	448	9,743	262,600	23±8

These data (**Table S4**) were generated in The Jackson Laboratory's IVF and Cryopreservation laboratory from 6/4/01 to 1/24/07 using a method modified from Sztein *et al* [*4*] **(Figure S2)**. IVF oocytes were obtained from the # of superovulated inbred females indicated. The standard error (StdErr) was calculated using the # of GM lines for each background.

Since mouse sperm survive cryopreservation with reasonable success[2], the key to an effective sperm cryopreservation and recovery scheme is maximizing post-thaw fertilization capacity. *In vivo*, sperm develop the capacity to fertilize oocytes during transit through the female reproductive tract. As reviewed by Visconti *et al* [7], sperm fertilization ability is associated with plasma membrane reorganization and increases in intracellular calcium levels and in Reactive Oxygen Species[8] (ROS). Because cryopreservation modifies aspects of sperm function associated with fertilization capacity[9], perhaps these processes can be modulated to increase the fertility of cryopreserved mouse sperm. Thus, the objective of this work was to develop economical processes to cryopreserve C57BL/6J sperm that retain or enhance fertilizing capacity.

Because variable cooling and warming rates have been observed with some methods[10], our effort began by defining reproducible processes for cryopreserving and thawing mouse sperm. Our methods were then refined to enhance the ability of cryopreserved C57BL/6J sperm to fertilize C57BL/6J oocytes. The overall effectiveness of our approach as a tool for the routine management of GM mouse lines, was demonstrated by cryopreserving and recovering 735 GM lines maintained on twelve genetic backgrounds, including 527 GM lines maintained on the C57BL/6J background.

## Results and Discussion

### Definition of cooling and warming rates

More C57BL/6J sperm retained intact membranes with moderate cooling (37±1°C/min) than with rapid cooling (94±2°C/min; **Table 2**). This is in accord with previous work[10], [11]. Importantly, using the device and process reported here (**Figure S1**), sperm were consistently cooled at the same rate. The warming rate also affects sperm survival[11], and encouraging results have been reported when thawing at 54°C[12]. We compared thawing cryopreserved C57BL/6J sperm at 37°C and 54°C. No differences in sample warming rate or sperm survival were observed (**Table 2**). However, a greater proportion of oocytes developed into 2-cell embryos when fertilized *in vitro* with sperm thawed at 37°C (**Table 2**). Even though the straws were exposed to the 54°C water bath for only 5 sec, the outer layers of the straw may have warmed to 54°C before the contents in the center had time to equilibrate. Those cells exposed to 54°C and to temperatures above 37°C likely incurred varying degrees of thermal damage, reducing their ability to fertilize. This mechanism is supported by the assertion of Jiang *et al* [13] that thawing sperm at 37°C reduces the risk of thermal damage.

**Table 2:** Suitable cooling and warming rate determination.

Treatment	Cooling rate °C/min (n)	% intact membranes (n)*	
Styrofoam box	37±1^a^ (3)	26±2^a^ (3)	
Stainless steel Dewar	94±2^b^ (3)	15±4^b^ (3)	
**Water bath temp** °**C**	**Warming rate** °**C/min (n)**	**% intact membranes (n)** *	**% of oocytes to 2-cell (n; # oocytes)** **
37	2,232±162^a^ (3)	28±7^a^ (3)	35±4^a^ (3; 723)
54	2,043±212^a^ (3)	19±5^a^ (3)	11±6^b^ (3; 879)

Cooling treatments are detailed in **Figure S1**. Data are means and standard errors from (**n**) replicates. Values with different superscripts are different (p<0.05).

* At least 300 sperm were evaluated for each replicate.

** A replicate is the average percent of CByB6F1/J oocytes developing into 2-cell embryos within four IVF drops. Each replicate represents sperm from a different set of two C57BL/6J males.

### Promoting/maintaining fertilization capacity

**Figure 1:**
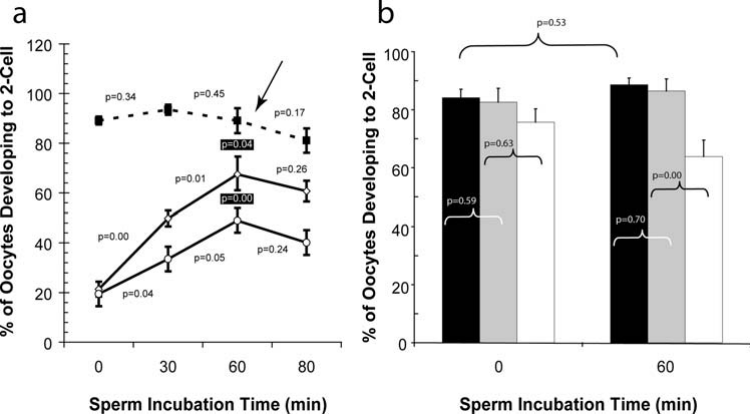
*Influence of sperm pre-incubation on* in vitro *fertilization success.* (a). Variability among males was limited by creating 3 pools of sperm, each containing sperm from 2 C57BL/6J males. Sperm treatments included cryopreservation in 3% skim milk, 18% raffinose (○); 3% skim milk, 18% raffinose supplemented with 477 µM monothioglycerol (◊) and freshly collected in Cook's Mouse Vitro Fert (MVF; ▪). Sperm were cryopreserved and thawed using previously optimized cooling and warming rates. Three IVFs per treatment, each using cumulus oocyte masses from 2 superovulated C57BL/6J females (mean±standard deviation; 66±20 oocytes/IVF), were performed following 0, 30, 60, or 80 min of sperm incubation in MVF. Treatment effects were determined using ANOVA and preplanned contrasts between consecutive time points. The arrow and black boxed p-values indicate the reduced ability of cryopreserved C57BL/6J sperm to fertilize oocytes after 60 min of incubation when compared to freshly collected sperm using Dunnett's Method[32]. (b). Sperm were collected from 6 C57BL/6J males and 3 pools created, each containing sperm from 2 males. The pools were subdivided into 3 treatments: i) *Control* – no treatment (black); ii) *Diluted live sperm* – sperm were diluted 1∶4 in MVF to mimic the concentration of live sperm equivalent to that found in cryopreserved samples (gray); and iii) *Diluted live+dead sperm* – live sperm were diluted 1∶4 with killed sperm (flash frozen in liquid nitrogen) to simulate the number and ratio of live and dead sperm observed in the cryopreserved samples (white). The various treatments received either no incubation or 60 min of incubation in MVF before adding cumulus oocyte masses from two superovulated C57BL/6J oocytes (mean±standard deviation; 69±23 oocytes/IVF). *In vitro* fertilization was done at least six times for each treatment, twice per pool of males. Comparisons indicated were evaluated using preplanned contrast.

Improved IVF success has been reported with cryopreserved C57BL/6 sperm[12], [14]. One characteristic shared by these reports is a processing step that delayed the time between thawing and combining eggs and sperm. To determine if pre-incubating cryopreserved C57BL/6J sperm enhances fertilizing ability, fresh and cryopreserved sperm samples were incubated for 0, 30, 60, or 80 min in Mouse Vitro Fert medium (MVF; Cook Medical; Brisbane, Australia). Freshly collected C57BL/6J sperm did not benefit from incubation. However, cryopreserved sperm exhibited considerable enhancement, with maximum fertility of cryopreserved C57BL/6J sperm being achieved after one hour of incubation (**Figure 1a**). Thus, after thawing, cryopreserved C57BL/6J sperm acquire fertilization competence over time. Similar findings have been reported with sperm incubated in media containing methyl-beta-cyclodextrin[15]. Together, these studies indicate that cryopreserved C57BL/6 sperm must undergo maturational events prior to the addition of oocytes. It is postulated that sperm must commence maturation prior to the addition of oocytes because of the spontaneous cortical granule release, which results in zona hardening and fertilization capacity reduction[16], [17]. In fact, we have observed strain specific differences in the time required for zona pellucida dissolution (personal unpublished data), which may be due to differences in the level of spontaneous cortical granule release or may simply reflect differences in the zona pellucidae from different strains. Nonetheless, if the sperm maturational events are not given time to occur prior to zona hardening, a reduced number of 2-cell embryos or a complete fertilization block would be observed. The need to incubate cryopreserved C57BL/6 sperm is in contrast to previous studies, in which cooling and cryopreservation reduced the time necessary for sperm to obtain fertilization competency[9], [18]. Conceivably, inherent genetic components regulate the differences observed in this phenotype.

Approximately 75% of C57BL/6J sperm do not survive cryopreservation (**Table 2**). Consequently, it may be necessary to increase sperm concentration to ensure that enough viable sperm are present to maximize fertility. However, this could also increase the concentration of dead sperm, which may be detrimental[19]. This hypothesis is supported by reports demonstrating improved fertilization following the separation of viable from non-viable sperm prior to IVF[12], [15]. To determine whether the requirement for a pre-incubation was linked to limited concentrations of viable sperm or increased concentrations of dead sperm, freshly collected sperm were a) diluted to limit the number of live sperm or b) were mixed with dead sperm to simulate the proportion and concentration of dead sperm present following cryopreservation and thawing. Sperm in the various treatments were incubated for 0 or 60 min before C57BL/6J oocytes were added. The proportion of oocytes developing into 2-cell embryos was determined after overnight incubation.

The percentage of oocytes developing into 2-cell embryos did not differ between 0 and 60 min for *Control* (**Figure 1b**), confirming our previous observation that freshly collected C57BL/6J sperm do not require incubation prior to being combined with oocytes. Further, no difference in the proportion of oocytes developing into 2-cell embryos was detected between *Control* and *Diluted live sperm* at time 0 or 60 min (**Figure 1b**). Thus, a broad range of viable sperm concentrations yield similar IVF results and reduced sperm concentrations do not lead to a requirement for sperm pre-incubation. Lastly, we determined if dead sperm or substances released from sperm damaged by cryopreservation interfere with fertilization. The percentage of oocytes developing into 2-cell embryos using *Diluted live sperm* was compared with *Diluted live+dead sperm*. No difference was detected between the two treatments at time 0 (**Figure 1b**), indicating that dead sperm and the substances they release are not responsible for the initial reduction in fertilization capacity of cryopreserved C57BL/6J sperm. In contrast, after 60 min of sperm incubation, the *Diluted live+dead sperm* fertilized ∼26% fewer oocytes than the *Diluted live sperm* (**Figure 1b**). This demonstrates that dead sperm or substances they liberate can be deleterious to the fertilizing capacity of viable sperm following prolonged exposure. Based on these results, it can be concluded that the benefit of a pre-incubation step following thawing is separable from the effects of limiting viable sperm concentrations or increasing the concentration of dead sperm.

**Figure 2:**
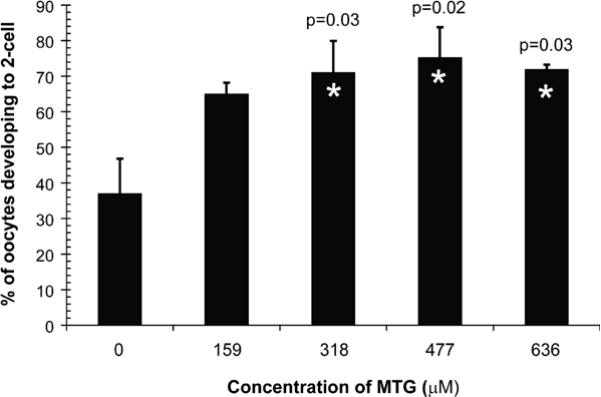
*Dose response of monothioglycerol addition to mouse sperm cryopreservation media.* Sperm were collected from 6 C57BL/6J males. To reduce male-to-male variability, 3 pools of sperm were created by combining 2 males each. The samples were split 5 ways and the sperm were cryopreserved in the concentrations of monothioglycerol (MTG) illustrated on the x-axis. The cryopreserved sperm were thawed, placed into MVF and incubated for 60 min prior to the addition of cumulus oocyte masses from two superovulated C57BL/6J females (mean±standard deviation; 50±22 oocytes/IVF). Differences among the treatments were determined using ANOVA and preplanned comparisons to the control of no MTG using Dunnett's method[32].

While the generation of ROS is necessary for fertilization[20], overproduction of ROS during cryopreservation[21] could alter this process or damage sperm, impairing their ability to fertilize or to support subsequent embryo development. To determine if reducing ROS generation can increase the fertilizing capacity of C57BL/6J sperm, cryopreservation was carried out in the presence of varying concentrations of the reducing agent alpha-monothioglycerol (MTG). This reagent was selected because previous work has shown it is necessary for the culture of ES-cells in sensitive/stressful serum-free systems[22]. The presence of at least 318 µM MTG improved the fertilization proficiency of C57BL/6J sperm (**Figure 2**). This improvement was not associated with an increase in the proportion of motile sperm (p = 0.12), providing further evidence that it is essential to evaluate endpoints other than sperm survival and motility when optimizing cryopreservation methods.

Excess ROS generation may be linked to the requirement for a sperm pre-incubation step, as observed in **Figure 1a**. To test this, C57BL/6J sperm were cryopreserved in the presence of 477 µM MTG and incubated in MVF media for 30, 60, 90 or 120 minutes prior to adding C57BL/6J oocytes. As shown in **Figure 1a**, the presence of MTG did not change the incubation time required to reach maximum fertility. However, the percentage of 2-cell embryos was greater for sperm frozen in the presence of MTG. Collectively, these observations indicate that damage by ROS may be partly responsible for the reduction in fertility of cryopreserved mouse sperm. This effect could be due to the oxidation of lipids[23] and/or proteins[24] that participate in the fusion and subsequent penetration of the oocyte by sperm. Reactive oxygen species can also oxidize DNA[25], potentially leading to a reduction in embryo development. Nonetheless, the observation that cryopreserved C57BL/6J sperm require post-thaw incubation to reach maximum fertility does not appear linked to ROS generation.

### Application of new sperm cryopreservation and recovery methods

The methods reported here yield fertilization rates of 70±5% with C57BL/6J oocytes and cryopreserved C57BL/6J sperm. This is a significant improvement over many previously published approaches [2]–[5], but these data, and those reported by others, may not reflect performance “in the field”. Indeed, the demand for improved sperm cryopreservation methods comes from the necessity to cryopreserve GM lines, not inbred strains. Thus, before reaching conclusions on applicability, we believe it is crucial to test methods as they will be used in practice.

Over a 15-month period (7/19/06 to 10/8/07), sperm were cryopreserved from 994 GM lines (predominantly knockouts and transgenics) submitted to The Jackson Laboratory. For the purpose of this study, the predominant genetic background of the GM line was defined by the inbred strain chosen to be the source of oocytes for IVF. This approach generally holds true, but more importantly, reflects the situation encountered by repositories and core facilities responsible for cryopreservation. To allow for robust statistical comparisons, only those backgrounds represented by five or more GM lines were considered, limiting the data set to 735 GM lines and 12 predominant genetic backgrounds. The entire data set is provided in **Table S1**, which details the Oocyte donor, stock/accession #, strain name, # females, # oocytes, % 2-cell, # embryos transferred and % to liveborn. These values provide detailed information for the genetic background and genetic modification of the publicly available strains investigated (http://www.informatics.jax.org/mgihome/nomen/ and http://jaxmice.jax.org/query/fp205:1:6069276859258683973). The distribution of background strains and genetic modifications likely reflect current utilization frequencies within the scientific community.

**Figure 3:**
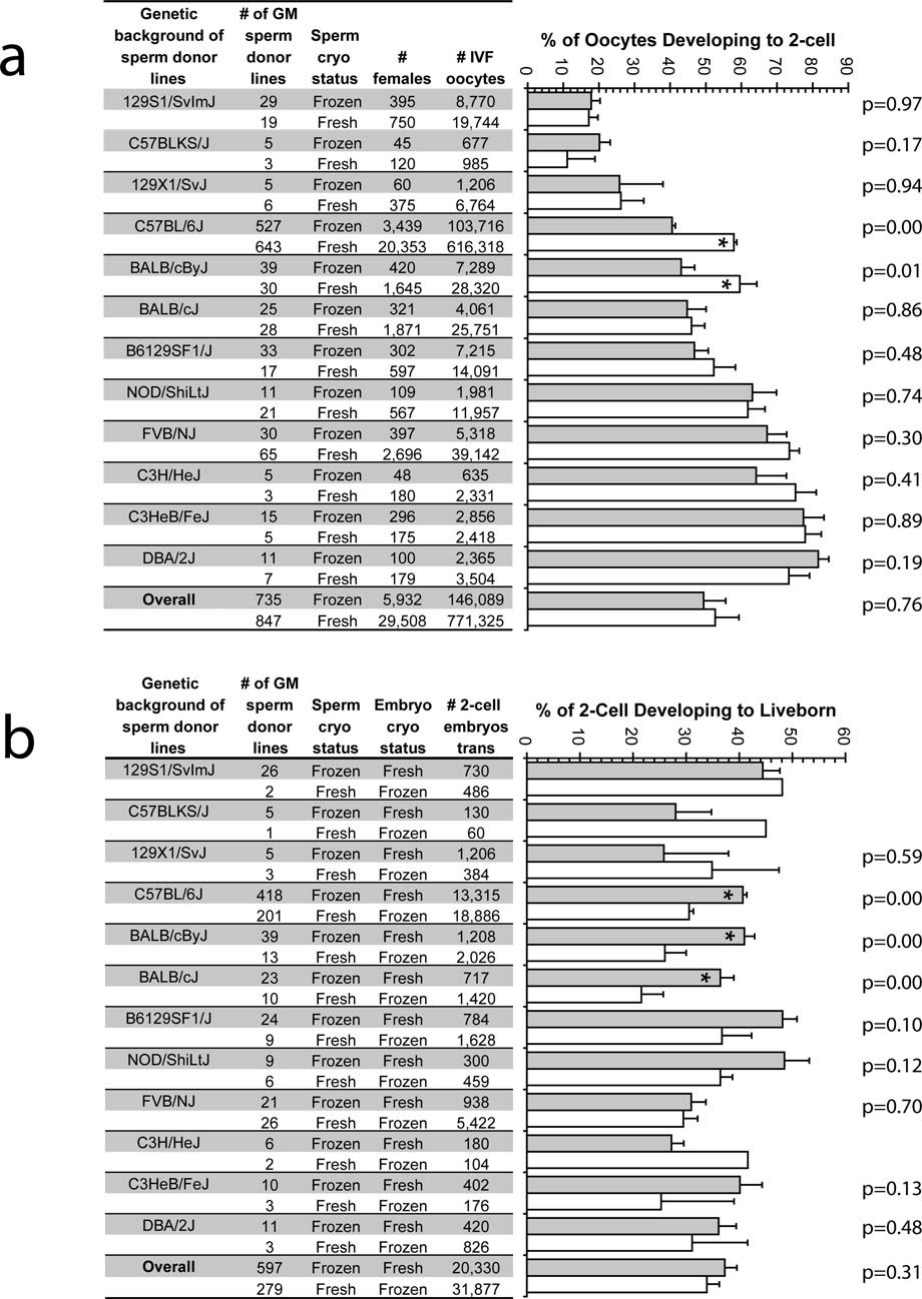
*High throughput application of cryopreserved mouse sperm.* (a). Genetically modified (GM) male mice were grouped based on their predominant background (first column), and the number of unique GM lines within each of the backgrounds is shown (second column). *In vitro* fertilization was preformed employing either our new sperm cryopreservation and recovery methods (Frozen) or freshly collected sperm (Fresh). The number of females and oocytes utilized are shown in the fourth and fifth columns. Differences between the Frozen and Fresh sperm treatments were assessed using ANOVA and preplanned contrasts. Asterisks within a bar indicate a significant difference between the treatments within a background. (b). A subset of the previously created embryos using cryopreserved sperm was transferred directly to pseudopregnant recipients. Those embryos created using freshly collected sperm were cryopreserved prior to transfer, as shown in column four. Differences between the two treatments were assessed using ANOVA and preplanned contrasts. Asterisks within a bar indicate a significant difference between the treatments within a background. No p-value is reported in those instances where the sample size was too small for statistical analysis.

Sperm were cryopreserved as described previously and stored in liquid nitrogen for at least 24 hours. *In vitro* fertilization was performed using 60 minutes of sperm pre-incubation and oocytes from superovulated inbred females corresponding to the predominant genetic background of each of the various GM lines. For comparison, IVF data was obtained using freshly collected sperm from 847 GM lines being maintained on the same genetic backgrounds (**Table S2**). No difference in fertility was detected between freshly collected or cryopreserved sperm for 10 of the 12 genetic backgrounds (129S1/SvImJ; C57BLKS/J; 129X1/SvJ; BALB/cJ; B6129SF1/J; NOD/ShiLtJ; FVB/NJ; C3H/HeJ; C3HeB/FeJ; DBA/2J; **Figure 3a**). Cryopreserved sperm from two of the genetic backgrounds (C57BL/6J and BALB/cByJ) showed slight reductions in fertility. However, when the proportion of 2-cell embryos generated on these two backgrounds using our new method and a method modified from Sztein *et al* [4] were compared, six- and five-fold increases were observed, respectively (**Figure 3a** vs **Table 1**). When frequency distributions are compared, a similar proportion of lines perform poorly (defined as fertilization rate of five percent or less) whether fresh or frozen sperm are used, demonstrating the reliability of the method (**Figure S2**). The difference observed between the fertilization capacity of the GM C57BL/6 lines (**Figure 3a**, **Table 1**) and inbred C57BL/6J mice (**Figure 1a**, **Figure 2**) is likely due to genetic modifications that may have affected fertility and the presence of alleles from other strains in lines that were not fully congenic. Relying on data from inbred strains alone would have overestimated the capability of the method in practice.

The developmental capacity of embryos produced using cryopreserved sperm was compared to that of cryopreserved IVF-derived embryos to illustrate the relative efficiency of different methods available for archiving GM mouse lines. The use of cryopreserved sperm was not associated with a reduction in the percentage of embryos developing to liveborn for any of the groups (**Figure 3b**). However, the use of cryopreserved sperm was associated with an increase in the production of liveborn in three groups (C57BL/6J, BALB/cByJ, BALB/cJ). Perhaps genetic differences among strains predispose IVF produced embryos from C57BL/6J, BALB/cByJ, BALB/cJ mice to cryopreservation damage, resulting in lowered developmental competency. These observations demonstrate that the developmental competence of embryos produced using cryopreserved sperm is equal to or greater than the developmental competence of cryopreserved embryos produced by IVF.

### Implications of efficient mouse sperm cryopreservation and recovery

We report here a simple, inexpensive sperm cryopreservation and recovery method that is easier to implement than other reported methods[12], [14], [15]. The cryopreservation medium is simple to make, the freezing apparatus is easily constructed, sperm separation is not required, and only minor modifications to standard IVF protocols are needed. The breadth of applicability (**Figure 3a**, **Figure 3b**) and efficiency demonstrated (**Figure 3a**, **Figure 3b**, and **Figure S2**) illustrate the general utility and robustness of the approach. Comparable rates of fertilization were observed for most genetic backgrounds whether cryopreserved or freshly collected sperm was used. Likewise, the developmental competence of embryos derived using cryopreserved sperm was comparable or superior to cryopreserved, IVF-derived embryos.

Unless it is necessary to preserve multiple mutations or the entire genome, the efficiencies reported here make sperm cryopreservation preferable to embryo cryopreservation in many cases, provided appropriate females will be available in the future. Sperm cryopreservation can also be preferable to maintaining small colonies. Often, once strains are no longer under active investigation, they are reduced to only a few breeding pairs, placing them at risk from breeding problems, genetic drift, and genetic contamination. When needed, these colonies are scaled up; a process that often takes months. Sperm cryopreservation offers an economical alternative by eliminating ongoing breeding costs and reducing the time to produce experimental cohorts, as the number of oocyte donors for IVF or the number of females used for artificial insemination can easily be scaled up to produce the desired number of animals.

Widespread adoption of sperm cryopreservation will hopefully make the proactive cryopreservation of strains routine. This will benefit facilities and investigators by reducing operating costs, providing protection against disease outbreaks or disasters, and improving collaboration by reducing barriers to distribution. A system in which investigators cryopreserve lines and deposit the cryopreserved material within one of the many publicly funded resource centers may now be possible. This will reduce the operating costs of resource centers, provide secure off-site backup of lines, and relieve investigators and core facilities of the burden of maintaining and distributing lines. The development of an effective, efficient sperm cryopreservation method marks a new paradigm in repository operations, facilitating the global need to archive and distribute mouse models.

## Methods

### Animals

Mice were maintained at The Jackson Laboratory (Bar Harbor, ME, USA) in accordance with The Jackson Laboratory institutional protocols and the *Guide for the Care and Use of Laboratory Animals* [26]. The animals were housed in a minimum barrier facility with a light cycle of 14 hrs on and 10 hrs off (on at 05:00 AM).

### Sperm collection and cryopreservation

The preparation of cryoprotective media and the cooling apparati are detailed in **Figure S1**. Briefly, 2 mL of CryoProtective Medium [CPM - 18% w/v raffinose (Sigma Aldrich; cat # R7630); 3% w/v skim milk (BD Diagnostics; cat # 232100); 477 µM monothioglycerol (Sigma cat # M6145); water (Invitrogen, cat # 15230-238)] were used to collect the sperm from the epididymides and vas deferentia of two 3–5 month-old male mice. Sperm were allowed to disperse from the tissue for 10 min and then 10 µL of sperm+CPM and ballast were loaded into 0.25 mL French straws (IMV; Maple Grove, MN; cat# AAA201). Straws were sealed with an impulse heat sealer (American International Electric; Whittier, CA; model AIE-305HD) and five straws per cassette (Zanders Medical Supplies; Vero Beach, FL; cat #16980/0601) were placed onto a raft (polystyrene 15.5 cm wide×20 cm long×2.5 cm deep) floating in LN_2_ for ≥10 min. This resulted in the sperm being cooled from −10 to −60°C at a rate of 37±1°C/min before being plunged and then stored in LN_2_.

Cooling rates were determined by placing the wire lead of an Ertco TC4000 thermocouple (Dubuque, IA) into the 10 µL volume of sperm+CPM within a straw that was inside a cassette with 5 loaded straws. Data points were obtained every two seconds, and those obtained from a temperature of −10°C to a temperature of −60°C were analyzed by linear regression (JMP 6; SAS Institute, Cary, NC). The calculated slope defined the cooling rate.

Warming rates were determined by moving the straw equipped with the thermocouple directly from the cassette within LN_2_ into a water bath. Data points were obtained every two seconds, and those obtained from a temperature of −175°C to −2°C were analyzed with a linear regression to provide a warming rate.

### In Vitro Fertilization

*In vitro* fertilizations were performed using a modified version of that described previously[27]. *In vitro* fertilization culture medium, Mouse Vitro Fert (Cook Medical; Brisbane, Australia), was used for sperm incubation, IVF and zygote culture. The components of MVF are the same as those listed for modified human tubal fluid[28], except for the substitution of gentamicin for penicillin and streptomycin (personal communication; Cook Medical; Brisbane, Australia). The IVF dishes contained one 500 µL fertilization drop.

Sperm samples were thawed in a 37°C water bath for ∼30 sec. The CPM+sperm was pushed out of the straw into the IVF drop and incubated for 1 hr. The number of sperm within an IVF drop (mean±standard deviation; 6.1±2.5×10^5^) varied depending on the sperm count of the males. Sperm count variation was controlled among treatments by applying treatments to be compared to a single pool of sperm. This resulted in the same sperm concentration being utilized across treatments. Superovulated 17- to 27-day-old female mice were used as oocyte donors. After 4 hrs of co-incubation, the presumptive zygotes were washed, and only those appearing normal were cultured overnight in a 150 µL MVF drop. Approximately 18 hrs after washing, the proportion of oocytes fertilized was calculated by dividing the number of 2-cell embryos by the sum of 2-cell embryos and normally appearing presumptive 1-cell oocytes. Polyspermy and parthenogenesis are negligible in mouse IVF systems[15] (personal unpublished data) and assumed consistent across treatments.

### Sperm Membrane Assessment

Sperm were stained according to the Live/Dead Spermatozoa Viability Kit (Molecular Probes; Eugene, OR). Red (compromised membrane) and green (intact membrane) sperm were visualized and for each analysis three different individuals determined the number of green sperm in a total of at least 100 cells.

### Sperm Motility Assay

Sperm were diluted at least 1∶10 in MVF. The samples were then drawn into capillary tubes (Microslide 1099, VitroCom Inc; Mt Lakes, NJ) or loaded into 80 micron *2X-CEL* chambers (Hamilton Thorne; Beverly, MA). Three fields per sample were manually selected and analyzed using the Hamilton Thorne IVOS computerized semen analyzer (Beverly, MA), with the calibration parameters defined in **Table S3**.

### Embryo Cryopreservation

Two-cell embryos were cryopreserved in 1.5 M propylene glycol as described by Glenister and Rall[29].

### Embryo Transfer

Pseudopregnant CByB6F1/J mice of 9 to 13 weeks of age were used as embryo transfer recipients. Four to 15 embryos were transferred into one oviduct of each female as described by Nagy *et al.* [30].

### Statistical Analysis

Percentages were arcsine transformed[31], and treatment differences were detected by analysis of variance (ANOVA) and preplanned comparisons in JMP 6 (SAS Institute, Cary, NC). For presentation, means and standard errors of percentages are shown.

## Supporting Information

Figure S1 Sperm collection and cryopreservation detail.
(1.38 MB DOC)

Figure S2 Efficiency of genetically modified (GM) line recovery.
(0.05 MB DOC)

Table S1 Data set for sperm cryopreserved and recovered using new method and freshly transferred embryos.
(0.03 MB PDF)

Table S2 Data set for freshly collected sperm and cryopreserved embryos.
(0.06 MB PDF)

Table S3 Calibration parameters used with Hamilton Thorne IVOS computerized semen analyzer (Beverly, MA).
(0.03 MB DOC)

Table S4 Data set for sperm cryopreserved and recovered using a method modified from Sztein et al.
(0.02 MB PDF)
