# Supplementary material for: Conserving, Distributing and Managing Genetically Modified Mouse Lines by Sperm Cryopreservation
Source: PLoS One. 2008 Jul 30;3(7):e2792. doi: 10.1371/journal.pone.0002792 (PMC2453316; doi:10.1371/journal.pone.0002792)
Supplement: Figure S1 — Sperm collection and cryopreservation detail. (1.38 MB DOC) [file pone.0002792.s001.doc]

***
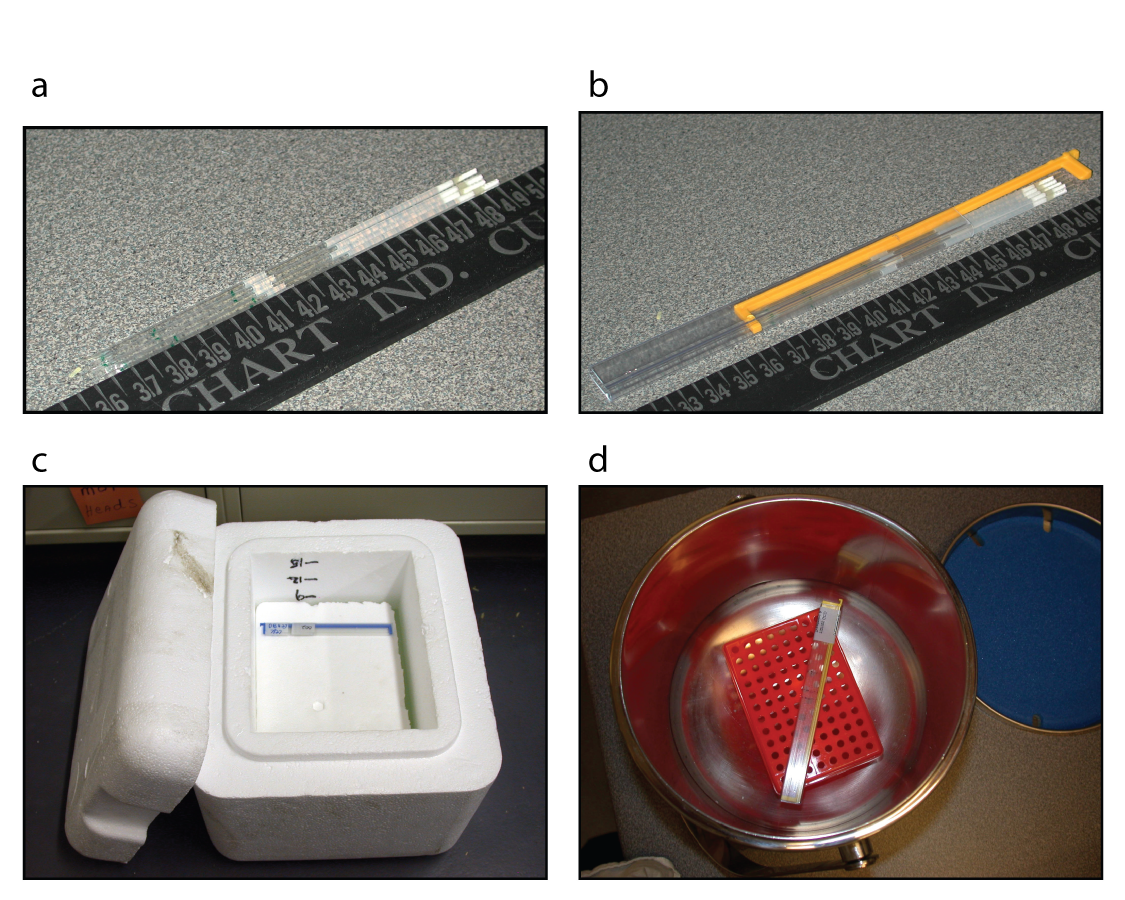
***

***Figure S1. Sperm collection and cryopreservation detail:*** Eighty mL of bottled distilled water (Invitrogen, cat # 15230-238) were placed in a beaker and heated for 40 sec in a microwave (~45°C). The beaker was placed on a heated stir plate (~40°C), 18g of raffinose (Sigma Aldrich; cat # R7630) was added and the solution was heated and stirred until clear. Three g of skim milk (BD Diagnostics; cat # 232100) were added to the raffinose mixture, which was further heated (~40°C) and stirred until the powder dissolved. The solution was then transferred to a volumetric flask and brought to 100 mL with bottled distilled water. Parafilm was placed over the top of the volumetric flask and inverted 3 to 5 times to mix the solution. The 18% raffinose (w/v), 3% skim milk (w/v) solution was centrifuged at 13,000 X g for 15 minutes at room temperature (22°C). The clarified supernatant was filtered through a 0.22 µm cellulose filter (Fisher; Catalog # 431154). The osmolarity was verified and ranged from 470 to 490 mOsm. The cryoprotective media (CPM) was dispensed in 10 mL volumes, into labeled 15 mL conical tubes, capped and stored at -20°C.

The CPM was thawed in a 37°C water bath until all solutes dissolved. The CPM was then supplemented to a final concentration of 477 µM alpha-monothioglycerol (MTG; FW=108.16; D=1.295g/mL; Sigma Aldrich; cat # M6145) and 2 mL were used to collect the sperm from the epididymides and vas deferentia of two 3- to 5-month-old males in a fashion similar to that previously explained[1,2]. (**a**) ***Straw Loading***. The sperm were allowed to disperse from the tissue for 10 min and then in a fashion similar to that proposed by Nakagata[1], loaded into 0.25 mL French straws (IMV; Maple Grove, MN; cat# AAA201) by first creating a 5.5 cm column of CPM, a 2.5 cm column of air, a 0.6 cm column of sperm sample (~10 µL) followed by a column of air. (**b**) ***Cassette Loading.*** The straws were sealed with an instantaneous heat sealer (model AIE-305HD; American International Electric; Whittier, CA) and 5 of them inserted into a cassette (Zanders Medical Supplies; Vero Beach, FL). (**c**) ***Floating raft.*** The cassettes were placed onto a Styrofoam raft (15.5 cm wide x 20 cm long x 2.5 cm deep) situated within a Styrofoam box (outer dimensions – 29 cm wide X 33 cm long x 28.5 cm high: inner dimensions – 21.5 cm long x 17 cm wide x 16.5 cm high) containing 6 to 9 cm of liquid nitrogen. The Styrofoam lid was placed on the box and the samples were exposed to LN_2_ vapor for at least 10 min before being plunged into the liquid phase. (d) ***Dewar setup.*** For comparison, the perforated insert of a pipette tip box was placed into the bottom of a 3L stainless steel Dewar flask (Cole Parmer; Vernon Hills, IL). Liquid nitrogen was added to the middle of the insert and cassettes having 5 straws each were placed on top of the insert and the Dewar lid was replaced. The samples were exposed to LN_2_ vapor for at least 10 min before being plunged into the liquid phase.

**References:**

1. Nakagata N (2000) Cryopreservation of mouse spermatozoa. Mamm Genome 11: 572-576.

2. Sztein JM, Farley JS, Mobraaten LE (2000) In vitro fertilization with cryopreserved inbred mouse sperm. Biol Reprod 63: 1774-1780.
