# Supplementary material for: Conserving, Distributing and Managing Genetically Modified Mouse Lines by Sperm Cryopreservation
Source: PLoS One. 2008 Jul 30;3(7):e2792. doi: 10.1371/journal.pone.0002792 (PMC2453316; doi:10.1371/journal.pone.0002792)
Supplement: Figure S2 — Efficiency of genetically modified (GM) line recovery. (0.05 MB DOC) [file pone.0002792.s002.doc]

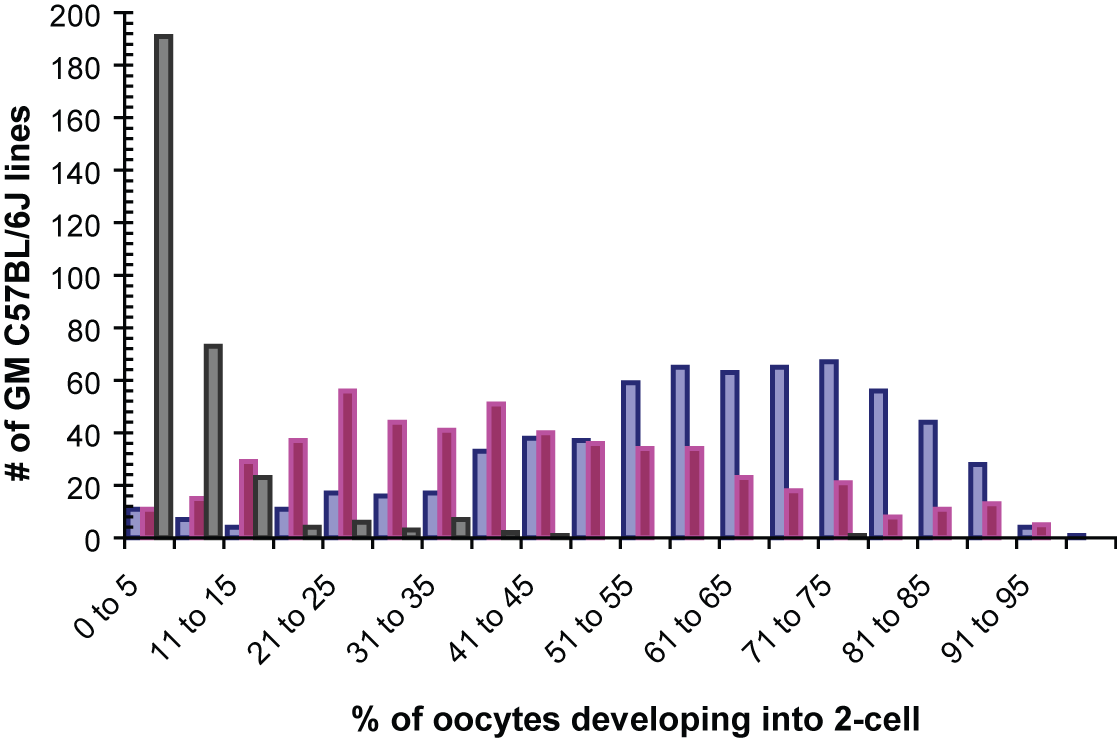


**Figure S2. *Efficiency of genetically modified (GM) line recovery.*** Sperm were collected from GM C57BL/6J mice. The sperm samples were either freshly collected (Blue bars; n=643; **Table S2**), cryopreserved and recovered using our new approach (Pink bars; n=527; **Table S1**), or cryopreserved and recovered using a modified version of that detailed by Sztein *et al*[1] (Black bars; n=311; **Table S4**). Briefly, for the modified Sztein method sperm were collected into a cryoprotective media (CPM) consisting of 18% raffinose and 3% skim milk. Aliquots of sperm+cpm ranging from 50 to 100 µL were placed into 1.8-mL cryotubes (Nunc Cryotubes, Roskilde, Denmark). After capping, the vials were placed in the vapor phase of LN_2_ for 10 min and then plunged into LN_2_ for storage. These samples were thawed in a 37°C water bath for at least 2 min. Ten µL of the sperm+cpm was placed into a 250 µL IVF drop of human tubal fluid[2] and incubated for less than 10 min prior to the addition of cumulus oocyte masses from superovulated inbred females representing the predominant background of the GM male line. The proportion of oocytes developing into 2-cell embryos was determined for all treatments and the GM lines grouped based on their fertilization capacity (x-axis). The number of GM lines in each group is shown on the y-axis.

In theory, if embryos are generated, subsequent line recovery can be achieved. With this definition, 2.9% (9/311), 0.3 % (2/643), and 0.2%(1/527) of the lines cryopreserved and recovered using the modified Sztein method, our new approach, and freshly collected sperm would be non-recoverable, respectively. Nonetheless, if the proportion of oocytes developing into 2-cell embryos is low, a greater amount of resources are necessary for GM line recovery. For example, if 10 pups are required for line recovery, and we assume 40% of embryos develop to term (**Figure 3b**); this would dictate at least 25 embryos are required. If 5% or less of the oocytes within an IVF develop into 2-cell embryos, greater than 500 oocytes are needed. What’s more, if an average of 30 oocytes are obtained from a superovulated C57BL/6J female (**Figure 3a**), then at least 17 females are required for line recovery. This would be the case for 61% (191/311), 2% (11/643) and 2% (11/527) of the GM lines cryopreserved and recovered using the modified Sztein method, our new approach, and freshly collected sperm, respectively. These observations support the view that our new approach to sperm cryopreservation and recovery is a reliable and economical approach for GM line conservation.

**References:**

1. Sztein JM, Farley JS, Mobraaten LE (2000) In vitro fertilization with cryopreserved inbred mouse sperm. Biol Reprod 63: 1774-1780.

2. Nagy A, Gertsenstein M, Vintersten K, Behringer R (2003) Manipulating the mouse embryo: a laboratory manual (3rd ed.): Cold Spring Harbor Laboratory Press. 175, 263-177, 571 p.
