# Supplementary material for: Conserving, Distributing and Managing Genetically Modified Mouse Lines by Sperm Cryopreservation
Source: PLoS One. 2008 Jul 30;3(7):e2792. doi: 10.1371/journal.pone.0002792 (PMC2453316; doi:10.1371/journal.pone.0002792)
Supplement: Table S1 — Data set for sperm cryopreserved and recovered using new method and freshly transferred embryos. (0.03 MB PDF) [file pone.0002792.s003.pdf]

Table S1. ***Data set for sperm cryopreserved and recovered using new method and freshly transferred embryos.***

The first row of the table details the data within each column. The first column depicts the inbred strain that was selected for oocyte donation. The second column represents the stock/accession number for each strain. For public strains, the stock number can be used to obtain detailed strain information by searching the JAX® Mice Database (http://jaxmice.jax.org/query/f?p=205:1:1867898574872980466). Accession numbers were randomly created for private strains, to maintain confidentiality. The third column shows the strain name, which details the genetic background and genetic modification of the strain (http://www.informatics.jax.org/mgihome/nomen/).

The fourth, fifth, and sixth columns indicate the number of females and oocytes used for *in vitro* fertilization and the proportion of oocytes developing into 2-cell embryos for that strain. Columns seven and eight detail the number of embryos transferred and the proportion of these developing into liveborn for the given strain. Data are not provided for all rows in columns seven and eight, as a subset of strains was used for embryo transfer.

Oocyte Donor	Stock/accesion #	Strain Name	# females	# oocytes	% 2-cell	# embryos transferred	% liveborn
129S1/SvImJ	6835	129-Dag1<tm2Kcam>/J	10	246	26.0	45	48.9
129S1/SvImJ	6830	129-Dysf<tm1Kcam>/J	10	224	8.5	18	61.1
129S1/SvImJ	6053	129-Gt(ROSA)26Sor<tm1Luo>/J	10	281	30.2	30	30.0
129S1/SvImJ	6067	129-Gt(ROSA)26Sor<tm2Luo>/J	20	476	14.2	45	51.1
129S1/SvImJ	4446	129-Lta4h<tm1Bhk>/J	13	198	21.2	60	45.0
129S1/SvImJ	6403	129S.B6-Tg(KRT14-Esr1/HRAS)1Pkha/J	12	150	8.0	10	70.0
129S1/SvImJ	5548	129S1/SvImJ-Chr Y<C57BL/6J>/NaJ	11	189	25.9	.	.
129S1/SvImJ	7172	129S6.129X1(B6)-Ptprc<tm1Weis>/J	12	334	3.6	12	58.3
129S1/SvImJ	6820	129S-Catsper3<tm1Clph>/J	38	646	5.3	38	10.5
129S1/SvImJ	6821	129S-Catsper4<tm1Clph>/J	10	197	4.6	9	44.4
129S1/SvImJ	6653	129S-Del(6)1Mom/MomJ	10	335	20.3	30	56.7
129S1/SvImJ	6832	129-Sgcb<tm1Kcam>/J	10	267	3.0	8	50.0
129S1/SvImJ	2866	129-Wnt4<tm1Amc>/J	11	300	29.0	30	63.3
129S1/SvImJ	129S1/SvImJ-1	Private Strain	10	88	43.2	30	30.0
129S1/SvImJ	129S1/SvImJ-10	Private Strain	10	176	11.9	19	63.2
129S1/SvImJ	129S1/SvImJ-11	Private Strain	12	260	1.9	5	20.0
129S1/SvImJ	129S1/SvImJ-12	Private Strain	10	175	32.0	30	16.7
129S1/SvImJ	129S1/SvImJ-13	Private Strain	10	263	4.9	13	69.2
129S1/SvImJ	129S1/SvImJ-14	Private Strain	10	78	32.1	30	46.7
129S1/SvImJ	129S1/SvImJ-15	Private Strain	9	230	14.3	30	63.3
129S1/SvImJ	129S1/SvImJ-16	Private Strain	28	649	0.8	.	.
129S1/SvImJ	129S1/SvImJ-2	Private Strain	9	312	43.3	.	.
129S1/SvImJ	129S1/SvImJ-3	Private Strain	10	383	3.7	16	31.3
129S1/SvImJ	129S1/SvImJ-4	Private Strain	20	540	21.4	46	56.5
129S1/SvImJ	129S1/SvImJ-5	Private Strain	10	212	8.0	16	25.0
129S1/SvImJ	129S1/SvImJ-6	Private Strain	20	379	15.9	54	29.6
129S1/SvImJ	129S1/SvImJ-7	Private Strain	10	188	44.7	30	26.7
129S1/SvImJ	129S1/SvImJ-8	Private Strain	20	493	25.8	46	30.4
129S1/SvImJ	129S1/SvImJ-9	Private Strain	20	501	14.2	30	56.7
129X1/SvJ	129X1/SvJ-1	Private Strain	10	136	8.8	136	8.8
129X1/SvJ	129X1/SvJ-2	Private Strain	10	221	18.1	221	18.1
129X1/SvJ	129X1/SvJ-3	Private Strain	15	337	73.6	337	73.6
129X1/SvJ	129X1/SvJ-4	Private Strain	10	238	8.0	238	8.0
129X1/SvJ	129X1/SvJ-5	Private Strain	15	274	20.4	274	20.4
B6129SF1/J	2727	B6;129-Ahr<tm1Bra>/J	7	79	46.8	30	50.0
B6129SF1/J	3535	B6;129-Gla<tm1Kul>/J	10	84	91.7	30	53.3
B6129SF1/J	6088	B6;129-Mcl1<tm3Sjk>/J	11	259	17.4	30	50.0
B6129SF1/J	2265	B6;129S2-Bcl2<tm1Sjk>/J	8	153	86.3	30	43.3
B6129SF1/J	2217	B6;129S2-Selp<tm1Hyn>/J	6	184	37.0	30	40.0
B6129SF1/J	2458	B6;129S2-Tap1<tm1Arp>/J	5	128	59.4	30	40.0
B6129SF1/J	2273	B6;129S2-Tlx1<tm1Sjk>/J	11	291	33.0	30	56.7
B6129SF1/J	2103	B6;129S2-Trp53<tm1Tyj>/J	8	269	88.5	30	30.0
B6129SF1/J	3366	B6;129S4-Cux1<tm1Ejn>/J	12	319	51.4	30	63.3
B6129SF1/J	2317	B6;129S7-Alpl<tm1Sor>/J	11	272	18.0	30	36.7
B6129SF1/J	3266	B6;129S7-Epas1<tm1Rus>/J	8	175	86.9	.	.
B6129SF1/J	2476	B6;129S7-Ptgs2<tm1Jed>/J	30	667	42.2	97	40.2
B6129SF1/J	2793	B6;129S7-Srd5a1<tm1Mahe>/J	10	194	39.2	30	20.0
B6129SF1/J	2664	B6;129S-Cd4<tm1Mak> Cd8a<tm1Mak>/J	10	310	19.4	30	63.3
B6129SF1/J	2444	B6;129S-Syn1<tm1Sud>/J	10	284	62.7	30	70.0
B6129SF1/J	2478	B6;129S-Syt1<tm1Sud>/J	8	253	39.5	.	.
B6129SF1/J	B6129SF1/J-1	Private Strain	6	308	50.6	30	63.3
B6129SF1/J	B6129SF1/J-10	Private Strain	12	311	53.4	.	.
B6129SF1/J	B6129SF1/J-11	Private Strain	12	242	42.1	.	.
B6129SF1/J	B6129SF1/J-12	Private Strain	10	247	34.0	30	53.3
B6129SF1/J	B6129SF1/J-13	Private Strain	5	104	92.3	.	.
B6129SF1/J	B6129SF1/J-14	Private Strain	6	100	20.0	20	10.0
B6129SF1/J	B6129SF1/J-15	Private Strain	10	153	15.7	14	85.7
B6129SF1/J	B6129SF1/J-2	Private Strain	5	65	63.1	.	.
B6129SF1/J	B6129SF1/J-3	Private Strain	5	65	78.5	.	.
B6129SF1/J	B6129SF1/J-4	Private Strain	5	54	42.6	23	52.2
B6129SF1/J	B6129SF1/J-5	Private Strain	5	55	27.3	15	73.3
B6129SF1/J	B6129SF1/J-6	Private Strain	5	178	51.1	30	43.3
B6129SF1/J	B6129SF1/J-7	Private Strain	7	154	36.4	30	30.0
B6129SF1/J	B6129SF1/J-8	Private Strain	6	179	25.7	30	43.3
B6129SF1/J	B6129SF1/J-9	Private Strain	12	264	2.3	.	.
B6129SF1/J	3534	STOCK Inpp5d<tm1Dmt>/J	18	583	58.7	75	44.0
B6129SF1/J	2403	STOCK Mag<tm1Rod>/J	8	232	31.5	.	.
BALB/cByJ	5463	B6;CByJ-Scn8a<7J>/J	9	220	17.3	30	53.3
BALB/cByJ	5753	B6;CByJ-Spnb4<qv-9J>/J	10	86	68.6	30	30.0
BALB/cByJ	6274	BALB/cByJ-Dfb/J	10	211	9.5	20	45.0
BALB/cByJ	1592	BALB/cByJ-Lpin1<fld>/J	9	132	4.5	5	60.0
BALB/cByJ	5852	BALB/c-Phex<Hyp-Duk>/J	10	117	29.9	30	60.0
BALB/cByJ	6070	C.129S6(B6)-Epha2<tm1Jrui>/J	12	166	39.8	30	56.7
BALB/cByJ	7173	C.129X1(B6)-Ptprc<tm1Weis>/J	12	142	28.9	30	30.0
BALB/cByJ	3581	CBy.129S4-Dab1tm1Cpr/J	10	236	10.2	24	16.7
BALB/cByJ	936	CBy.AK-Tgcog/J	10	213	28.6	30	36.7
BALB/cByJ	5307	CBy.Cg-Thy1<a> Tg(TcraCl4,TcrbCl4)1Shrm/ShrmJ	11	125	52.8	30	26.7
BALB/cByJ	2894	CByJ.A-Atp2b2<dfw-2J>/J	29	588	55.2	135	35.6
BALB/cByJ	1723	CByJ.A-Ttc7<fsn>/J	10	221	79.6	30	46.7

BALB/cByJ	4913	CByJ.B6-Inpp4a<wbl>/FrkJ	9	158	28.5	30	26.7
BALB/cByJ	711	CByJ.Cg-Foxn1<nu>/J	10	244	18.4	30	50.0
BALB/cByJ	3777	CXB5/By-Sil1<wz>/J	10	205	4.9	10	60.0
BALB/cByJ	BALBc/ByJ-1	Private Strain	10	328	13.7	30	20.0
BALB/cByJ	BALBc/ByJ-10	Private Strain	10	85	57.6	30	36.7
BALB/cByJ	BALBc/ByJ-11	Private Strain	10	95	68.4	30	33.3
BALB/cByJ	BALBc/ByJ-12	Private Strain	10	139	33.1	30	30.0
BALB/cByJ	BALBc/ByJ-13	Private Strain	10	186	67.7	30	23.3
BALB/cByJ	BALBc/ByJ-14	Private Strain	10	261	33.7	30	36.7
BALB/cByJ	BALBc/ByJ-15	Private Strain	10	263	43.7	30	43.3
BALB/cByJ	BALBc/ByJ-16	Private Strain	9	175	89.1	30	40.0
BALB/cByJ	BALBc/ByJ-17	Private Strain	10	86	41.9	30	36.7
BALB/cByJ	BALBc/ByJ-18	Private Strain	10	123	26.8	30	30.0
BALB/cByJ	BALBc/ByJ-19	Private Strain	10	160	60.0	30	46.7
BALB/cByJ	BALBc/ByJ-2	Private Strain	10	134	53.0	30	43.3
BALB/cByJ	BALBc/ByJ-20	Private Strain	10	154	56.5	30	50.0
BALB/cByJ	BALBc/ByJ-21	Private Strain	10	197	55.3	30	40.0
BALB/cByJ	BALBc/ByJ-22	Private Strain	10	184	72.8	30	46.7
BALB/cByJ	BALBc/ByJ-23	Private Strain	10	164	34.8	30	50.0
BALB/cByJ	BALBc/ByJ-24	Private Strain	10	213	78.8	30	43.3
BALB/cByJ	BALBc/ByJ-3	Private Strain	10	219	85.8	30	40.0
BALB/cByJ	BALBc/ByJ-4	Private Strain	10	256	8.6	25	20.0
BALB/cByJ	BALBc/ByJ-5	Private Strain	20	328	43.8	40	47.5
BALB/cByJ	BALBc/ByJ-6	Private Strain	10	73	61.6	30	63.3
BALB/cByJ	BALBc/ByJ-7	Private Strain	10	194	46.9	30	36.7
BALB/cByJ	BALBc/ByJ-8	Private Strain	10	105	49.5	30	53.3
BALB/cByJ	BALBc/ByJ-9	Private Strain	10	103	20.4	19	52.6
BALB/cJ	6019	BALB/cJ-ssl/J	10	174	22.4	15	53.3
BALB/cJ	6339	C.129-Btla<tm1Kmm>/J	10	157	74.5	.	.
BALB/cJ	3017	C.129S1-Il12rb1<tm1Jm>/J	20	216	50.5	45	24.4
BALB/cJ	3238	C.129S2(B6)-Ciita<tm1Ccum>/J	10	148	75.7	30	43.3
BALB/cJ	2327	C.129S2-Plat<tm1Mlg>/J	10	157	63.7	30	20.0
BALB/cJ	5440	C.129S4-Ccr3<tm1Cge>/J	50	566	11.2	.	.
BALB/cJ	5316	C.Cg-Fv4<r>/HmJ	10	138	15.2	21	28.6
BALB/cJ	5653	C.Cg-Gata1<tm6Sho>/J	10	70	87.1	30	20.0
BALB/cJ	5420	C;129S7 Gt(ROSA)26Sor-Bmp5<cfe-se7J>/J	20	129	69.8	44	38.1
BALB/cJ	1490	C;AK-Gja8<Lop10>/J	10	145	55.2	45	24.4
BALB/cJ	6560	CBy(Cg)-Gpi1<a-m1J>/BrkJ	10	141	17.7	24	58.3
BALB/cJ	BALB/cJ-1	Private Strain	10	144	79.2	30	46.7
BALB/cJ	BALB/cJ-10	Private Strain	10	135	46.7	30	26.7
BALB/cJ	BALB/cJ-11	Private Strain	10	79	74.7	30	43.3
BALB/cJ	BALB/cJ-12	Private Strain	10	192	87.0	75	34.7
BALB/cJ	BALB/cJ-13	Private Strain	11	183	26.8	45	46.7
BALB/cJ	BALB/cJ-14	Private Strain	10	140	45.7	30	46.7
BALB/cJ	BALB/cJ-2	Private Strain	10	105	60.0	30	23.3
BALB/cJ	BALB/cJ-3	Private Strain	10	131	13.7	18	27.8
BALB/cJ	BALB/cJ-4	Private Strain	10	133	9.0	11	18.2
BALB/cJ	BALB/cJ-5	Private Strain	20	235	20.9	39	30.8
BALB/cJ	BALB/cJ-6	Private Strain	10	113	24.8	27	37.0
BALB/cJ	BALB/cJ-7	Private Strain	10	106	19.8	21	42.9
BALB/cJ	BALB/cJ-8	Private Strain	10	225	51.1	30	50.0
BALB/cJ	BALB/cJ-9	Private Strain	10	99	17.2	17	52.9
C3H/HeJ	5143	C3.B6-Tg(Fabp1-Ccnd1)4Rdb/J	10	138	77.5	30	26.7
C3H/HeJ	784	C3H/HeJ-Fasl<gld>/J	10	156	38.5	30	26.7
C3H/HeJ	513	C3H/HeJ-Oca2<p-J>/J	10	96	53.1	30	23.3
C3H/HeJ	2433	C3H/HeJ-Spnb4<qv-lnd2J>/J	8	131	65.6	30	30.0
C3H/HeJ	C3H/HeJ-1	Private Strain	10	114	86.0	30	36.7
C3Heb/FeJ	6863	C3Fe.B6-Mcm4<chaos3>/J	8	76	97.4	30	43.3
C3Heb/FeJ	3798	C3HeB/FeJ-Scn8a<med>/J	10	108	82.4	30	60.0
C3Heb/FeJ	C3Heb/FeJ-1	Private Strain	8	77	97.4	30	16.7
C3Heb/FeJ	C3Heb/FeJ-10	Private Strain	89	1042	94.1	.	
C3Heb/FeJ	C3Heb/FeJ-11	Private Strain	10	100	76.0	.	
C3Heb/FeJ	C3Heb/FeJ-12	Private Strain	30	170	18.8	80	32.5
C3Heb/FeJ	C3Heb/FeJ-13	Private Strain	60	234	39.8	82	25.6
C3Heb/FeJ	C3Heb/FeJ-2	Private Strain	10	173	89.0	.	
C3Heb/FeJ	C3Heb/FeJ-3	Private Strain	10	99	76.8	30	43.3
C3Heb/FeJ	C3Heb/FeJ-4	Private Strain	10	118	89.0	30	56.7
C3Heb/FeJ	C3Heb/FeJ-5	Private Strain	10	143	88.1	30	46.7
C3Heb/FeJ	C3Heb/FeJ-6	Private Strain	11	87	58.6	.	
C3Heb/FeJ	C3Heb/FeJ-7	Private Strain	10	152	75.0	.	
C3Heb/FeJ	C3Heb/FeJ-8	Private Strain	10	130	84.6	30	40.0
C3Heb/FeJ	C3Heb/FeJ-9	Private Strain	10	147	93.9	30	36.7
C57BL/6J	5543	B6(129)-Duox2<thyd>/J	6	131	9.2	7	57.1
C57BL/6J	4608	B6(Cg)-Htra2<mnd2>/J	6	230	22.2	.	
C57BL/6J	4742	B6(Cg)-Ncf1<m1J>/J	6	218	38.5	30	43.3
C57BL/6J	6111	B6(Cg)-Ush1g<js-2J>/J	13	405	35.2	60	41.7
C57BL/6J	5717	B6(NOD) H2<g7>-Sostdc1<shk>/J	5	183	19.1	30	43.3
C57BL/6J	5789	B6.129-Cer1<tm1Dgen>/J	6	137	36.5	30	26.7
C57BL/6J	7064	B6.129-Crx<tm1Clc>/J	6	207	50.7	30	36.7
C57BL/6J	6836	B6.129-Dag1<tm1Kcam>/J	6	96	20.8	18	38.9
C57BL/6J	6834	B6.129-Dag1<tm2Kcam>/J	6	221	29.4	30	63.3
C57BL/6J	6887	B6.129-Dpagt1<tm2Jxm>/J	5	19	31.6	5	60.0
C57BL/6J	6895	B6.129-Galnt1<tm1Jxm>/J	6	105	43.8	30	43.3
C57BL/6J	6891	B6.129-Mgat1<tm2Jxm>/J	5	84	40.5	30	66.7
C57BL/6J	6892	B6.129-Mgat2<tm1Jxm>/J	5	123	36.6	30	56.7
C57BL/6J	6893	B6.129-Mgat3<tm1Jxm>/J	13	305	29.8	52	0.0
C57BL/6J	2797	B6.129-Notch1<tm1Con>/J	6	170	35.3	30	43.3
C57BL/6J	6431	B6.129P2-Adam21<tm1Dgen>/J	6	211	49.3	30	16.7
C57BL/6J	5774	B6.129P2-Adcy7<tm1Dgen>/J	10	190	29.5	30	16.7
C57BL/6J	2853	B6.129P2-Cbs<tm1Unc>/J	8	178	41.0	30	30.0
C57BL/6J	5801	B6.129P2-Esrra<tm1Dgen>/J	7	356	71.3	30	23.3
C57BL/6J	3025	B6.129P2-Fmr1<tm1Cgr>/J	6	198	27.8	.	
C57BL/6J	5810	B6.129P2-Gabrp<tm1Dgen>/J	10	237	71.3	30	36.7
C57BL/6J	2683	B6.129P2-Hbb-b1<tm1Unc> Hbb-b2<tm1Unc>/J	5	151	46.4	30	33.3
C57BL/6J	2405	B6.129P2-Ncam1<tm1Cgn>/J	25	658	32.0	96	40.6
C57BL/6J	2609	B6.129P2-Nos2<tm1Lau>/J	6	213	41.3	.	

C57BL/6J	5576	B6.129P2-P2rx7<tm1Gab>/J	6	242	32.6	30	50.0
C57BL/6J	3142	B6.129P2-Prlr<tm1Cnp>/J	6	176	61.9	30	26.7
C57BL/6J	5841	B6.129P2-Slc6a9<tm1Dgen>/J	6	189	85.7	30	46.7
C57BL/6J	5843	B6.129P2-Slc9a6<tm1Dgen>/J	21	656	40.6	85	32.9
C57BL/6J	2120	B6.129P2-Tcrd<tm1Mom>/J	7	295	40.0	45	35.6
C57BL/6J	6434	B6.129P2-Tmprss13<tm1Dgen>/J	5	95	69.5	.	
C57BL/6J	5940	B6.129S1-Csf2rb<tm1Cgb>/J	7	253	14.6	28	35.7
C57BL/6J	2693	B6.129S1-Il12b<tm1Jm>/J	5	131	39.7	30	16.7
C57BL/6J	2984	B6.129S1-Il12rb1<tm1Jm>/J	5	196	11.2	22	18.2
C57BL/6J	7016	B6.129S1-Irak3<tm1Flv>/J	6	119	43.7	30	23.3
C57BL/6J	4322	B6.129S1-Mapk10<tm1Flv>/J	14	351	45.2	60	43.3
C57BL/6J	7017	B6.129S1-Ripk2<tm1Flv>/J	6	61	60.7	30	46.7
C57BL/6J	5977	B6.129S2(C)-Stat6<tm1Gru>/J	6	223	74.4	30	43.3
C57BL/6J	2778	B6.129S2-Alox15<tm1Fun>/J	6	215	47.9	30	56.7
C57BL/6J	2401	B6.129S2-Igll1<tm1Cgn>/J	6	195	36.9	30	46.7
C57BL/6J	6141	B6.129S2-Thbs1<tm1Hyn>/J	6	204	30.4	30	56.7
C57BL/6J	6490	B6.129S4-Abcb7<tm1Mdf>/J	6	105	51.4	30	50.0
C57BL/6J	6087	B6.129S4-Cxcl10<tm1Adl>/J	6	257	24.9	30	50.0
C57BL/6J	3174	B6.129S4-Il2rg<tm1Wjl>/J	5	145	29.0	30	33.3
C57BL/6J	6198	B6.129S4-Matk<tm1Sor>/J	10	250	56.8	30	40.0
C57BL/6J	3541	B6.129S4-Ntf3<tm2Jae>/J	12	326	15.7	46	30.4
C57BL/6J	4272	B6.129S4-Pdyn<tm1Ute>/J	8	256	53.5	30	40.0
C57BL/6J	7147	B6.129S4-Soat1<tm1Far>/Pgn	5	114	24.6	28	50.0
C57BL/6J	4125	B6.129S6-Abcb11<tm1Wng>/J	13	413	61.2	60	38.3
C57BL/6J	6236	B6.129S6-Casp6<tm1Flv>/J	6	267	44.6	.	
C57BL/6J	6237	B6.129S6-Casp7<tm1Flv>/J	7	164	43.9	30	43.3
C57BL/6J	6852	B6.129S6-Per2<tm1Jt>/J	5	200	62.0	30	46.7
C57BL/6J	6566	B6.129S6-Ppt1<tm1Hof>/SopJ	6	227	53.3	30	30.0
C57BL/6J	4142	B6.129S7-Aplp2<tm1Dbo>/J	6	221	35.3	30	16.7
C57BL/6J	6833	B6.129-Sgcb<tm1Kcam>/2J	5	134	11.9	15	73.3
C57BL/6J	6301	B6.129S-Id3<tm1Zhu>/J	5	108	27.8	30	46.7
C57BL/6J	7174	B6.129-Sla<tm1Weis>/J	6	235	60.4	30	60.0
C57BL/6J	5623	B6.129S-Shh<tm2(cre/ESR1)Cjt>/J	6	182	78.0	30	26.7
C57BL/6J	6837	B6.129-Sspn<tm1Kcam>/J	6	206	17.5	30	50.0
C57BL/6J	6897	B6.129-St3gal1<tm2Jxm>/J	6	172	27.3	30	36.7
C57BL/6J	6898	B6.129-St3gal2<tm2Jxm>/J	6	196	23.5	30	43.3
C57BL/6J	6899	B6.129-St3gal3<tm1Jxm>/J	6	138	13.8	19	42.1
C57BL/6J	6902	B6.129-St8sia2<tm1Jxm>/J	6	153	21.6	30	43.3
C57BL/6J	5540	B6.129S-Tnf<tm1Gkl>/J	5	180	65.0	30	50.0
C57BL/6J	6388	B6.129-Syt7<tm1Sud>/J	5	180	73.9	30	53.3
C57BL/6J	6199	B6.129X1-Fzd9<tm1Uta>/J	6	210	32.4	.	
C57BL/6J	3126	B6.129X1-Grpr<tm1Jfb>/J	8	288	26.7	30	36.7
C57BL/6J	6072	B6.129X1-Mcl1<tm2Sjk>/J	7	152	21.7	30	46.7
C57BL/6J	7171	B6.129X1-Ptprc<tm1Weis>/J	6	157	25.5	30	30.0
C57BL/6J	4361	B6.129-Xrcc5<tm1Nus>/J	12	393	71.7	60	28.3
C57BL/6J	6073	B6.A-(D4Mit108-D4Mit151)/PgnJ	6	193	10.4	19	42.1
C57BL/6J	6090	B6.A-(D4Mit192-D4Mit108)/PgnJ	6	210	23.3	30	46.7
C57BL/6J	6091	B6.A-(D4Mit192-D4Mit53)/PgnJ	6	227	20.7	30	60.0
C57BL/6J	6092	B6.A-(D4Mit53-D4Mit151)/PgnJ	7	242	44.2	30	50.0
C57BL/6J	6079	B6.A-(D6Mit201)/PgnJ	6	237	19.8	30	40.0
C57BL/6J	6081	B6.A-(D6Mit219a-D6Mit201)/PgnJ	6	151	58.9	30	46.7
C57BL/6J	6082	B6.A-(D6Mit230-D6Mit201)/PgnJ	6	193	57.5	30	56.7
C57BL/6J	6077	B6.A-(D6Mit287-D6Mit201)/PgnJ	6	236	21.2	30	46.7
C57BL/6J	26	B6.C3-Gli3<Xt-J>/J	5	155	60.0	30	23.3
C57BL/6J	6557	B6.C3-Gusb<mps-2J>/BrkJ	6	188	30.3	30	30.0
C57BL/6J	3479	B6.C3-Tg(Fos-luc)1Rnd/J	12	367	43.5	60	41.7
C57BL/6J	6561	B6.CBy(Cg)-Gpi1<a-m1J>/BrkJ	6	195	25.1	30	20.0
C57BL/6J	2306	B6.Cg-an/BrkJ	6	189	31.7	30	53.3
C57BL/6J	6253	B6.Cg-Ap3b1<tm1.1Sms>/J	5	122	33.6	30	26.7
C57BL/6J	6230	B6.Cg-Cebpa<tm1Dgt> Tg(Mx1-cre)1Cgn/J	6	161	6.8	.	
C57BL/6J	3826	B6.Cg-cub/J	6	287	44.6	30	30.0
C57BL/6J	6429	B6.Cg-Dwh/J	5	74	18.9	14	57.1
C57BL/6J	4993	B6.Cg-F2rl1<tm1Mslb>/J	6	186	51.1	30	50.0
C57BL/6J	819	B6.Cg-Foxn1<nu>/J	5	175	28.6	.	
C57BL/6J	6407	B6.Cg-Gusb<mps>/BrkJ	6	289	20.8	30	33.3
C57BL/6J	5912	B6.Cg-Il10<tm1Cgn> (D3Mit49-D3Mit348)/Lt	5	116	51.7	30	36.7
C57BL/6J	5491	B6.Cg-Mapt<tm1(EGFP)Klt> Tg(MAPT)8cPdav/J	6	224	24.1	30	30.0
C57BL/6J	6096	B6.Cg-Msr1<tm1Csk>/J	6	161	21.7	30	53.3
C57BL/6J	566	B6.Cg-Os +/+ Cacna1a<tg-la>/J	6	196	21.4	.	
C57BL/6J	6492	B6.Cg-Pdgfra<tm8Sor>/EiJ	16	364	40.6	60	35.0
C57BL/6J	6194	B6.Cg-Polq<tm1Jcs>/J	5	141	35.5	30	33.3
C57BL/6J	5622	B6.Cg-Shh<tm1(EGFP/cre)Cjt>/J	5	166	57.8	30	46.7
C57BL/6J	5317	B6.Cg-Tg(BAT-lacZ)3Picc/J	6	163	44.8	30	20.0
C57BL/6J	6368	B6.Cg-Tg(Cr2-cre)3Cgn/J	5	175	42.3	.	
C57BL/6J	5000	B6.Cg-Tg(F2RL1)1Mslb/J	7	235	48.1	30	33.3
C57BL/6J	6864	B6.Cg-Tg(Ins1-EGFP)1Hara/J	5	132	62.1	30	60.0
C57BL/6J	2210	B6.Cg-Tg(Mt1)174Bri/J	6	214	34.6	30	36.7
C57BL/6J	6293	B6.Cg-Tg(PDGFB-APPSwInd)20Lms/2J	6	232	23.7	30	36.7
C57BL/6J	4659	B6.Cg-Tg(TIE2GFP)287Sato/1J	12	369	42.0	60	18.3
C57BL/6J	5646	B6.Cg-Zfpm2<tm1Sho>/EiJ	6	313	18.2	30	36.7
C57BL/6J	5068	B6.C-Phex<Hyp-Duk>/J	6	172	11.0	18	22.2
C57BL/6J	3095	B6.D2-ingls/J	6	223	3.6	30	50.0
C57BL/6J	2921	B6.D2N-Ahr<d>/J	6	143	46.9	30	40.0
C57BL/6J	3923	B6.HRS(BKS)-Cpe<fat>/J	6	237	48.9	30	13.3
C57BL/6J	3012	B6.SJL-Slc9a1<swe>/J	18	211	23.0	.	
C57BL/6J	7066	B6.SJL-Tg(Crx-GFP,-ALPP)1Clc/J	6	205	46.3	30	40.0
C57BL/6J	6095	B6.SPRET-(D18Mit192-D18Mit91)/PgnJ	6	247	46.6	30	16.7
C57BL/6J	524	B6.WK-Lama2<dy-2J>/J	6	281	26.3	30	16.7
C57BL/6J	6382	B6;129-Cask<tm1Sud>/J	6	263	50.6	30	56.7
C57BL/6J	6885	B6;129-Man2a1<tm2Jxm>/J	5	31	64.5	19	68.4
C57BL/6J	6894	B6;129-Mgat4a<tm1Jxm>/J	6	92	22.8	21	52.4
C57BL/6J	6378	B6;129-Nrxn2<tm1Sud>/J	6	212	50.9	30	43.3
C57BL/6J	6377	B6;129-Nrxn3<tm1Sud> Nrxn1<tm1Sud> Nrxn2<tm1Sud>/J	6	231	25.1	30	36.7
C57BL/6J	6703	B6;129P2-Gucy2d<tm1Mom>/MomJ	5	133	51.9	30	33.3
C57BL/6J	6704	B6;129P2-Gucy2d<tm2Mom>/MomJ	5	144	86.1	30	40.0
C57BL/6J	6665	B6;129P2-Olfr151<tm13(rI7)Mom>/MomJ	5	153	35.9	30	46.7

C57BL/6J	6689	B6;129P2-Olfr151<tm19Mom>/MomJ	11	493	48.5	60	36.7
C57BL/6J	6648	B6;129P2-Olfr151<tm22Mom>/MomJ	5	141	25.5	30	40.0
C57BL/6J	6666	B6;129P2-Olfr151<tm24(Olfr2)Mom>/MomJ	5	134	50.0	30	50.0
C57BL/6J	6675	B6;129P2-Olfr151<tm25Mom>/MomJ	6	149	91.9	30	60.0
C57BL/6J	6676	B6;129P2-Olfr151<tm26Mom>/MomJ	6	204	25.5	30	63.3
C57BL/6J	6679	B6;129P2-Olfr151<tm27Mom>/MomJ	5	63	54.0	30	43.3
C57BL/6J	6681	B6;129P2-Olfr151<tm29Mom>/MomJ	5	159	64.2	30	30.0
C57BL/6J	6637	B6;129P2-Olfr155<tm1Mom>/MomJ	7	156	7.7	12	66.7
C57BL/6J	6640	B6;129P2-Olfr157<tm1Mom>/MomJ	5	158	66.5	30	36.7
C57BL/6J	6642	B6;129P2-Olfr16<tm1Mom>/MomJ	5	107	93.5	30	56.7
C57BL/6J	6643	B6;129P2-Olfr16<tm2Mom>/MomJ	5	202	92.1	30	43.3
C57BL/6J	6596	B6;129P2-Olfr160<tm4Mom>/MomJ	8	160	15.6	25	60.0
C57BL/6J	6687	B6;129P2-Olfr160<tm7Mom>/MomJ	5	138	73.2	30	23.3
C57BL/6J	6595	B6;129P2-Olfr17<tm1Mom>/MomJ	17	598	30.4	90	25.6
C57BL/6J	6649	B6;129P2-Olfr17<tm5Mom>/MomJ	6	166	86.1	30	26.7
C57BL/6J	6664	B6;129P2-Olfr2<tm1Mom>/MomJ	5	167	24.6	30	36.7
C57BL/6J	6667	B6;129P2-Omp<tm3Mom>/MomJ	12	389	58.6	60	46.7
C57BL/6J	6668	B6;129P2-Omp<tm4(cre)Mom>/MomJ	12	431	81.0	60	56.7
C57BL/6J	6376	B6;129P2-Rims1<tm1Sud>/J	6	159	56.6	30	40.0
C57BL/6J	6374	B6;129P2-Rph3a<tm1Sud>/J	6	116	83.6	30	20.0
C57BL/6J	6705	B6;129P2-Sema3b<tm1Mom>/MomJ	6	94	92.6	30	46.7
C57BL/6J	6710	B6;129P2-Sema3f<tm1Mom>/MomJ	6	127	22.8	30	16.7
C57BL/6J	6385	B6;129P2-Syt1<tm3Sud>/J	5	178	60.1	30	60.0
C57BL/6J	6386	B6;129P2-Syt1<tm5Sud>/J	6	40	92.5	30	46.7
C57BL/6J	6387	B6;129P2-Syt1<tm6Sud>/J	5	156	35.9	30	13.3
C57BL/6J	6380	B6;129P2-Vamp2<tm1Sud>/J	5	229	55.5	30	50.0
C57BL/6J	6384	B6;129-Rims1<tm2Sud>/J	6	226	47.3	30	40.0
C57BL/6J	6594	B6;129S2-Omp<tm1Mom>/MomJ	18	569	20.8	65	27.7
C57BL/6J	6404	B6;129S4-Apoa4<tm1Bres>/J	5	131	48.9	.	
C57BL/6J	7065	B6;129S4-Glra2<tm1Clc>/J	6	207	59.9	30	40.0
C57BL/6J	6414	B6;129S4-Mc4r<tm1Lowl>/J	6	208	57.7	30	53.3
C57BL/6J	6392	B6;129S6-Dnajc5<tm1Sud>/J	5	119	55.5	30	33.3
C57BL/6J	6482	B6;129S6-Lig4<tm1Fwa>/Kvm	5	169	23.1	30	43.3
C57BL/6J	6393	B6;129S6-Lphn1<tm1Sud>/J	6	90	62.2	30	20.0
C57BL/6J	6391	B6;129S6-Pclo<tm1Sud>/J	5	233	50.2	30	53.3
C57BL/6J	6379	B6;129S6-Scamp1<tm1Sud>/J	6	104	56.7	30	46.7
C57BL/6J	6044	B6;129S7-Ephb4<tm1And>/J	6	285	35.1	30	36.7
C57BL/6J	2223	B6;129S7-Uox<tm1Bay>/J	5	165	5.5	9	33.3
C57BL/6J	6381	B6;129S-Stxbp1<tm1Sud>/J	5	163	36.8	30	40.0
C57BL/6J	6680	B6;CBA-Tg(Olfr16*,taulacZ)19Mom/MomJ	5	101	65.3	30	63.3
C57BL/6J	6671	B6;CBA-Tg(Olfr16*,taulacZ)5Mom/MomJ	10	296	82.2	45	24.4
C57BL/6J	6672	B6;CBA-Tg(Olfr16*,taulacZ)7Mom/MomJ	5	224	67.4	30	36.7
C57BL/6J	6673	B6;CBA-Tg(Olfr16,taulacZ)sn2Mom/MomJ	5	199	73.9	30	33.3
C57BL/6J	3299	B6;SWJ-Tg(TIMP3-lacZ)7Jeb/J	18	582	14.2	61	47.5
C57BL/6J	6430	B6Ei.129(Cg)-Figla<tm1Dean>/EiJ	7	235	12.3	28	21.4
C57BL/6J	6812	B6Ei.129(Cg)-Lhx9<tm1Lmgd>/EiJ	11	274	59.4	60	38.3
C57BL/6J	7006	B6Ei.129-Nr0b1<tm1Lja>/EiJ	5	109	52.3	30	56.7
C57BL/6J	6002	B6Ei.129P2(C)-Cbx2<tm1Cim>/EiJ	5	161	39.1	30	23.3
C57BL/6J	7041	B6Ei.129P2-Nr5a1<tm2Klp>/EiJ	5	203	63.5	30	43.3
C57BL/6J	6807	B6Ei.129S6(Cg)-Gata4<tm1.1Sho>/EiJ	6	213	46.5	30	33.3
C57BL/6J	6010	B6Ei.129-Wnt4<tm1Amc>/EiJ	6	258	38.4	30	33.3
C57BL/6J	6305	B6Ei.Cg-Nr0b1<tm1.1Lja> Tg(Sry)2Ei Chr Y<AKR>/EiJ	5	75	38.7	30	33.3
C57BL/6J	2197	C57BL/6-Col1a1<Mov13>/J	6	212	31.1	30	23.3
C57BL/6J	5547	C57BL/6J-Chr Y<129S1/SvImJ>/NaJ	5	228	23.2	.	
C57BL/6J	4399	C57BL/6J-Chr Y<A/J>/NaJ	5	91	84.6	30	33.3
C57BL/6J	7030	C57BL/6JEi-Chr Y<A/J>/EiJ	5	124	48.4	30	50.0
C57BL/6J	7250	C57BL/6JEi-Chr Y<AKR/J>/Ei	6	115	33.9	30	60.0
C57BL/6J	2009	C57BL/6J-Hook1<azh>/J	11	268	12.3	20	55.0
C57BL/6J	5749	C57BL/6J-Myo6<sv-3J>/J	13	341	38.0	60	40.0
C57BL/6J	528	C57BL/6J-Phex<Hyp>/J	6	220	10.9	24	37.5
C57BL/6J	6107	C57BL/6J-rslk<2J>/J	6	218	33.9	30	46.7
C57BL/6J	2226	C57BL/6J-Tg(Alb1HBV)44Bri/J	23	678	34.6	90	50.0
C57BL/6J	6362	C57BL/6J-Tg(CMV-Cox8a/EYFP)17J/J	6	217	53.9	30	63.3
C57BL/6J	3833	C57BL/6J-Tg(Eno2-Ighmpb2)17Cx/Cx	5	206	40.8	30	13.3
C57BL/6J	6567	C57BL/6-Tg(ACTB-EGFP)131Osb/LeySopJ	6	299	38.8	30	36.7
C57BL/6J	5070	C57BL/6-Tg(Csf1r-EGFP-NGFR/FKBP1A/TNFRSF6)2Bck/J	6	219	26.9	30	43.3
C57BL/6J	2628	C57BL/6-Tg(SOD1)10Cje/J	6	15	13.3	.	
C57BL/6J	7264	C57BL/6-Tg(Sry-EGFP)92Ei Tg(Sry)4Ei Chr Y<POS>/EiJ	8	288	17.0	30	53.3
C57BL/6J	6888	C57BL/6-Tg(Zp3-cre)1Gwh/J	8	167	25.7	30	30.0
C57BL/6J	C57BL/6J-1	Private Strain	5	89	34.8	30	60.0
C57BL/6J	C57BL/6J-10	Private Strain	5	138	60.9	.	
C57BL/6J	C57BL/6J-100	Private Strain	6	223	29.1	30	66.7
C57BL/6J	C57BL/6J-101	Private Strain	6	220	25.5	30	30.0
C57BL/6J	C57BL/6J-102	Private Strain	6	220	16.4	30	53.3
C57BL/6J	C57BL/6J-103	Private Strain	6	186	23.7	30	66.7
C57BL/6J	C57BL/6J-104	Private Strain	10	263	14.4	30	23.3
C57BL/6J	C57BL/6J-105	Private Strain	6	191	52.9	30	53.3
C57BL/6J	C57BL/6J-106	Private Strain	10	196	4.6	9	33.3
C57BL/6J	C57BL/6J-107	Private Strain	6	140	57.1	30	16.7
C57BL/6J	C57BL/6J-108	Private Strain	11	403	18.0	60	0.0
C57BL/6J	C57BL/6J-109	Private Strain	6	239	18.8	30	36.7
C57BL/6J	C57BL/6J-11	Private Strain	5	171	25.7	.	
C57BL/6J	C57BL/6J-110	Private Strain	8	304	35.9	30	53.3
C57BL/6J	C57BL/6J-111	Private Strain	7	141	61.7	30	56.7
C57BL/6J	C57BL/6J-112	Private Strain	6	149	54.4	30	63.3
C57BL/6J	C57BL/6J-113	Private Strain	6	158	77.2	30	60.0
C57BL/6J	C57BL/6J-114	Private Strain	6	134	82.1	30	70.0
C57BL/6J	C57BL/6J-115	Private Strain	6	120	33.3	30	10.0
C57BL/6J	C57BL/6J-116	Private Strain	6	219	58.0	30	13.3
C57BL/6J	C57BL/6J-117	Private Strain	14	337	49.0	71	1.4
C57BL/6J	C57BL/6J-118	Private Strain	12	286	19.1	36	47.2
C57BL/6J	C57BL/6J-119	Private Strain	6	184	67.9	30	60.0
C57BL/6J	C57BL/6J-12	Private Strain	5	111	21.6	.	
C57BL/6J	C57BL/6J-120	Private Strain	6	216	59.3	30	80.0
C57BL/6J	C57BL/6J-121	Private Strain	6	75	76.0	30	50.0
C57BL/6J	C57BL/6J-122	Private Strain	6	176	64.8	30	53.3

C57BL/6J	C57BL/6J-123	Private Strain	6	166	86.1	30	36.7
C57BL/6J	C57BL/6J-124	Private Strain	6	226	38.1	30	63.3
C57BL/6J	C57BL/6J-125	Private Strain	6	38	34.2	13	15.4
C57BL/6J	C57BL/6J-126	Private Strain	5	91	62.6	30	33.3
C57BL/6J	C57BL/6J-127	Private Strain	5	116	67.2	30	20.0
C57BL/6J	C57BL/6J-128	Private Strain	5	112	75.0	30	20.0
C57BL/6J	C57BL/6J-129	Private Strain	5	55	40.0	22	27.3
C57BL/6J	C57BL/6J-13	Private Strain	5	114	21.9	.	
C57BL/6J	C57BL/6J-130	Private Strain	5	108	50.0	30	46.7
C57BL/6J	C57BL/6J-131	Private Strain	5	50	86.0	30	33.3
C57BL/6J	C57BL/6J-132	Private Strain	6	139	88.5	30	70.0
C57BL/6J	C57BL/6J-133	Private Strain	6	139	54.0	30	63.3
C57BL/6J	C57BL/6J-134	Private Strain	6	177	72.9	30	46.7
C57BL/6J	C57BL/6J-135	Private Strain	6	116	35.3	30	26.7
C57BL/6J	C57BL/6J-136	Private Strain	6	149	13.4	20	85.0
C57BL/6J	C57BL/6J-137	Private Strain	6	212	3.3	7	57.1
C57BL/6J	C57BL/6J-138	Private Strain	6	164	22.0	30	6.7
C57BL/6J	C57BL/6J-139	Private Strain	6	169	56.2	30	46.7
C57BL/6J	C57BL/6J-14	Private Strain	5	152	26.3	.	
C57BL/6J	C57BL/6J-140	Private Strain	6	156	65.4	30	10.0
C57BL/6J	C57BL/6J-141	Private Strain	6	168	67.9	30	30.0
C57BL/6J	C57BL/6J-142	Private Strain	6	158	28.5	30	50.0
C57BL/6J	C57BL/6J-143	Private Strain	6	129	43.4	30	46.7
C57BL/6J	C57BL/6J-144	Private Strain	6	172	89.0	30	30.0
C57BL/6J	C57BL/6J-145	Private Strain	6	71	80.3	30	50.0
C57BL/6J	C57BL/6J-146	Private Strain	6	203	64.0	30	60.0
C57BL/6J	C57BL/6J-147	Private Strain	6	296	12.2	30	36.7
C57BL/6J	C57BL/6J-148	Private Strain	6	153	38.6	30	50.0
C57BL/6J	C57BL/6J-149	Private Strain	5	196	47.4	.	
C57BL/6J	C57BL/6J-15	Private Strain	5	179	13.4	.	
C57BL/6J	C57BL/6J-150	Private Strain	5	160	32.5	.	
C57BL/6J	C57BL/6J-151	Private Strain	5	151	21.9	.	
C57BL/6J	C57BL/6J-152	Private Strain	5	147	33.3	.	
C57BL/6J	C57BL/6J-153	Private Strain	5	160	27.5	.	
C57BL/6J	C57BL/6J-154	Private Strain	5	167	25.7	.	
C57BL/6J	C57BL/6J-155	Private Strain	5	187	16.0	.	
C57BL/6J	C57BL/6J-156	Private Strain	5	151	23.2	.	
C57BL/6J	C57BL/6J-157	Private Strain	5	131	13.7	.	
C57BL/6J	C57BL/6J-158	Private Strain	5	127	15.7	.	
C57BL/6J	C57BL/6J-159	Private Strain	6	160	19.4	.	
C57BL/6J	C57BL/6J-16	Private Strain	5	126	6.3	.	
C57BL/6J	C57BL/6J-160	Private Strain	5	163	22.1	.	
C57BL/6J	C57BL/6J-161	Private Strain	11	511	1.0	.	
C57BL/6J	C57BL/6J-162	Private Strain	5	246	23.2	.	
C57BL/6J	C57BL/6J-163	Private Strain	6	195	52.8	30	56.7
C57BL/6J	C57BL/6J-164	Private Strain	5	105	66.7	30	50.0
C57BL/6J	C57BL/6J-165	Private Strain	5	129	79.8	30	33.3
C57BL/6J	C57BL/6J-166	Private Strain	5	146	30.8	30	36.7
C57BL/6J	C57BL/6J-167	Private Strain	5	241	39.4	30	43.3
C57BL/6J	C57BL/6J-168	Private Strain	5	155	61.3	.	
C57BL/6J	C57BL/6J-169	Private Strain	6	131	37.4	30	6.7
C57BL/6J	C57BL/6J-17	Private Strain	5	234	23.5	.	
C57BL/6J	C57BL/6J-170	Private Strain	8	156	23.1	30	20.0
C57BL/6J	C57BL/6J-171	Private Strain	6	199	45.2	.	
C57BL/6J	C57BL/6J-172	Private Strain	5	234	40.6	.	
C57BL/6J	C57BL/6J-173	Private Strain	5	229	34.5	.	
C57BL/6J	C57BL/6J-174	Private Strain	5	229	47.6	.	
C57BL/6J	C57BL/6J-175	Private Strain	5	229	17.5	.	
C57BL/6J	C57BL/6J-176	Private Strain	6	247	15.0	.	
C57BL/6J	C57BL/6J-177	Private Strain	6	296	31.8	.	
C57BL/6J	C57BL/6J-178	Private Strain	6	300	12.7	.	
C57BL/6J	C57BL/6J-179	Private Strain	6	234	38.0	.	
C57BL/6J	C57BL/6J-18	Private Strain	5	215	50.7	.	
C57BL/6J	C57BL/6J-180	Private Strain	6	231	39.0	.	
C57BL/6J	C57BL/6J-181	Private Strain	5	128	34.4	.	
C57BL/6J	C57BL/6J-182	Private Strain	9	211	3.8	.	
C57BL/6J	C57BL/6J-183	Private Strain	10	147	55.1	.	
C57BL/6J	C57BL/6J-184	Private Strain	6	267	21.0	.	
C57BL/6J	C57BL/6J-185	Private Strain	6	199	72.9	.	
C57BL/6J	C57BL/6J-186	Private Strain	6	184	56.5	30	66.7
C57BL/6J	C57BL/6J-187	Private Strain	6	162	11.7	.	
C57BL/6J	C57BL/6J-188	Private Strain	6	146	16.4	23	43.5
C57BL/6J	C57BL/6J-189	Private Strain	6	211	15.2	30	63.3
C57BL/6J	C57BL/6J-19	Private Strain	5	181	26.0	.	
C57BL/6J	C57BL/6J-190	Private Strain	6	201	40.3	30	33.3
C57BL/6J	C57BL/6J-191	Private Strain	6	154	48.1	30	33.3
C57BL/6J	C57BL/6J-192	Private Strain	6	225	8.4	17	29.4
C57BL/6J	C57BL/6J-193	Private Strain	6	218	30.7	41	41.5
C57BL/6J	C57BL/6J-194	Private Strain	6	252	29.4	30	43.3
C57BL/6J	C57BL/6J-195	Private Strain	6	211	61.1	30	46.7
C57BL/6J	C57BL/6J-196	Private Strain	6	276	44.2	30	23.3
C57BL/6J	C57BL/6J-197	Private Strain	6	223	28.7	30	40.0
C57BL/6J	C57BL/6J-198	Private Strain	6	218	41.3	30	63.3
C57BL/6J	C57BL/6J-199	Private Strain	6	235	57.9	30	23.3
C57BL/6J	C57BL/6J-2	Private Strain	6	238	16.8	30	50.0
C57BL/6J	C57BL/6J-20	Private Strain	5	167	56.9	.	
C57BL/6J	C57BL/6J-200	Private Strain	6	272	44.9	30	16.7
C57BL/6J	C57BL/6J-201	Private Strain	6	216	38.9	30	16.7
C57BL/6J	C57BL/6J-202	Private Strain	6	177	28.8	30	36.7
C57BL/6J	C57BL/6J-203	Private Strain	6	195	25.1	30	40.0
C57BL/6J	C57BL/6J-204	Private Strain	6	193	36.3	30	53.3
C57BL/6J	C57BL/6J-205	Private Strain	6	177	21.5	30	50.0
C57BL/6J	C57BL/6J-206	Private Strain	6	273	42.1	.	
C57BL/6J	C57BL/6J-207	Private Strain	6	238	16.0	.	
C57BL/6J	C57BL/6J-208	Private Strain	6	293	48.1	.	
C57BL/6J	C57BL/6J-209	Private Strain	5	191	44.0	30	26.7

C57BL/6J	C57BL/6J-21	Private Strain	5	252	22.2	.	
C57BL/6J	C57BL/6J-210	Private Strain	5	188	43.6	30	60.0
C57BL/6J	C57BL/6J-211	Private Strain	5	202	40.6	30	53.3
C57BL/6J	C57BL/6J-212	Private Strain	6	246	54.1	30	33.3
C57BL/6J	C57BL/6J-213	Private Strain	6	246	39.0	30	43.3
C57BL/6J	C57BL/6J-214	Private Strain	6	212	73.1	30	43.3
C57BL/6J	C57BL/6J-215	Private Strain	6	262	12.2	30	53.3
C57BL/6J	C57BL/6J-216	Private Strain	14	359	20.8	40	47.5
C57BL/6J	C57BL/6J-217	Private Strain	6	206	42.7	30	23.3
C57BL/6J	C57BL/6J-218	Private Strain	11	204	48.5	30	53.3
C57BL/6J	C57BL/6J-219	Private Strain	5	136	9.6	13	46.2
C57BL/6J	C57BL/6J-22	Private Strain	10	81	14.4	.	
C57BL/6J	C57BL/6J-220	Private Strain	6	194	5.2	9	77.8
C57BL/6J	C57BL/6J-221	Private Strain	5	196	38.8	30	13.3
C57BL/6J	C57BL/6J-222	Private Strain	6	174	9.2	.	
C57BL/6J	C57BL/6J-223	Private Strain	9	391	22.3	45	33.3
C57BL/6J	C57BL/6J-224	Private Strain	4	141	39.7	.	
C57BL/6J	C57BL/6J-225	Private Strain	4	79	51.9	.	
C57BL/6J	C57BL/6J-226	Private Strain	14	289	2.2	.	
C57BL/6J	C57BL/6J-227	Private Strain	8	168	24.2	.	
C57BL/6J	C57BL/6J-228	Private Strain	4	22	36.4	.	
C57BL/6J	C57BL/6J-229	Private Strain	4	92	41.3	.	
C57BL/6J	C57BL/6J-23	Private Strain	6	189	29.6	30	50.0
C57BL/6J	C57BL/6J-230	Private Strain	4	110	38.2	.	
C57BL/6J	C57BL/6J-231	Private Strain	4	71	60.6	.	
C57BL/6J	C57BL/6J-232	Private Strain	4	107	35.5	.	
C57BL/6J	C57BL/6J-233	Private Strain	4	116	12.1	.	
C57BL/6J	C57BL/6J-234	Private Strain	4	96	65.6	.	
C57BL/6J	C57BL/6J-235	Private Strain	4	102	34.3	.	
C57BL/6J	C57BL/6J-236	Private Strain	4	159	16.4	.	
C57BL/6J	C57BL/6J-237	Private Strain	4	86	25.6	.	
C57BL/6J	C57BL/6J-238	Private Strain	4	71	21.1	.	
C57BL/6J	C57BL/6J-239	Private Strain	6	163	17.8	29	41.4
C57BL/6J	C57BL/6J-24	Private Strain	6	171	37.4	30	26.7
C57BL/6J	C57BL/6J-240	Private Strain	5	97	15.5	15	80.0
C57BL/6J	C57BL/6J-241	Private Strain	6	198	26.8	30	53.3
C57BL/6J	C57BL/6J-242	Private Strain	6	217	18.9	30	50.0
C57BL/6J	C57BL/6J-243	Private Strain	15	511	11.6	49	0.0
C57BL/6J	C57BL/6J-244	Private Strain	9	293	25.6	45	48.9
C57BL/6J	C57BL/6J-245	Private Strain	6	117	41.9	30	36.7
C57BL/6J	C57BL/6J-246	Private Strain	6	193	37.8	30	30.0
C57BL/6J	C57BL/6J-247	Private Strain	6	237	33.3	30	63.3
C57BL/6J	C57BL/6J-248	Private Strain	6	141	70.9	30	53.3
C57BL/6J	C57BL/6J-249	Private Strain	6	247	43.3	30	36.7
C57BL/6J	C57BL/6J-25	Private Strain	6	175	68.0	30	43.3
C57BL/6J	C57BL/6J-250	Private Strain	6	168	76.2	30	16.7
C57BL/6J	C57BL/6J-251	Private Strain	6	196	4.6	8	50.0
C57BL/6J	C57BL/6J-252	Private Strain	10	369	0.9	.	
C57BL/6J	C57BL/6J-253	Private Strain	5	121	29.8	.	
C57BL/6J	C57BL/6J-254	Private Strain	8	209	71.3	45	44.4
C57BL/6J	C57BL/6J-255	Private Strain	5	155	71.6	.	
C57BL/6J	C57BL/6J-256	Private Strain	5	188	22.3	.	
C57BL/6J	C57BL/6J-257	Private Strain	6	185	88.6	30	33.3
C57BL/6J	C57BL/6J-258	Private Strain	5	97	42.3	30	46.7
C57BL/6J	C57BL/6J-259	Private Strain	5	58	55.2	26	15.4
C57BL/6J	C57BL/6J-26	Private Strain	6	140	38.6	30	53.3
C57BL/6J	C57BL/6J-260	Private Strain	5	83	55.4	30	30.0
C57BL/6J	C57BL/6J-261	Private Strain	9	328	54.9	.	
C57BL/6J	C57BL/6J-262	Private Strain	6	217	86.2	30	50.0
C57BL/6J	C57BL/6J-263	Private Strain	5	55	83.6	30	43.3
C57BL/6J	C57BL/6J-264	Private Strain	5	32	87.5	28	39.3
C57BL/6J	C57BL/6J-265	Private Strain	8	203	50.7	45	48.9
C57BL/6J	C57BL/6J-266	Private Strain	5	158	58.9	.	
C57BL/6J	C57BL/6J-267	Private Strain	5	187	23.0	.	
C57BL/6J	C57BL/6J-268	Private Strain	5	173	47.4	.	
C57BL/6J	C57BL/6J-269	Private Strain	8	300	48.7	60	18.3
C57BL/6J	C57BL/6J-27	Private Strain	6	223	36.3	30	46.7
C57BL/6J	C57BL/6J-270	Private Strain	8	152	34.9	45	55.6
C57BL/6J	C57BL/6J-271	Private Strain	8	79	87.3	45	31.1
C57BL/6J	C57BL/6J-272	Private Strain	8	268	48.5	45	48.9
C57BL/6J	C57BL/6J-273	Private Strain	5	168	45.2	.	
C57BL/6J	C57BL/6J-274	Private Strain	5	215	42.8	.	
C57BL/6J	C57BL/6J-275	Private Strain	6	182	36.3	.	
C57BL/6J	C57BL/6J-276	Private Strain	4	66	21.2	.	
C57BL/6J	C57BL/6J-277	Private Strain	4	82	13.4	.	
C57BL/6J	C57BL/6J-278	Private Strain	8	261	28.7	.	
C57BL/6J	C57BL/6J-279	Private Strain	16	384	70.4	45	0.0
C57BL/6J	C57BL/6J-28	Private Strain	6	156	53.2	30	43.3
C57BL/6J	C57BL/6J-280	Private Strain	16	583	52.5	45	0.0
C57BL/6J	C57BL/6J-281	Private Strain	9	352	30.1	.	
C57BL/6J	C57BL/6J-282	Private Strain	6	129	54.3	.	
C57BL/6J	C57BL/6J-283	Private Strain	6	137	54.0	.	
C57BL/6J	C57BL/6J-284	Private Strain	8	212	58.5	.	
C57BL/6J	C57BL/6J-29	Private Strain	7	246	33.7	30	43.3
C57BL/6J	C57BL/6J-3	Private Strain	10	298	16.8	30	40.0
C57BL/6J	C57BL/6J-30	Private Strain	13	531	25.5	60	40.0
C57BL/6J	C57BL/6J-31	Private Strain	6	140	59.3	30	53.3
C57BL/6J	C57BL/6J-32	Private Strain	6	214	16.4	30	40.0
C57BL/6J	C57BL/6J-33	Private Strain	6	172	65.7	30	23.3
C57BL/6J	C57BL/6J-34	Private Strain	7	200	35.0	30	50.0
C57BL/6J	C57BL/6J-35	Private Strain	6	217	60.8	30	50.0
C57BL/6J	C57BL/6J-36	Private Strain	6	172	16.9	30	36.7
C57BL/6J	C57BL/6J-37	Private Strain	6	218	36.7	30	43.3
C57BL/6J	C57BL/6J-38	Private Strain	14	216	0.0	.	
C57BL/6J	C57BL/6J-39	Private Strain	6	273	17.2	30	16.7
C57BL/6J	C57BL/6J-4	Private Strain	6	150	40.7	30	46.7

C57BL/6J	C57BL/6J-40	Private Strain	5	185	55.1	30	26.7
C57BL/6J	C57BL/6J-41	Private Strain	6	183	44.3	30	40.0
C57BL/6J	C57BL/6J-42	Private Strain	5	124	70.2	.	
C57BL/6J	C57BL/6J-43	Private Strain	5	213	19.7	30	36.7
C57BL/6J	C57BL/6J-44	Private Strain	6	149	24.8	30	63.3
C57BL/6J	C57BL/6J-45	Private Strain	6	136	47.8	.	
C57BL/6J	C57BL/6J-46	Private Strain	5	120	58.3	.	
C57BL/6J	C57BL/6J-47	Private Strain	6	190	49.5	30	33.3
C57BL/6J	C57BL/6J-48	Private Strain	5	111	27.9	30	26.7
C57BL/6J	C57BL/6J-49	Private Strain	6	156	59.6	30	36.7
C57BL/6J	C57BL/6J-5	Private Strain	5	244	38.5	.	
C57BL/6J	C57BL/6J-50	Private Strain	6	222	13.1	29	34.5
C57BL/6J	C57BL/6J-51	Private Strain	6	221	31.7	30	53.3
C57BL/6J	C57BL/6J-52	Private Strain	6	181	33.1	30	53.3
C57BL/6J	C57BL/6J-53	Private Strain	6	124	34.7	30	40.0
C57BL/6J	C57BL/6J-54	Private Strain	6	161	18.0	30	23.3
C57BL/6J	C57BL/6J-55	Private Strain	6	143	18.2	25	52.0
C57BL/6J	C57BL/6J-56	Private Strain	6	171	23.4	30	53.3
C57BL/6J	C57BL/6J-57	Private Strain	6	157	23.6	30	46.7
C57BL/6J	C57BL/6J-58	Private Strain	12	211	55.0	30	20.0
C57BL/6J	C57BL/6J-59	Private Strain	6	208	49.0	30	33.3
C57BL/6J	C57BL/6J-6	Private Strain	5	227	24.7	.	
C57BL/6J	C57BL/6J-60	Private Strain	6	173	32.4	30	53.3
C57BL/6J	C57BL/6J-61	Private Strain	6	234	29.1	30	46.7
C57BL/6J	C57BL/6J-62	Private Strain	6	276	26.1	30	63.3
C57BL/6J	C57BL/6J-63	Private Strain	6	227	32.6	30	40.0
C57BL/6J	C57BL/6J-64	Private Strain	6	239	18.0	30	40.0
C57BL/6J	C57BL/6J-65	Private Strain	8	300	12.3	30	50.0
C57BL/6J	C57BL/6J-66	Private Strain	6	216	51.4	30	30.0
C57BL/6J	C57BL/6J-67	Private Strain	6	201	32.3	30	43.3
C57BL/6J	C57BL/6J-68	Private Strain	6	192	8.9	15	20.0
C57BL/6J	C57BL/6J-69	Private Strain	6	188	39.9	30	40.0
C57BL/6J	C57BL/6J-7	Private Strain	5	258	16.7	.	
C57BL/6J	C57BL/6J-70	Private Strain	6	237	27.0	30	26.7
C57BL/6J	C57BL/6J-71	Private Strain	6	277	9.4	26	46.2
C57BL/6J	C57BL/6J-72	Private Strain	6	199	49.2	30	46.7
C57BL/6J	C57BL/6J-73	Private Strain	5	181	29.3	30	56.7
C57BL/6J	C57BL/6J-74	Private Strain	6	244	32.0	30	50.0
C57BL/6J	C57BL/6J-75	Private Strain	13	256	36.9	51	23.5
C57BL/6J	C57BL/6J-76	Private Strain	6	175	48.0	.	
C57BL/6J	C57BL/6J-77	Private Strain	6	228	17.1	30	40.0
C57BL/6J	C57BL/6J-78	Private Strain	6	113	69.0	30	0.0
C57BL/6J	C57BL/6J-79	Private Strain	6	186	28.5	30	33.3
C57BL/6J	C57BL/6J-8	Private Strain	5	134	23.9	.	
C57BL/6J	C57BL/6J-80	Private Strain	6	226	23.0	30	46.7
C57BL/6J	C57BL/6J-81	Private Strain	12	392	46.8	30	30.0
C57BL/6J	C57BL/6J-82	Private Strain	5	144	6.3	30	23.3
C57BL/6J	C57BL/6J-83	Private Strain	5	237	19.0	30	46.7
C57BL/6J	C57BL/6J-84	Private Strain	6	225	9.3	21	38.1
C57BL/6J	C57BL/6J-85	Private Strain	5	65	81.5	30	43.3
C57BL/6J	C57BL/6J-86	Private Strain	6	124	25.0	30	33.3
C57BL/6J	C57BL/6J-87	Private Strain	7	161	60.9	30	36.7
C57BL/6J	C57BL/6J-88	Private Strain	6	167	23.4	.	
C57BL/6J	C57BL/6J-89	Private Strain	6	222	11.3	25	40.0
C57BL/6J	C57BL/6J-9	Private Strain	5	187	25.1	.	
C57BL/6J	C57BL/6J-90	Private Strain	6	202	46.0	30	30.0
C57BL/6J	C57BL/6J-91	Private Strain	21	777	31.4	135	26.7
C57BL/6J	C57BL/6J-92	Private Strain	6	159	53.5	30	33.3
C57BL/6J	C57BL/6J-93	Private Strain	6	193	43.0	30	33.3
C57BL/6J	C57BL/6J-94	Private Strain	5	163	37.4	30	43.3
C57BL/6J	C57BL/6J-95	Private Strain	6	125	14.4	18	66.7
C57BL/6J	C57BL/6J-96	Private Strain	6	152	19.7	30	23.3
C57BL/6J	C57BL/6J-97	Private Strain	5	181	49.7	30	33.3
C57BL/6J	C57BL/6J-98	Private Strain	6	168	38.1	30	63.3
C57BL/6J	C57BL/6J-99	Private Strain	5	134	33.6	30	50.0
C57BL/6J	6001	STOCK Dicer1<tm1Bdh>/J	6	217	59.0	30	46.7
C57BL/6J	6241	STOCK Hhip<tm1Amc>/J	5	142	57.7	.	
C57BL/6J	5994	STOCK Mbtps1<tm1Jdh>/J	5	108	54.6	30	46.7
C57BL/6J	6706	STOCK Ncam2<tm1Mom>/MomJ	6	204	33.3	30	40.0
C57BL/6J	6951	STOCK Notch1<tm2Rko>/GridJ	5	171	14.6	.	
C57BL/6J	6697	STOCK Nrp2<tm1.1Mom>/MomJ	14	380	67.6	60	38.3
C57BL/6J	6700	STOCK Nrp2<tm1.2Mom>/MomJ	5	169	78.1	30	66.7
C57BL/6J	6702	STOCK Nts<tm1Mom>/MomJ	5	181	21.5	30	40.0
C57BL/6J	6622	STOCK Olfr151<tm10Mom>/MomJ	5	188	58.0	30	43.3
C57BL/6J	6646	STOCK Olfr151<tm11(Olfr160)Mom>/MomJ	7	128	74.2	30	56.7
C57BL/6J	6645	STOCK Olfr151<tm12(Olfr16)Mom>/MomJ	6	138	68.8	30	43.3
C57BL/6J	6691	STOCK Olfr151<tm14(Adrb2)Mom>/MomJ	6	205	72.2	30	53.3
C57BL/6J	6635	STOCK Olfr151<tm15(V1rb2)Mom>/MomJ	6	223	21.5	30	60.0
C57BL/6J	6692	STOCK Olfr151<tm16(Olfr160/161)Mom>/MomJ	6	172	40.7	30	43.3
C57BL/6J	6693	STOCK Olfr151<tm17Mom>/MomJ	6	155	9.7	14	50.0
C57BL/6J	6688	STOCK Olfr151<tm18(GFP)Mom>/MomJ	11	400	42.0	90	36.7
C57BL/6J	6630	STOCK Olfr151<tm1Mom>/MomJ	5	139	28.1	30	40.0
C57BL/6J	6694	STOCK Olfr151<tm20Mom>/MomJ	6	168	44.0	30	46.7
C57BL/6J	6695	STOCK Olfr151<tm21Mom>/MomJ	6	173	67.1	43	27.9
C57BL/6J	6690	STOCK Olfr151<tm23Mom>/MomJ	6	230	84.3	30	63.3
C57BL/6J	6677	STOCK Olfr151<tm28Mom>/MomJ	11	369	34.9	60	28.3
C57BL/6J	6629	STOCK Olfr151<tm2Mom>/MomJ	6	115	37.4	30	23.3
C57BL/6J	6628	STOCK Olfr151<tm3Mom>/MomJ	6	115	87.0	30	40.0
C57BL/6J	6627	STOCK Olfr151<tm4Mom>/MomJ	5	173	46.8	30	56.7
C57BL/6J	6626	STOCK Olfr151<tm6Mom>/MomJ	5	102	54.9	30	43.3
C57BL/6J	6625	STOCK Olfr151<tm7Mom>/MomJ	5	98	87.8	30	46.7
C57BL/6J	6624	STOCK Olfr151<tm8Mom>/MomJ	6	162	50.6	30	16.7
C57BL/6J	6623	STOCK Olfr151<tm9Mom>/MomJ	5	249	73.9	30	60.0
C57BL/6J	6638	STOCK Olfr155<tm2Mom>/MomJ	5	174	56.9	30	56.7
C57BL/6J	6639	STOCK Olfr156<tm1Mom>/MomJ	5	197	76.1	30	56.7
C57BL/6J	6641	STOCK Olfr157<tm2Mom>/MomJ	5	136	52.9	30	63.3

C57BL/6J	6740	STOCK Olfr160<tm1Mom> Tg(Olfr151,taulacZ)AMom/MomJ	5	160	54.4	.	
C57BL/6J	6741	STOCK Olfr160<tm1Mom> Tg(Olfr151,taulacZ)BMom/MomJ	5	171	15.8	27	14.8
C57BL/6J	6647	STOCK Olfr160<tm1Mom>/MomJ	5	149	59.7	30	46.7
C57BL/6J	6652	STOCK Olfr160<tm2(GFP)Mom>/MomJ	6	173	23.1	30	43.3
C57BL/6J	6696	STOCK Olfr160<tm3Mom>/MomJ	6	148	39.9	30	50.0
C57BL/6J	6636	STOCK Olfr160<tm5(Cnga2)Mom>/MomJ	5	192	68.2	30	13.3
C57BL/6J	6678	STOCK Olfr160<tm6Mom>/MomJ	12	383	24.8	60	33.3
C57BL/6J	6650	STOCK Olfr17<tm6Mom>/MomJ	10	166	0.0	3	66.7
C57BL/6J	6669	STOCK Olfr17<tm7Mom>/MomJ	6	146	41.1	30	43.3
C57BL/6J	6686	STOCK Olfr6<tm1Mom>/MomJ	7	211	35.1	30	30.0
C57BL/6J	6683	STOCK Olfr713<tm1Mom>/MomJ	5	87	21.8	16	81.3
C57BL/6J	6684	STOCK Olfr713<tm2Mom>/MomJ	12	110	4.5	5	100.0
C57BL/6J	6685	STOCK Olfr714<tm1Mom>/MomJ	5	65	81.5	30	53.3
C57BL/6J	5645	STOCK Tg(ACTB-mRFP1)1F1Hadj/J	6	214	79.9	30	20.0
C57BL/6J	3529	STOCK Tg(NES-TVA)12Hev/J	7	214	68.7	30	13.3
C57BL/6J	6674	STOCK Tg(Olfr16,taulacZ)2030Mom/MomJ	6	178	41.6	30	43.3
C57BL/6J	3273	STOCK Tg(rtTAhCMV)4Bjd/J	8	135	61.5	30	36.7
C57BL/6J	6631	STOCK V1rb2<tm1Mom>/MomJ	5	212	71.2	30	26.7
C57BL/6J	6632	STOCK V1rb2<tm2Mom>/MomJ	10	214	5.5	9	0.0
C57BL/6J	6634	STOCK V1rb2<tm4(Olfr151)Mom>/MomJ	5	113	72.6	30	46.7
C57BL/6J	6657	STOCK Vmn2r26<tm1Mom>/MomJ	5	140	83.6	30	20.0
C57BL/6J	6651	STOCK-Olfr17<tm4Mom>/MomJ	5	150	36.7	30	46.7
C57BL/6J	2307	WB.Cg-an/BrkJ	6	109	24.8	26	30.8
C57BLKS/J	3606	BKS(Cg)-frzl/J	8	143	20.3	29	20.7
C57BLKS/J	5898	BKS(Cg)-trls/J	8	179	12.3	24	8.3
C57BLKS/J	4176	BKS.B6-Tub<tub>/Jng	10	99	22.2	22	36.4
C57BLKS/J	2391	BKSChpLt.HRS-Cpe<fat>/J	10	100	30.0	30	26.7
C57BLKS/J	2760	C57BLKS/J-Npc1<spm>/J	9	156	16.0	25	48.0
DBA/2J	6811	D2.129(Cg)-Lhx9<tm1Lmgd>/EiJ	6	102	95.1	30	53.3
DBA/2J	7042	D2.129P2(B6)-Nr5a1<tm2Klp>/EiJ	5	76	89.5	30	43.3
DBA/2J	6003	D2.129P2(Cg)-Cbx2<tm1Cim>/EiJ	6	135	84.4	30	53.3
DBA/2J	6817	D2.129S4(Cg)-Wt1<tm1Jae>/EiJ	17	468	85.4	90	22.2
DBA/2J	6808	D2.129S6(Cg)-Gata4<tm1.1Sho>/EiJ	10	249	68.3	30	26.7
DBA/2J	6768	D2.Cg-Tg(Myh6-Zfpm2)1Sho/EiJ	6	126	72.2	30	26.7
DBA/2J	6838	D2.Cg-Zfpm2<tm1Sho>/EiJ	8	78	92.3	30	36.7
DBA/2J	6818	DBA/2J-Chr Y<AKR/J>/EiJ	6	143	83.2	30	26.7
DBA/2J	DBA/2J-1	Private Strain	8	231	85.3	30	40.0
DBA/2J	DBA/2J-2	Private Strain	18	564	64.9	60	28.3
DBA/2J	DBA/2J-3	Private Strain	10	193	76.7	30	40.0
FVB/NJ	2935	FVB.129S2(B6)-Ccnd1<tm1Wbg>/J	20	292	77.6	75	46.7
FVB/NJ	2329	FVB.129S2-Plau<tm1Mlg>/J	10	176	86.4	30	46.7
FVB/NJ	3502	FVB.129S6-Stat5a<tm1Mam>/J	20	252	87.2	105	29.5
FVB/NJ	4363	FVB.Cg-Tg(MMTV-vHaras)SH1Led/J	15	188	46.9	60	8.3
FVB/NJ	3170	FVB.Cg-Tg(Myh6-tTA)6Smbf/J	40	618	8.1	43	37.2
FVB/NJ	6225	FVB.Cg-Tg(SFTPC-rtTA)5Jaw/J	10	117	90.6	30	30.0
FVB/NJ	6224	FVB.Cg-Tg(tetO-cre)1Jaw/J	5	62	96.8	30	10.0
FVB/NJ	6402	FVB/N-Adrb3<tm1Lowl>/J	8	122	83.6	.	
FVB/NJ	3640	FVB/NJ-Tg(YAC72)2511Hay/J	22	277	78.8	120	16.7
FVB/NJ	3257	FVB/N-Tg(GFAPGFP)14Mes/J	20	356	45.5	30	30.0
FVB/NJ	2421	FVB/N-Tg(MtTGFA)100Lmb/J	10	161	96.3	30	40.0
FVB/NJ	4066	FVB;129S-Men1<tm1.1Ctre>/J	10	77	62.3	30	36.7
FVB/NJ	5110	FVB-Tg(Sod1*G86R)M1Jwg/J	10	134	29.9	25	48.0
FVB/NJ	FVB/NJ-1	Private Strain	10	104	99.0	30	30.0
FVB/NJ	FVB/NJ-10	Private Strain	6	49	81.6	.	
FVB/NJ	FVB/NJ-11	Private Strain	10	92	0.0	.	
FVB/NJ	FVB/NJ-12	Private Strain	10	140	6.4	.	
FVB/NJ	FVB/NJ-13	Private Strain	10	78	7.7	.	
FVB/NJ	FVB/NJ-14	Private Strain	10	76	57.9	.	
FVB/NJ	FVB/NJ-15	Private Strain	10	132	92.4	.	
FVB/NJ	FVB/NJ-16	Private Strain	10	185	75.7	.	
FVB/NJ	FVB/NJ-2	Private Strain	10	200	81.0	30	56.7
FVB/NJ	FVB/NJ-3	Private Strain	8	62	79.0	30	33.3
FVB/NJ	FVB/NJ-4	Private Strain	10	113	96.5	30	16.7
FVB/NJ	FVB/NJ-5	Private Strain	10	93	98.9	30	16.7
FVB/NJ	FVB/NJ-6	Private Strain	8	106	90.6	.	
FVB/NJ	FVB/NJ-7	Private Strain	30	400	56.0	60	26.7
FVB/NJ	FVB/NJ-8	Private Strain	30	420	56.0	60	30.0
FVB/NJ	FVB/NJ-9	Private Strain	5	92	73.9	30	26.7
FVB/NJ	2326	STOCK Plat<tm1Mlg>/J	10	144	73.6	30	33.3
NOD/ShiLtJ	6355	NOD.129-Btla<tm1Kmm>/J	10	163	62.6	.	.
NOD/ShiLtJ	5080	NOD.129-Ica1<tm1Mdos>/MdosJ	10	290	70.7	30	56.7
NOD/ShiLtJ	6360	NOD.129S2(B6)-Aire<tm1.1Doi>/DoiJ	10	144	46.5	30	56.7
NOD/ShiLtJ	4761	NOD.129S2(B6)-Cd28<tm1Mak>/JbsJ	5	54	90.7	30	76.7
NOD/ShiLtJ	5983	NOD.129S4(B6)-Icam1<tm1Jcgr>/SmtnJ	8	130	92.3	30	36.7
NOD/ShiLtJ	6610	NOD.B6-Tg(ML5sHEL)5Ccg/DvsJ	20	425	61.3	60	30.0
NOD/ShiLtJ	5870	NOD/ShiLt(Cg)-Tg(Ins2-GAD2)2Lt/J	10	131	71.8	.	.
NOD/ShiLtJ	6604	NOD/ShiLtDvs-Tg(HLA-A/H2-D/B2M)1Dvs/J	10	173	22.5	30	56.7
NOD/ShiLtJ	4939	NOD/ShiLtJ-Lepr<db-5J>/LtJ	10	168	32.1	30	30.0
NOD/ShiLtJ	4986	NOD/ShiLt-Tg(Ins2-cre)3Lt/Lt	8	138	82.6	30	53.3
NOD/ShiLtJ	4987	NOD/ShiLt-Tg(Ins2-cre)6Lt/Lt	8	165	61.2	30	40.0
