# Supplementary material for: Conserving, Distributing and Managing Genetically Modified Mouse Lines by Sperm Cryopreservation
Source: PLoS One. 2008 Jul 30;3(7):e2792. doi: 10.1371/journal.pone.0002792 (PMC2453316; doi:10.1371/journal.pone.0002792)
Supplement: Table S2 — Data set for freshly collected sperm and cryopreserved embryos. (0.06 MB PDF) [file pone.0002792.s004.pdf]

Table S2. Data set for f*reshly collected sperm and cryopreserved embryos.*

The first row in the table details the data within each column. The first column depicts the inbred strain that was selected for oocyte donation. The second column represents the stock/accession number for each strain. For public strains, the stock number can be used to obtain detailed strain information by searching the JAX® Mice Database (http://jaxmice.jax.org/query/f?p=205:1:1867898574872980466). Accession numbers were randomly created for private strains, to maintain confidentiality. The third column shows the strain name, which details the genetic background and genetic modification of the strain (http://www.informatics.jax.org/mgihome/nomen/).

The fourth, fifth, and sixth columns indicate the number of females and oocytes used for *in vitro* fertilization and the proportion of oocytes developing into 2-cell embryos for that strain. Columns seven and eight detail the number of embryos transferred and the proportion of these developing into liveborn for the given strain. Data are not provided for all rows in columns seven and eight, as a subset of strains was used for embryo transfer.

Oocyte Donor	Stock / Acc #	Strain Name	# females	# oocytes	% 2-cell	# embryos transferred	% liveborn
129S1/SvImJ	6616	129(B6)-Ccni<tm1Jro>/J	10	262	0.0		
129S1/SvImJ	6367	129-Cckar<tm1Kpn>/J	10	192	9.4		
129S1/SvImJ	6369	129-Cckbr<tm1Kpn>/J	10	130	34.6		
129S1/SvImJ	6067	129-Gt(ROSA)26Sor<tm2Luo>/J	40	1027	10.4		
129S1/SvImJ	4446	129-Lta4h<tm1Bhk>/J	230	6251	14.3		
129S1/SvImJ	6403	129S.B6-Tg(KRT14-Esr1/HRAS)1Pkha/J	25	863	9.4		
129S1/SvImJ	6661	129S.B6-Tg(KRT14-RAF1/ESR1)1Pkha/J	25	500	7.8		
129S1/SvImJ	6409	129S1.Cg-Tg(APPSw)40Btla/J	12	278	18.3		
129S1/SvImJ	2448	129S1/SvImJ	120	2915	24.3	110	55.0
129S1/SvImJ	6821	129S-Catsper4<tm1Clph>/J	21	380	41.3		
129S1/SvImJ	7005	129S-Scg5<tm1Led>/J	12	206	23.8		
129S1/SvImJ	7199	129S-Sgpl1<Gt(ROSA)78Sor>/J	10	298	10.1		
129S1/SvImJ	7204	B6;129S4-2610005L07Rik<Gt(ROSA)73Sor>/J	10	193	36.3		
129S1/SvImJ	129S1/SvImJ-17	Private Strain	15	382	11.8		
129S1/SvImJ	129S1/SvImJ-4	Private Strain	10	294	15.3		
129S1/SvImJ	6085	STOCK Rad9<tm1Lieb>/J	145	4510	14.2	376	41.2
129S1/SvImJ	4510	STOCK Rom1<tm1Mci>/J	20	459	9.1		
129S1/SvImJ	6473	STOCK Smyd1<tm1Dsr>/J	10	192	25.0		
129S1/SvImJ	6850	STOCK Tg(ACTB-Bgeo,-NOTCH1,-EGFP)1Lbe/J	15	412	10.7		
129X1/SvJ	2908	129-Col4a3<tm1Dec>/J	190	2795	40.5	284	20.6
129X1/SvJ	6050	129-Sirt6<tm1Fwa>/J	30	527	39.2	.	
129X1/SvJ	5291	129-Tulp3<tm1Jng>/Pjn	20	407	18.2	.	
129X1/SvJ	129X1/SvJ-4	Private Strain	40	784	5.9	50	60.0
129X1/SvJ	129X1/SvJ-6	Private Strain	80	1669	12.5	50	24.0
129X1/SvJ	7576	STOCK Gt(ROSA)26Sor<tm4(ACTB-tdTomato,-EGFP)Luo>/J	15	582	40.7	.	
B6129SF1/J	4177	B6;129-Cd3e<tm1Lov>/J	35	911	36.6		
B6129SF1/J	3535	B6;129-Gla<tm1Kul>/J	40	871	72.7	30	60.0
B6129SF1/J	6088	B6;129-Mcl1<tm3Sjk>/J	10	189	79.4		
B6129SF1/J	5323	B6;129P2 Pemt<tm1J>-tnyw/J	55	996	55.7		
B6129SF1/J	5549	B6;129-Pax3<tm1(cre)Joe>/J		409	55.3		
B6129SF1/J	4858	B6;129S1-Tshr<tm1Rmar>/J	40	965	6.8		
B6129SF1/J	2495	B6;129S4-Col1a1<tm1Jae>/J	45	1186	43.1	226	41.1
B6129SF1/J	5937	B6;129S4-Ucp3<tm1Lowl>/J	30	849	16.1	60	41.7
B6129SF1/J	3524	B6;129S6-Lrp8<tm1Her>/J	10	189	51.4	534	35.0
B6129SF1/J	2536	B6;129S-Btk<tm1Wk>/J	15	427	22.0	163	35.3
B6129SF1/J	2201	B6;129S-Gja1<tm1Kdr>/J	30	972	69.5	265	7.7
B6129SF1/J	3807	B6;129S-Sele<tm1Hyn> Sell<tm1Hyn> Selp<tm1Hyn>/J	41	684	57.2	98	19.0
B6129SF1/J	B6129SF1/J-16	Private Strain	10	279	65.2		
B6129SF1/J	4779	STOCK Mapt<tm1(EGFP)Klt>/J	70	2061	22.1		
B6129SF1/J	5936	STOCK Tg(ACTA1-cre)79Jme/J	61	1147	78.1	118	58.5
B6129SF1/J	3920	STOCK Tg(ACTB-Bgeo/GFP)21Lbe/J	50	828	98.7		
B6129SF1/J	3102	STOCK Tgfb2<tm1Doe>/J	55	1128	58.6	134	32.5
BALB/cByJ	5520	B6;CByJ-Cacna1a<tg-6J>/J	20	295	74.6		
BALB/cByJ	5050	B6;CByJ-Cacna2d2<du-3J>/J	59	1053	32.6		
BALB/cByJ	5633	B6;CByJ-Dst<dt-38J>/J	50	1111	88.6		
BALB/cByJ	5750	B6;CByJ-nmf375/J	60	1198	66.1	166	31.3
BALB/cByJ	5636	B6;CByJ-nmf418/J	59	1095	67.0		
BALB/cByJ	5751	B6;CByJ-nmf419/J	35	838	82.6	34	44.1
BALB/cByJ	5463	B6;CByJ-Scn8a<7J>/J	90	1857	26.3		
BALB/cByJ	5753	B6;CByJ-Spnb4<qv-9J>/J	60	672	85.7	152	12.5
BALB/cByJ	4317	BALB/cBy-Gulo<sfx>/J	20	614	41.2		
BALB/cByJ	1265	BALB/cByJ-Clcn1<adr-mto2J>/J	178	3145	54.8	176	9.1
BALB/cByJ	3756	BALB/cByJ-nr/J	139	1996	55.3	204	23.5
BALB/cByJ	3478	C.129P2-Cbx2<tm1Cim>/J	158	2588	40.0	784	12.1
BALB/cByJ	5419	C;129P2-Npy5r<tm1Pern>/J	59	591	77.9		
BALB/cByJ	3581	CBy.129S4-Dab1<tm1Cpr>/J	48	499	8.2		
BALB/cByJ	2766	CBy.MRL-Fbxw4<Dac-2J>/J	107	1640	65.0	207	18.3
BALB/cByJ	5421	CBy;B6-Bmp5<cfe-se8J>/J	50	1125	81.1		
BALB/cByJ	1723	CByJ.A-Ttc7<fsn>/J	10	71	39.4		
BALB/cByJ	BALB/cByJ-25	Private Strain	10	163	63.2		
BALB/cByJ	BALB/cByJ-26	Private Strain	20	236	63.1		
BALB/cByJ	BALB/cByJ-27	Private Strain	29	628	85.0		
BALB/cByJ	BALB/cByJ-28	Private Strain	40	704	90.9	29	20.7
BALB/cByJ	BALB/cByJ-29	Private Strain	68	1366	57.2	80	15.0
BALB/cByJ	BALB/cByJ-30	Private Strain	20	393	0.5		
BALB/cByJ	BALB/cByJ-31	Private Strain	10	196	57.7		
BALB/cByJ	BALB/cByJ-32	Private Strain	15	251	37.8		
BALB/cByJ	BALB/cByJ-33	Private Strain	45	866	84.5	32	31.3
BALB/cByJ	BALB/cByJ-34	Private Strain	86	1293	7.0	72	41.3
BALB/cByJ	BALB/cByJ-35	Private Strain	45	911	86.2	69	21.8
BALB/cByJ	BALB/cByJ-36	Private Strain	45	584	86.5	21	57.1
BALB/cByJ	BALBc/ByJ-5	Private Strain	10	341	81.2		
BALB/cJ	3092	BALB/cNctr-Npc1<m1N>/J	70	629	67.0		
BALB/cJ	6339	C.129-Btla<tm1Kmm>/J	15	215	45.6		
BALB/cJ	4190	C.129-Il4<tm1Lky>/J	179	2579	22.8		
BALB/cJ	5700	C.129P2-Cxcr6<tm1Litt>/J	80	1135	83.2		
BALB/cJ	2691	C.129S1(B6)-Il12a<tm1Jm>/J	40	696	49.4	26	38.5
BALB/cJ	2694	C.129S1-Il12b<tm1Jm>/J	41	516	64.5		
BALB/cJ	2724	C.129S2(B6)-Il8rb<tm1Mwm>/J	150	2586	30.3		
BALB/cJ	5629	C.129S2-Fcer1a<tm1Knt>/J	90	1176	30.4		
BALB/cJ	6845	C.129S4(B6)-C5ar1<tm1Cge>/J	20	264	48.9		
BALB/cJ	5712	C.129S4-C3ar1<tm1Cge>/J	70	1353	47.6		
BALB/cJ	5440	C.129S4-Ccr3<tm1Cge>/J	39	479	1.5		
BALB/cJ	6769	C.Cg-Foxp3<tm2Tch>/J	20	264	35.6		
BALB/cJ	5673	C.Cg-Tg(Mx1-cre)1Cgn/J	30	506	11.7		
BALB/cJ	5420	C;129S7 Gt(ROSA)26Sor-Bmp5<cfe-se7J>/J	70	886	32.2		
BALB/cJ	4223	CHa.SWV(C3Fe)-Mbp<shi>/J	40	531	52.5		
BALB/cJ	BALB/cJ-1	Private Strain	79	917	44.0	546	6.8
BALB/cJ	BALB/CJ-15	Private Strain	100	935	60.2		
BALB/cJ	BALB/CJ-16	Private Strain	10	54	37.0		

BALB/cJ	BALB/CJ-17	Private Strain	110	915	59.3	96	34.4
BALB/cJ	BALB/CJ-18	Private Strain	74	955	63.8	46	30.4
BALB/cJ	BALB/CJ-19	Private Strain	73	948	61.4	82	24.0
BALB/cJ	BALB/CJ-20	Private Strain	75	1068	60.4	240	18.8
BALB/cJ	BALB/CJ-21	Private Strain	73	1437	44.3	94	4.2
BALB/cJ	BALB/CJ-22	Private Strain	80	1347	87.0	150	13.2
BALB/cJ	BALB/CJ-23	Private Strain	20	329	39.5		
BALB/cJ	BALB/CJ-24	Private Strain	49	567	37.3		
BALB/cJ	BALB/CJ-25	Private Strain	80	1319	48.5	38	36.8
BALB/cJ	BALB/CJ-26	Private Strain	94	1145	22.5	102	8.8
C3H/HeJ	C3H/HeJ-2	Private Strain	40	607	77.1		
C3H/HeJ	C3H/HeJ-3	Private Strain	60	853	84.2	62	45.2
C3H/HeJ	C3H/HeJ-4	Private Strain	80	871	64.3	42	38.1
C3HeB/FeJ	4951	C3HeB/FeJ-Cacnb4<lh-3J>/J	35	437	71.4		
C3HeB/FeJ	1533	C3HeB/FeJ-Mc1r<E-so> Gli3<Xt-J>/J	30	423	67.4	76	2.6
C3HeB/FeJ	6045	C3HeB/FeJ-Xsl/J	40	598	88.3	78	23.3
C3HeB/FeJ	C3HeB/FeJ-14	Private Strain	30	450	73.3		
C3HeB/FeJ	6128	STOCK Otof<deaf5Jcs>/Kjn	40	510	89.2	22	50.0
C57BL/6J	6963	B6(101)-Mdh1<am1H>/LvtJ	6	203	55.2	.	.
C57BL/6J	4742	B6(Cg)-Ncf1<m1J>/J	10	291	65.6	.	.
C57BL/6J	7212	B6(Cg)-Tnfrsf13c<tm1Mass>/J	10	239	77.0	.	.
C57BL/6J	5624	B6(V) Lep<ob>-whe/J	20	682	61.9	.	.
C57BL/6J	7248	B6.129(FVB)-Col1a2<tm1Mcbr>/J	8	123	95.1	.	.
C57BL/6J	2831	B6.129-Ahr<tm1Bra>/J	48	862	85.2	224	32.4
C57BL/6J	6257	B6.129-Aldh5a1<tm1Kmg>/J	10	208	59.6	.	.
C57BL/6J	5708	B6.129-Apbb1<tm1Quhu>/J	30	693	79.4	.	.
C57BL/6J	6353	B6.129-Btla<tm1Kmm>/J	9	286	64.3	.	.
C57BL/6J	5795	B6.129-Ccrl2<tm1Dgen>/J	105	3169	47.3	68	17.6
C57BL/6J	5319	B6.129-Cdh1<tm2Kem>/J	45	1127	74.2	.	.
C57BL/6J	5789	B6.129-Cer1<tm1Dgen>/J	40	1369	69.8	81	5.0
C57BL/6J	5951	B6.129-Dgat2<tm1Rvf>/J	30	1017	80.0	39	28.2
C57BL/6J	5628	B6.129-Emx1<tm1(cre)Krj>/J	30	1407	82.3	.	.
C57BL/6J	5704	B6.129-Fbn1<tm2Rmz>/J	8	179	83.8	.	.
C57BL/6J	6874	B6.129-Gabra4<tm1.2Geh>/J	6	148	89.2	.	.
C57BL/6J	3725	B6.129-Gabrd<tm1Geh>/J	35	882	58.3	184	29.3
C57BL/6J	6411	B6.129-Gast<tm1(INS)Ez>/J	15	438	86.5	.	.
C57BL/6J	6080	B6.129-Gt(ROSA)26Sor<tm2Luo>/J	15	518	61.0	.	.
C57BL/6J	7561	B6.129-Hif1a<tm3Rsjo>/J	8	160	85.6	.	.
C57BL/6J	5768	B6.129-Htr5a<tm1Dgen>/J	85	2933	61.1	66	24.2
C57BL/6J	6412	B6.129-Il12b<tm1Lky>/J	10	308	38.6	.	.
C57BL/6J	5867	B6.129-Indo<tm1Alm>/J	40	1725	70.0	34	23.5
C57BL/6J	5819	B6.129-Insl5<tm1Dgen>/J	57	1566	52.0	96	31.4
C57BL/6J	7251	B6.129-Mapt<tm1Hnd>/J	6	219	53.9	.	.
C57BL/6J	6785	B6.129P2(C)-Cd19<tm1(cre)Cgn>/J	6	121	43.0	.	.
C57BL/6J	3890	B6.129P2(C)-Mecp2<tm1.1Bird>/J	83	2218	28.9	348	33.9
C57BL/6J	7453	B6.129P2(Cg)-Dhcr7<tm1Gst>/J	6	78	32.1	.	.
C57BL/6J	5772	B6.129P2-Acvrl1<tm1Dgen>/J	61	2108	53.8	58	34.5
C57BL/6J	6431	B6.129P2-Adam21<tm1Dgen>/J	81	3281	42.5	118	40.6
C57BL/6J	5770	B6.129P2-Adamts4<tm1Dgen>/J	95	2900	55.2	363	23.5
C57BL/6J	5771	B6.129P2-Adamts5<tm1Dgen>/J	56	1762	44.9	148	24.0
C57BL/6J	5773	B6.129P2-Adcy3<tm1Dgen>/J	65	1630	76.5	144	24.1
C57BL/6J	5774	B6.129P2-Adcy7<tm1Dgen>/J	58	1577	44.9	58	34.5
C57BL/6J	5775	B6.129P2-Adipor2<tm1Dgen>/J	80	2345	38.7	70	34.3
C57BL/6J	2681	B6.129P2-Agt<tm1Unc>/J	50	1967	58.1	123	12.8
C57BL/6J	2682	B6.129P2-Agtr1a<tm1Unc>/J	12	388	63.7	.	.
C57BL/6J	6433	B6.129P2-Atp8a1<tm1Dgen>/J	60	1658	81.9	48	33.3
C57BL/6J	5776	B6.129P2-Avpr1a<tm1Dgen>/J	44	1513	51.6	67	42.3
C57BL/6J	5777	B6.129P2-Axl<tm1Dgen>/J	106	2966	55.1	84	15.3
C57BL/6J	5783	B6.129P2-Cacna1c<tm1Dgen>/J	54	1751	70.5	70	25.7
C57BL/6J	5780	B6.129P2-Cacna2d3<tm1Dgen>/J	96	3139	26.7	81	25.9
C57BL/6J	5781	B6.129P2-Cacng3<tm1Dgen>/J	55	1933	32.5	28	50.0
C57BL/6J	5782	B6.129P2-Cacng4<tm1Dgen>/J	85	2248	50.9	48	25.0
C57BL/6J	5784	B6.129P2-Capn5<tm1Dgen>/J	86	3013	49.5	52	19.2
C57BL/6J	5785	B6.129P2-Capn7<tm1Dgen>/J	43	1871	63.4	40	27.5
C57BL/6J	5792	B6.129P2-Ccr1l1<tm1Dgen>/J	70	2122	59.3	74	32.4
C57BL/6J	5793	B6.129P2-Ccr6<tm1Dgen>/J	55	1299	49.4	28	35.7
C57BL/6J	5794	B6.129P2-Ccr7<tm1Dgen>/J	93	2723	47.7	63	66.7
C57BL/6J	5779	B6.129P2-Celsr2<tm1Dgen>/J	94	2732	65.3	92	17.4
C57BL/6J	5797	B6.129P2-Chrna2<tm1Dgen>/J	68	2480	52.6	46	26.1
C57BL/6J	7566	B6.129P2-Clip2<tm1.1Gal>/J	10	257	93.0	.	.
C57BL/6J	5786	B6.129P2-Cnr2<tm1Dgen>/J	45	1053	52.0	25	48.0
C57BL/6J	5787	B6.129P2-Ctsc<tm1Dgen>/J	40	1659	67.5	34	32.4
C57BL/6J	5796	B6.129P2-Cxcr3<tm1Dgen>/J	70	2802	42.5	64	21.9
C57BL/6J	5798	B6.129P2-Drd5<tm1Dgen>/J	83	2492	61.0	42	19.0
C57BL/6J	5799	B6.129P2-Edg6<tm1Dgen>/J	65	1562	54.7	100	40.0
C57BL/6J	5800	B6.129P2-Efemp2<tm1Dgen>/J	92	2438	44.9	50	28.0
C57BL/6J	4745	B6.129P2-Esr2<tm1Unc>/J	40	950	64.0	.	.
C57BL/6J	5801	B6.129P2-Esrra<tm1Dgen>/J	71	1679	62.6	120	40.6
C57BL/6J	4303	B6.129P2-F9<tm1Dws>/J	92	2255	59.7	.	.
C57BL/6J	5802	B6.129P2-Faim2<tm1Dgen>/J	50	1478	37.4	30	46.7
C57BL/6J	6262	B6.129P2-Fut2<tm1Sdo>/J	6	143	73.4	.	.
C57BL/6J	5803	B6.129P2-Fzd1<tm1Dgen>/J	54	1343	44.8	21	47.6
C57BL/6J	5804	B6.129P2-Fzd8<tm1Dgen>/J	44	1306	54.7	31	38.7
C57BL/6J	5811	B6.129P2-Gabra3<tm1Dgen>/J	60	2237	56.7	72	27.8
C57BL/6J	5812	B6.129P2-Gabra4<tm1Dgen>/J	50	1074	57.6	52	42.3
C57BL/6J	5810	B6.129P2-Gabrp<tm1Dgen>/J	50	1512	72.3	32	40.6
C57BL/6J	5809	B6.129P2-Galr1<tm1Dgen>/J	77	2932	44.2	114	27.8
C57BL/6J	5816	B6.129P2-Glra3<tm1Dgen>/J	85	2078	64.9	72	16.7
C57BL/6J	5805	B6.129P2-Gpr151<tm1Dgen>/J	65	1825	62.5	324	24.6
C57BL/6J	5806	B6.129P2-Gpr37<tm1Dgen>/J	45	1417	67.9	33	36.4
C57BL/6J	5807	B6.129P2-Gpr6<tm1Dgen>/J	39	1360	86.0	45	26.7
C57BL/6J	5813	B6.129P2-Grik5<tm1Dgen>/J	56	1501	47.8	32	43.8
C57BL/6J	5808	B6.129P2-Grk5<tm1Dgen>/J	55	1269	70.5	226	24.3
C57BL/6J	5814	B6.129P2-Grm1<tm1Dgen>/J	82	2173	47.9	81	33.3
C57BL/6J	5815	B6.129P2-Grm3<tm1Dgen>/J	55	1997	55.8	64	46.9
C57BL/6J	5817	B6.129P2-Gsk3b<tm1Dgen>/J	56	1686	62.9	264	51.8
C57BL/6J	5818	B6.129P2-Hcrtr1<tm1Dgen>/J	62	2028	46.8	48	33.3
C57BL/6J	5767	B6.129P2-Htr4<tm1Dgen>/J	97	2820	54.8	94	19.9
C57BL/6J	5769	B6.129P2-Htr7<tm1Dgen>/J	92	2237	48.0	72	16.7
C57BL/6J	4595	B6.129P2-Htt<tm2Detl>/J	50	1085	67.3	.	.
C57BL/6J	5820	B6.129P2-Il8ra<tm1Dgen>/J	70	1782	52.7	44	31.8
C57BL/6J	5830	B6.129P2-Kcnq2<tm1Dgen>/J	66	1392	51.8	94	42.3
C57BL/6J	5821	B6.129P2-Lats2<tm1Dgen>/J	55	1443	46.2	100	24.4

C57BL/6J	5822	B6.129P2-Lmbr1<tm1Dgen>/J	45	1237	54.5	27	48.1
C57BL/6J	5823	B6.129P2-Lrp5<tm1Dgen>/J	60	2349	58.4	172	16.7
C57BL/6J	5850	B6.129P2-Mapkapk2<tm1Dgen>/J	65	1992	36.9	58	27.6
C57BL/6J	5824	B6.129P2-Mmp17<tm1Dgen>/J	40	1319	49.7	30	40.0
C57BL/6J	5825	B6.129P2-Mtmr1<tm1Dgen>/J	40	1262	64.2	39	25.6
C57BL/6J	5778	B6.129P2-Naip1<tm1Dgen>/J	43	1192	58.3	24	45.8
C57BL/6J	5826	B6.129P2-Ntsr1<tm1Dgen>/J	65	2345	46.3	66	24.2
C57BL/6J	5829	B6.129P2-Pkd2l2<tm1Dgen>/J	80	2755	46.0	52	19.2
C57BL/6J	2829	B6.129P2-Plaur<tm1Jld>/J	40	1521	70.8	68	31.8
C57BL/6J	5828	B6.129P2-Ppard<tm1Dgen>/J	45	1280	51.7	30	50.0
C57BL/6J	5831	B6.129P2-Ppm1f<tm1Dgen>/J	60	1805	60.9	42	38.1
C57BL/6J	5827	B6.129P2-Ptch2<tm1Dgen>/J	40	1345	63.9	40	25.0
C57BL/6J	5832	B6.129P2-Ptpro<tm1Dgen>/J	40	1607	60.2	33	18.2
C57BL/6J	5837	B6.129P2-Scn11a<tm1Dgen>/J	111	3764	42.1	171	21.3
C57BL/6J	5836	B6.129P2-Scn9a<tm1Dgen>/J	66	1950	38.7	48	33.3
C57BL/6J	6620	B6.129P2-Scp2<tm1Usee>/J	10	360	38.3	.	.
C57BL/6J	5834	B6.129P2-Sema5a<tm1Dgen>/J	65	2615	50.0	58	27.6
C57BL/6J	5835	B6.129P2-Sema6c<tm1Dgen>/J	89	1913	63.5	144	16.7
C57BL/6J	6432	B6.129P2-Slc18a1<tm1Dgen>/J	30	875	64.7	22	72.7
C57BL/6J	5839	B6.129P2-Slc22a12<tm1Dgen>/J	70	1834	73.9	52	23.1
C57BL/6J	5838	B6.129P2-Slc22a6<tm1Dgen>/J	130	3792	38.8	104	23.1
C57BL/6J	5840	B6.129P2-Slc40a1<tm1Dgen>/J	42	1339	58.1	32	34.4
C57BL/6J	5841	B6.129P2-Slc6a9<tm1Dgen>/J	40	1372	72.8	73	8.3
C57BL/6J	5842	B6.129P2-Slc7a8<tm1Dgen>/J	50	1524	59.3	34	47.1
C57BL/6J	5843	B6.129P2-Slc9a6<tm1Dgen>/J	160	4362	12.1	128	40.6
C57BL/6J	5844	B6.129P2-Sstr1<tm1Dgen>/J	70	2237	48.6	34	41.2
C57BL/6J	6184	B6.129P2-Tbxas1<tm1Swl>/J	23	828	58.5	60	50.0
C57BL/6J	5847	B6.129P2-Tgfbr1<tm1Dgen>/J	89	2328	69.2	87	13.8
C57BL/6J	5845	B6.129P2-Thbs4<tm1Dgen>/J	47	1567	33.0	21	47.6
C57BL/6J	6434	B6.129P2-Tmprss13<tm1Dgen>/J	83	2924	55.9	68	35.3
C57BL/6J	5790	B6.129P2-Tpp1<tm1Dgen>/J	45	1408	58.7	22	50.0
C57BL/6J	5848	B6.129P2-Trpm5<tm1Dgen>/J	70	1808	62.1	82	39.0
C57BL/6J	4751	B6.129P2-Ugt8a<tm1Pop>/J	30	1083	53.1	.	.
C57BL/6J	5791	B6.129P2-Xcr1<tm1Dgen>/J	49	1216	61.8	104	30.8
C57BL/6J	6607	B6.129-Pctp<tm1Bor>/J	10	230	85.2	.	.
C57BL/6J	5702	B6.129-Pik3c2b<tm1Pkha>/J	40	1386	68.8	.	.
C57BL/6J	4584	B6.129-Pparg<tm2Rev>/J	64	2221	22.4	.	.
C57BL/6J	7605	B6.129P-Psen1<tm1Vln>/J	8	248	51.6	.	.
C57BL/6J	5617	B6.129P-Psen2<tm1Bdes>/J	30	1002	75.0	.	.
C57BL/6J	2984	B6.129S1-Il12rb1<tm1Jm>/J	8	259	33.2	.	.
C57BL/6J	7263	B6.129S1-Ing1<tm1Avg>/J	8	154	39.6	.	.
C57BL/6J	6221	B6.129S1-Lyve1<tm1Lhua>/J	10	390	53.6	.	.
C57BL/6J	6600	B6.129S1-Mnx1<tm4(cre)Tmj>/J	10	381	63.0	.	.
C57BL/6J	5846	B6.129S1-Tlr2<tm1Dgen>/J	60	1604	71.9	32	40.6
C57BL/6J	6848	B6.129S2(C)-Il8rb<tm1Mwm>/J	6	169	81.1	.	.
C57BL/6J	2612	B6.129S2-Bmp4<tm1Blh>/J	13	436	87.2	356	20.1
C57BL/6J	3114	B6.129S2-Crh<tm1Maj>/J	59	1009	64.4	74	33.9
C57BL/6J	3584	B6.129S2-H2<dlAb1-Ea>/J	60	2367	58.4	.	.
C57BL/6J	4513	B6.129S2-H2-DMa<tm1Doi>/J	40	1328	76.7	.	.
C57BL/6J	2762	B6.129S2-Irf1<tm1Mak>/J	20	658	82.1	.	.
C57BL/6J	2817	B6.129S2-Lck<tm1Mak>/J	43	1601	52.5	154	28.0
C57BL/6J	6187	B6.129S2-Nr4a1<tm1Jmi>/J	30	993	69.6	40	20.0
C57BL/6J	2509	B6.129S2-Plau<tm1Mlg>/J	40	796	52.9	23	56.5
C57BL/6J	5301	B6.129S2-Tg(APP)8.9Btla/J	40	1535	67.6	.	.
C57BL/6J	6490	B6.129S4-Abcb7<tm1Mdf>/J	10	305	57.4	.	.
C57BL/6J	2266	B6.129S4-Bdnf<tm1Jae>/J	35	1293	40.3	37	32.4
C57BL/6J	5517	B6.129S4-Cyb5r4<tm1Hfb>/HfbJ	39	1440	45.1	.	.
C57BL/6J	2198	B6.129S4-Dnmt1<tm1Jae>/J	49	1174	72.1	138	27.2
C57BL/6J	2958	B6.129S4-Drd3<tm1Dac>/J	39	1095	67.5	114	22.9
C57BL/6J	4067	B6.129S4-Ep300<tm1Dli>/J	50	1732	44.2	.	.
C57BL/6J	2952	B6.129S4-Il2ra<tm1Dw>/J	80	2938	40.9	184	15.1
C57BL/6J	3991	B6.129S4-Itgam<tm1Myd>/J	20	726	28.4	.	.
C57BL/6J	6503	B6.129S4-Lpl<tm1Ijg>/J	10	194	38.7	.	.
C57BL/6J	3515	B6.129S4-Lyn<tm1Sor>/J	20	626	80.2	123	24.1
C57BL/6J	2275	B6.129S4-Ntf3<tm1Jae>/J	39	1146	69.8	78	17.9
C57BL/6J	6582	B6.129S4-Park2<tm1Shn>/J	6	92	81.5	.	.
C57BL/6J	7669	B6.129S4-Pdgfra<tm11(EGFP)Sor>/J	8	196	55.1	.	.
C57BL/6J	5901	B6.129S4-Ppard<tm2Rev>/J	20	752	86.7	33	33.3
C57BL/6J	6142	B6.129S4-Pparg<tm1Rev>/J	29	964	64.3	58	44.8
C57BL/6J	4189	B6.129S4-Prkce<tm1Msg>/J	31	1225	64.1	.	.
C57BL/6J	7609	B6.129S4-Strap<Gt(ROSA)71Sor>/J	8	357	62.2	.	.
C57BL/6J	6406	B6.129S4-Tg(APPSwLon)96Btla/J	5	178	53.4	.	.
C57BL/6J	6469	B6.129S4-Tg(PSEN1H163R)G9Btla/J	6	125	82.4	.	.
C57BL/6J	6133	B6.129S4-Vdr<tm1Mbd>/J	42	1284	49.4	80	35.0
C57BL/6J	2719	B6.129S4-Wt1<tm1Jae>/J	15	524	56.7	.	.
C57BL/6J	5862	B6.129S6-Esam1<tm1Tq>/J	41	1057	58.4	28	32.1
C57BL/6J	7621	B6.129S6-Hr<tm1Cct>/J	8	211	31.8	.	.
C57BL/6J	6878	B6.129S6-Tagln<tm2(cre)Yec>/J	8	295	75.6	.	.
C57BL/6J	5970	B6.129S7-Atoh1<tm2Hzo>/J	30	964	66.3	59	30.5
C57BL/6J	3336	B6.129S7-Cdkn1c<tm1Sje>/J	40	1348	51.6	794	25.7
C57BL/6J	4164	B6.129S7-Chrna3<tm1Bay>/J	10	251	37.8	.	.
C57BL/6J	6039	B6.129S7-Efnb2<tm1And>/J	10	266	9.8	.	.
C57BL/6J	6042	B6.129S7-Efnb2<tm2And>/J	10	232	5.2	.	.
C57BL/6J	6201	B6.129-Scd1<tm1Ntam>/J	10	262	9.2	.	.
C57BL/6J	6879	B6.129-Scd2<tm1Myz>/J	8	137	70.8	.	.
C57BL/6J	6156	B6.129-Scel<tm1Hba>/J	20	808	79.0	43	27.9
C57BL/6J	6336	B6.129-Selplg<tm1Rpmc>/J	6	153	74.5	.	.
C57BL/6J	6301	B6.129S-Id3<tm1Zhu>/J	10	378	28.8	.	.
C57BL/6J	5709	B6.129-Ski<tm1Cco>/J	11	466	44.6	.	.
C57BL/6J	6497	B6.129-Skil<tm2Spw>/J	10	288	53.1	.	.
C57BL/6J	5960	B6.129S-Pecam1<Gt(VICTR20)12Lex>/J	20	652	78.1	56	28.6
C57BL/6J	5669	B6.129S-Runx1<tm1Spe>/J	53	1828	68.6	.	.
C57BL/6J	5623	B6.129S-Shh<tm2(cre/ESR1)Cjt>/J	10	406	74.9	.	.
C57BL/6J	4650	B6.129-Tlr2<tm1Kir>/J	9	366	29.8	.	.
C57BL/6J	5434	B6.129-Tmhs<tm1Kjn>/Kjn	20	742	59.4	.	.
C57BL/6J	5849	B6.129-Tmprss11a<tm1Dgen>/J	58	1505	39.5	24	45.8
C57BL/6J	5039	B6.129X1-Adra1a<tm1Pcs>/J	30	959	76.4	.	.
C57BL/6J	6199	B6.129X1-Fzd9<tm1Uta>/J	10	458	83.6	.	.
C57BL/6J	5248	B6.129X1-Igfbp1<tm1Taub>/J	30	977	70.4	.	.
C57BL/6J	6072	B6.129X1-Mcl1<tm2Sjk>/J	20	599	37.6	60	15.0
C57BL/6J	4265	B6.129X1-Mpo<tm1Lus>/J	37	1104	69.1	.	.
C57BL/6J	5643	B6.129X-Gusb<tm1Sly>/J	25	1007	60.1	.	.
C57BL/6J	797	B6.CAST-Gpi1<a>/EiJ	21	542	55.7	42	24.4

C57BL/6J	3904	B6.CBA-Tg(CETP)5203Tall/J	70	2550	69.9	72	36.1
C57BL/6J	6952	B6.Cg-Akt2<tm1.1Mbb> Ldlr<tm1Her>/J	8	230	4.8	.	.
C57BL/6J	6253	B6.Cg-Ap3b1<tm1.1Sms>/J	10	329	12.5	.	.
C57BL/6J	6230	B6.Cg-Cebpa<tm1Dgt> Tg(Mx1-cre)1Cgn/J	10	284	70.4	.	.
C57BL/6J	6183	B6.Cg-Col4a5<tm1Yseg>/J	37	1011	56.0	98	42.9
C57BL/6J	3826	B6.Cg-cub/J	10	443	49.0	23	30.4
C57BL/6J	6772	B6.Cg-Foxp3<tm2Tch>/J	6	130	86.2	.	.
C57BL/6J	6407	B6.Cg-Gusb<mps>/BrkJ	20	674	26.6	.	.
C57BL/6J	6908	B6.Cg-Ikbke<tm1Tman>/J	6	143	69.2	.	.
C57BL/6J	6580	B6.Cg-Ins2<Akita> Ldlr<tm1Her>/J	10	150	23.3	.	.
C57BL/6J	5051	B6.Cg-Kit<W-sh>/HNihrJaeBsmJ	35	1014	71.5	.	.
C57BL/6J	6883	B6.Cg-Ldlr<tm1Her> Sod2<tm1Leb>/J	10	231	61.9	.	.
C57BL/6J	6877	B6.Cg-Ldlr<tm1Her> Tg(H2-K-AKR1B1)1Tj/J	6	176	43.2	.	.
C57BL/6J	6906	B6.Cg-Lep<ob> Ldlr<tm1Her>/J	6	193	20.2	.	.
C57BL/6J	6865	B6.Cg-Mag<tm1Rod>/J	8	238	45.4	.	.
C57BL/6J	5491	B6.Cg-Mapt<tm1(EGFP)Klt> Tg(MAPT)8cPdav/J	15	246	87.0	.	.
C57BL/6J	6124	B6.Cg-Myo6<sv-2J>/J	31	883	40.2	18	33.3
C57BL/6J	6097	B6.Cg-Nfkb1<tm1Bal>/J	15	453	71.7	.	.
C57BL/6J	6577	B6.Cg-Park7<tm1Shn>/J	6	126	92.9	.	.
C57BL/6J	6194	B6.Cg-Polq<tm1Jcs>/J	8	232	49.6	.	.
C57BL/6J	6922	B6.Cg-Sfpi1<tm2Dgt>/J	8	225	85.3	.	.
C57BL/6J	3780	B6.Cg-Sgsh<mps3a>/PstJ	40	1516	50.9	37	24.3
C57BL/6J	5622	B6.Cg-Shh<tm1(EGFP/cre)Cjt>/J	9	316	74.4	.	.
C57BL/6J	6612	B6.Cg-Tg(ACTA1-MYOT)12Mah/J	6	215	77.2	.	.
C57BL/6J	6615	B6.Cg-Tg(ACTA1-MYOT*T57I)71Mah/J	6	117	66.7	.	.
C57BL/6J	6055	B6.Cg-Tg(ACTB-Bgeo,-DsRed*MST)1Nagy/J	23	606	81.5	.	.
C57BL/6J	4178	B6.Cg-Tg(ACTB-Bgeo/GFP)21Lbe/J	25	762	81.2	.	.
C57BL/6J	5866	B6.Cg-Tg(APP695)3Dbo Tg(PSEN1dE9)S9Dbo/J	154	4687	18.1	300	15.2
C57BL/6J	5864	B6.Cg-Tg(APPswe,PSEN1dE9)85Dbo/J	69	1995	60.3	200	28.0
C57BL/6J	5317	B6.Cg-Tg(BAT-lacZ)3Picc/J	12	422	53.6	.	.
C57BL/6J	2320	B6.Cg-Tg(BCL2)25Wehi/J	22	667	65.5	170	25.1
C57BL/6J	5359	B6.Cg-Tg(Camk2a-cre)T29-1Stl/J	20	722	60.0	.	.
C57BL/6J	5855	B6.Cg-Tg(Camk2a-Prkaca)426Tabe/J	43	1381	58.6	218	19.7
C57BL/6J	7004	B6.Cg-Tg(Camk2a-tTA)1Mmay/DboJ	8	321	68.5	.	.
C57BL/6J	5551	B6.Cg-Tg(Cd4-TGFBR2)16Flv/J	30	826	88.4	.	.
C57BL/6J	6137	B6.Cg-Tg(Cdh5-cre)7Mlia/J	20	459	23.5	.	.
C57BL/6J	6368	B6.Cg-Tg(Cr2-cre)3Cgn/J	10	244	16.4	.	.
C57BL/6J	6229	B6.Cg-Tg(DRE-lacZ)2Gswz/J	10	348	52.9	.	.
C57BL/6J	5000	B6.Cg-Tg(F2RL1)1Mslb/J	30	970	26.3	.	.
C57BL/6J	4631	B6.Cg-Tg(GFAP-APOE*4)1Hol Apoe<tm1Unc>/J	15	509	40.7	.	.
C57BL/6J	5964	B6.Cg-Tg(GFAP-tTA)110Pop/J	20	588	77.0	59	33.9
C57BL/6J	4753	B6.Cg-Tg(Il1rn)1Dih/J	30	486	87.2	.	.
C57BL/6J	6098	B6.Cg-Tg(Il2/NFAT-luc)83Rinc/J	10	290	73.8	.	.
C57BL/6J	3802	B6.Cg-Tg(Lck-cre)548Jxm/J	20	645	38.9	.	.
C57BL/6J	6293	B6.Cg-Tg(PDGFB-APPSwInd)20Lms/2J	40	1306	65.8	.	.
C57BL/6J	5975	B6.Cg-Tg(Plp1-cre/ESR1)3.16Pop/J	30	1192	56.5	116	31.0
C57BL/6J	6006	B6.Cg-Tg(Prnp-APP)A-2Dbo/J	40	1168	60.4	120	16.7
C57BL/6J	6005	B6.Cg-Tg(Prnp-App/APPswe)E1-2Dbo/J	51	1465	4.6	.	.
C57BL/6J	3967	B6.Cg-Tg(Rbp3-cre)528Jxm/J	20	789	41.3	122	32.6
C57BL/6J	6438	B6.Cg-Tg(Scgb1a1-Scnn1b)6608Bouc/J	10	222	53.2	.	.
C57BL/6J	4435	B6.Cg-Tg(SOD1*G93A)1Gur/J	983	29900	37.5	.	.
C57BL/6J	6361	B6.Cg-Tg(Sp7-tTA,tetO-EGFP/cre)1Amc/J	99	2933	30.8	.	.
C57BL/6J	3966	B6.Cg-Tg(Syn1-cre)671Jxm/J	70	1972	80.4	400	18.5
C57BL/6J	4128	B6.Cg-Tg(Tek-cre)12Flv/J	31	653	43.8	.	.
C57BL/6J	7051	B6.Cg-Tg(tetO-APPSwInd)102Dbo/J	8	296	73.3	.	.
C57BL/6J	7052	B6.Cg-Tg(tetO-APPSwInd)107Dbo/J	10	380	50.8	.	.
C57BL/6J	7049	B6.Cg-Tg(tetO-APPSwInd)885Dbo/J	8	260	58.8	.	.
C57BL/6J	7612	B6.Cg-Tg(Thy1-COP4/EYFP)18Gfng/J	12	350	86.0	.	.
C57BL/6J	7615	B6.Cg-Tg(Thy1-COP4/EYFP)9Gfng/J	12	308	81.5	.	.
C57BL/6J	7606	B6.Cg-Tg(Thy1-cre/ESR1,-EYFP)AGfng/J	12	380	75.0	.	.
C57BL/6J	7610	B6.Cg-Tg(Thy1-cre/ESR1,-EYFP)VGfng/J	8	357	80.4	.	.
C57BL/6J	4659	B6.Cg-Tg(TIE2GFP)287Sato/1J	101	2280	63.5	.	.
C57BL/6J	6101	B6.Cg-Tg(TRE/Prl-luc)31FlvRinc/J	30	993	74.2	52	30.8
C57BL/6J	6475	B6.FVB(129S4)-Tg(Ckmm-cre)5Khn/J	8	251	73.7	.	.
C57BL/6J	4971	B6.FVB-Tg(CD46)2Gsv/J	31	1085	5.0	.	.
C57BL/6J	6576	B6.FVB-Tg(GNAT2-Dta)98Wwk/J	10	342	20.2	.	.
C57BL/6J	6000	B6.FVB-Tg(ITGAM-DTR/EGFP)34Lan/J	41	709	78.6	40	30.0
C57BL/6J	4509	B6.FVB-Tg(Itgax-DTR/EGFP)57Lan/J	45	1639	40.3	.	.
C57BL/6J	6417	B6.FVB-Tg(Npy-hrGFP)1Lowl/J	10	262	31.7	.	.
C57BL/6J	5738	B6.FVB-Tg(tetO-EGFP,-Tgfbr2)8Mcle/J	10	193	88.6	.	.
C57BL/6J	3951	B6.P2-P2rx3<tm1Ckn>/J	40	1651	35.8	236	29.7
C57BL/6J	6086	B6.SJL-Tg(HBB-GH1)420King/J	128	3808	38.3	208	32.7
C57BL/6J	632	B6.V-Lep<ob>/J	30	1129	73.4	.	.
C57BL/6J	6329	B6;129-Bax<tm2Sjk> Bak1<tm1Thsn>/J	10	312	64.1	.	.
C57BL/6J	6911	B6;129-Gt(ROSA)26Sor<tm1(rtTA*M2)Jae> Col1a1<tm2(tetO-Pou5f1)Jae>/J	8	182	37.4	.	.
C57BL/6J	6011	B6;129-Ly9<tm1Mckn>/J	10	280	36.1	.	.
C57BL/6J	6904	B6;129-Msc<tm1Eno>/J	8	256	68.4	.	.
C57BL/6J	6377	B6;129-Nrxn3<tm1Sud> Nrxn1<tm1Sud> Nrxn2<tm1Sud>/J	8	166	69.3	.	.
C57BL/6J	5788	B6;129P2-Cd97<tm1Dgen>/J	40	1454	79.5	30	26.7
C57BL/6J	5833	B6;129P2-Rgs4<tm1Dgen>/J	52	1468	57.4	31	35.5
C57BL/6J	6568	B6;129P2-Terf2<tm1Tdl>/J	8	215	75.3	.	.
C57BL/6J	6375	B6;129-Rab3b<tm1Sud> Rab3a<tm1Sud> Rab3d<tm1Rja> Rab3c<tm1Sud>/J	10	316	44.3	.	.
C57BL/6J	6258	B6;129S4-Apoa2<tm1Bres>/J	8	291	75.3	.	.
C57BL/6J	6404	B6;129S4-Apoa4<tm1Bres>/J	8	209	30.6	.	.
C57BL/6J	7200	B6;129S4-Arid5b<Gt(ROSA)75Sor>/J	8	294	44.9	.	.
C57BL/6J	7671	B6;129S4-Fgfr1<tm5Sor>/J	5	175	79.4	.	.
C57BL/6J	7670	B6;129S4-Gt(ROSA)26Sor<tm3(phiC31*)Sor>/J	9	222	66.7	.	.
C57BL/6J	6414	B6;129S4-Mc4r<tm1Lowl>/J	10	289	74.4	.	.
C57BL/6J	7201	B6;129S4-Plekha1<Gt(ROSA)82Sor>/J	8	261	54.8	.	.
C57BL/6J	7203	B6;129S4-Zfand5<Gt(ROSA)72Sor>/J	8	213	69.0	.	.
C57BL/6J	6410	B6;129S6-Chat<tm1(cre)Lowl>/J	12	191	40.8	.	.
C57BL/6J	6208	B6;129S6-Pdzk1<tm1Dls>/J	10	308	62.0	.	.
C57BL/6J	6044	B6;129S7-Ephb4<tm1And>/J	10	311	32.2	.	.
C57BL/6J	6470	B6;129S-Hopx<tm1Eno>/J	8	274	63.5	.	.
C57BL/6J	6390	B6;129-Snca<tm1Sud> Sncb<tm1.1Sud>/J	10	300	71.3	.	.
C57BL/6J	6958	B6;129S-Nkd1<tm1Kwha>/J	8	358	27.7	.	.
C57BL/6J	6251	B6;129-Tor1a<tm1Wtd>/J	11	352	61.4	.	.
C57BL/6J	6980	B6;129-Trp53<tm2Xu>/J	8	280	48.2	.	.
C57BL/6J	6614	B6;CB-Tg(Thy1-CFP/COX8A)C1Lich/J	15	660	75.6	.	.
C57BL/6J	6617	B6;CB-Tg(Thy1-CFP/COX8A)S2Lich/J	37	909	65.6	.	.
C57BL/6J	6033	B6;CByJ-nmf445/J	20	664	72.7	24	29.2
C57BL/6J	6302	B6;SJL-Slc6a3<tm1.1(cre)Bkmn>/J	8	249	88.8	.	.
C57BL/6J	7622	B6;SJL-Tg(KRT14-Hr)551Cct/J	8	313	19.5	.	.

C57BL/6J	6043	B6;SJL-Tg(Oxt/EGFP)AI03Wsy/J	10	272	76.5	.	.
C57BL/6J	6575	C57BL/6-Camk2a<tm1Vyb>/J	10	339	69.6	.	.
C57BL/6J	6468	C57BL/6-Chrm1<tm1Stl>/J	10	303	50.5	.	.
C57BL/6J	2518	C57BL/6-Il4<tm1Nnt>/J	15	509	61.5	.	.
C57BL/6J	3548	C57BL/6-Ins2<Akita>/J	138	4154	42.6	220	30.7
C57BL/6J	664	C57BL/6J	520	15725	59.9	1020	43.1
C57BL/6J	2020	C57BL/6J-Apc<Min>/J	132	3682	46.9	.	.
C57BL/6J	5921	C57BL/6J-Aqp2<F204V>/J	30	892	71.3	58	34.5
C57BL/6J	5762	C57BL/6J-Chr X.1<PWD/Ph>/ForeJ	30	948	18.4	48	37.5
C57BL/6J	5253	C57BL/6J-Clcn1<adr-mto6J>/J	20	553	75.2	.	.
C57BL/6J	3129	C57BL/6J-Epha4<rb-2J>/J	20	772	38.1	25	48.0
C57BL/6J	5013	C57BL/6J-Gars<Nmf249>/J	21	696	42.8	.	.
C57BL/6J	5561	C57BL/6J-Grm1<nmf373>/J	40	1402	23.0	.	.
C57BL/6J	2009	C57BL/6J-Hook1<azh>/J	15	424	0.5	.	.
C57BL/6J	5748	C57BL/6J-Kcnq1<vtg-3J>/J	16	621	45.9	49	24.7
C57BL/6J	5635	C57BL/6J-Lama2<dy-7J>/J	40	1151	79.1	.	.
C57BL/6J	5761	C57BL/6J-mt<PWD/Ph>/ForeJ	30	984	39.3	72	22.2
C57BL/6J	5749	C57BL/6J-Myo6<sv-3J>/J	81	2629	7.4	66	4.5
C57BL/6J	2561	C57BL/6J-Nek8<jck>/J	150	4424	46.4	.	.
C57BL/6J	5101	C57BL/6J-nmf268/J	25	887	22.7	.	.
C57BL/6J	5448	C57BL/6J-nmf347/J	32	1215	34.3	.	.
C57BL/6J	5741	C57BL/6J-nmf356/J	30	786	68.1	.	.
C57BL/6J	5467	C57BL/6J-nmf370/J	33	1072	50.7	.	.
C57BL/6J	5451	C57BL/6J-nmf391/J	30	784	71.9	.	.
C57BL/6J	4817	C57BL/6J-Npc1<nmf164>/J	72	2304	75.1	.	.
C57BL/6J	6699	C57BL/6J-Pcsk1<N222D>/J	8	202	76.7	.	.
C57BL/6J	810	C57BL/6J-Ptpn6<me>/J	6	79	93.7	.	.
C57BL/6J	5744	C57BL/6J-Reln<rl-6J>/J	80	2485	22.1	34	38.2
C57BL/6J	5482	C57BL/6J-Shar/J	30	929	75.8	.	.
C57BL/6J	219	C57BL/6J-Slc30a4<lm>/J	34	900	61.9	22	27.3
C57BL/6J	5449	C57BL/6J-Spnb4<qv-8J>/J	18	567	38.4	.	.
C57BL/6J	5863	C57BL/6J-Tg(ACTB-DDAH1)1Jpck/J	31	1278	61.3	56	30.4
C57BL/6J	6481	C57BL/6J-Tg(ACTB-NOTCH1)1Shn/J	20	595	63.5	.	.
C57BL/6J	5967	C57BL/6J-Tg(Mt1-Tnfsf4)1Pgn/Pgn	30	978	46.1	34	47.1
C57BL/6J	5968	C57BL/6J-Tg(Mt1-Tnfsf4)2Pgn/Pgn	20	618	77.8	40	25.0
C57BL/6J	2355	C57BL/6J-Tg(pPWL88puro)2Ems/J	92	3035	45.1	236	17.9
C57BL/6J	5037	C57BL/6J-Ticam1<Lps2>/J	25	856	79.1	.	.
C57BL/6J	5638	C57BL/6J-Tmhs<hscy-2J>/J	30	517	57.4	.	.
C57BL/6J	6581	C57BL/6-Ppp3r1<tm1Stl>/J	6	95	81.1	.	.
C57BL/6J	6662	C57BL/6-Tg(ACTB-MAP2K1*K97M)1Stl/J	9	216	37.0	.	.
C57BL/6J	5145	C57BL/6-Tg(ACTB-OVA)916Jen/J	25	944	14.2	.	.
C57BL/6J	6579	C57BL/6-Tg(Camk2a-Bdnf)A9Stl/J	18	567	64.6	.	.
C57BL/6J	6474	C57BL/6-Tg(Grik4-cre)G32-4Stl/J	6	157	72.0	.	.
C57BL/6J	5432	C57BL/6-Tg(Ins2-OVA)307Wehi/WehiJ	20	737	24.4	.	.
C57BL/6J	5433	C57BL/6-Tg(Ins2-OVA)59Wehi/WehiJ	40	1505	49.6	.	.
C57BL/6J	5431	C57BL/6-Tg(Ins2-TFRC/OVA)296Wehi/WehiJ	80	2736	26.9	.	.
C57BL/6J	2598	C57BL/6-Tg(KLK4mHEL)6Ccg/J	44	1424	61.4	78	15.4
C57BL/6J	6912	C57BL/6-Tg(Tcra2D2,Tcrb2D2)1Kuch/J	8	242	56.6	.	.
C57BL/6J	6140	C57BL/6-Tg(TERT)C10Hode/J	10	265	70.2	.	.
C57BL/6J	5706	C57BL/6-Tg(tetO-CDK5R1/GFP)337Lht/J	26	798	82.9	.	.
C57BL/6J	6618	C57BL/6-Tg(tetO-COX8A/EYFP)1Ksn/J	6	194	80.9	.	.
C57BL/6J	3135	C57BL/6-Tg(TRAMP)8247Ng/J	194	6239	43.3	1673	26.6
C57BL/6J	C57BL/6J-108	Private Strain	6	227	81.5	.	.
C57BL/6J	C57BL/6J-184	Private Strain	22	606	58.9	.	.
C57BL/6J	C57BL/6J-240	Private Strain	440	8921	43.3	.	.
C57BL/6J	C57BL/6J-257	Private Strain	20	331	84.9	.	.
C57BL/6J	C57BL/6J-27	Private Strain	32	1255	54.8	.	.
C57BL/6J	C57BL/6J-285	Private Strain	11	418	80.1	11	27.3
C57BL/6J	C57BL/6J-286	Private Strain	10	129	82.2	.	.
C57BL/6J	C57BL/6J-287	Private Strain	10	414	51.2	.	.
C57BL/6J	C57BL/6J-288	Private Strain	95	1484	54.1	.	.
C57BL/6J	C57BL/6J-289	Private Strain	35	1051	4.8	.	.
C57BL/6J	C57BL/6J-290	Private Strain	24	745	71.3	.	.
C57BL/6J	C57BL/6J-291	Private Strain	15	638	36.2	.	.
C57BL/6J	C57BL/6J-292	Private Strain	35	1335	19.6	.	.
C57BL/6J	C57BL/6J-293	Private Strain	40	1574	76.2	.	.
C57BL/6J	C57BL/6J-294	Private Strain	30	1004	53.2	24	20.8
C57BL/6J	C57BL/6J-295	Private Strain	40	1240	53.7	102	21.2
C57BL/6J	C57BL/6J-296	Private Strain	40	1406	23.6	25	40.0
C57BL/6J	C57BL/6J-297	Private Strain	15	555	76.0	150	45.9
C57BL/6J	C57BL/6J-298	Private Strain	9	323	87.0	40	35.0
C57BL/6J	C57BL/6J-299	Private Strain	10	462	57.1	.	.
C57BL/6J	C57BL/6J-30	Private Strain	12	241	21.2	.	.
C57BL/6J	C57BL/6J-300	Private Strain	10	155	83.9	.	.
C57BL/6J	C57BL/6J-301	Private Strain	130	3406	23.1	144	47.9
C57BL/6J	C57BL/6J-302	Private Strain	20	500	2.0	.	.
C57BL/6J	C57BL/6J-303	Private Strain	20	736	17.7	.	.
C57BL/6J	C57BL/6J-304	Private Strain	40	1588	62.9	40	35.0
C57BL/6J	C57BL/6J-305	Private Strain	40	1114	41.2	24	29.2
C57BL/6J	C57BL/6J-306	Private Strain	20	671	74.8	40	32.5
C57BL/6J	C57BL/6J-307	Private Strain	10	434	79.5	.	.
C57BL/6J	C57BL/6J-308	Private Strain	8	325	60.3	.	.
C57BL/6J	C57BL/6J-309	Private Strain	8	379	42.2	.	.
C57BL/6J	C57BL/6J-31	Private Strain	10	358	77.4	.	.
C57BL/6J	C57BL/6J-310	Private Strain	8	197	43.7	.	.
C57BL/6J	C57BL/6J-311	Private Strain	9	259	64.9	.	.
C57BL/6J	C57BL/6J-312	Private Strain	9	381	38.1	.	.
C57BL/6J	C57BL/6J-313	Private Strain	10	330	55.5	.	.
C57BL/6J	C57BL/6J-314	Private Strain	10	325	79.1	.	.
C57BL/6J	C57BL/6J-315	Private Strain	15	430	83.7	.	.
C57BL/6J	C57BL/6J-316	Private Strain	15	603	53.7	.	.
C57BL/6J	C57BL/6J-317	Private Strain	10	231	75.8	.	.
C57BL/6J	C57BL/6J-318	Private Strain	23	648	54.7	.	.
C57BL/6J	C57BL/6J-319	Private Strain	10	247	56.7	.	.
C57BL/6J	C57BL/6J-320	Private Strain	10	258	74.0	.	.
C57BL/6J	C57BL/6J-321	Private Strain	20	742	31.0	.	.
C57BL/6J	C57BL/6J-322	Private Strain	10	450	68.9	.	.
C57BL/6J	C57BL/6J-323	Private Strain	10	339	63.4	.	.
C57BL/6J	C57BL/6J-324	Private Strain	11	326	4.0	.	.
C57BL/6J	C57BL/6J-325	Private Strain	10	278	67.6	.	.
C57BL/6J	C57BL/6J-326	Private Strain	6	261	19.9	.	.
C57BL/6J	C57BL/6J-327	Private Strain	10	366	91.0	.	.
C57BL/6J	C57BL/6J-328	Private Strain	10	366	80.6	.	.

C57BL/6J	C57BL/6J-329	Private Strain	15	660	75.6	.	.
C57BL/6J	C57BL/6J-330	Private Strain	37	909	65.6	.	.
C57BL/6J	C57BL/6J-331	Private Strain	10	123	61.0	.	.
C57BL/6J	C57BL/6J-332	Private Strain	10	418	34.2	.	.
C57BL/6J	C57BL/6J-333	Private Strain	20	524	5.7	.	.
C57BL/6J	C57BL/6J-334	Private Strain	10	180	11.1	.	.
C57BL/6J	C57BL/6J-335	Private Strain	20	964	33.1	.	.
C57BL/6J	C57BL/6J-336	Private Strain	10	217	79.7	.	.
C57BL/6J	C57BL/6J-337	Private Strain	10	230	61.3	.	.
C57BL/6J	C57BL/6J-338	Private Strain	10	423	56.3	.	.
C57BL/6J	C57BL/6J-339	Private Strain	10	239	39.7	.	.
C57BL/6J	C57BL/6J-340	Private Strain	10	366	68.0	.	.
C57BL/6J	C57BL/6J-341	Private Strain	10	452	61.5	.	.
C57BL/6J	C57BL/6J-342	Private Strain	10	451	83.1	.	.
C57BL/6J	C57BL/6J-343	Private Strain	10	197	36.0	.	.
C57BL/6J	C57BL/6J-344	Private Strain	10	356	44.4	.	.
C57BL/6J	C57BL/6J-345	Private Strain	10	355	26.5	.	.
C57BL/6J	C57BL/6J-346	Private Strain	10	327	68.5	.	.
C57BL/6J	C57BL/6J-347	Private Strain	11	343	70.8	.	.
C57BL/6J	C57BL/6J-348	Private Strain	30	1025	65.2	.	.
C57BL/6J	C57BL/6J-349	Private Strain	9	171	77.2	.	.
C57BL/6J	C57BL/6J-350	Private Strain	8	229	72.9	.	.
C57BL/6J	C57BL/6J-351	Private Strain	12	210	24.8	.	.
C57BL/6J	C57BL/6J-352	Private Strain	7	168	84.5	.	.
C57BL/6J	C57BL/6J-353	Private Strain	6	207	86.5	.	.
C57BL/6J	C57BL/6J-354	Private Strain	10	358	58.7	.	.
C57BL/6J	C57BL/6J-355	Private Strain	20	617	71.8	38	36.8
C57BL/6J	C57BL/6J-356	Private Strain	8	148	75.7	.	.
C57BL/6J	C57BL/6J-357	Private Strain	8	146	63.0	.	.
C57BL/6J	C57BL/6J-358	Private Strain	6	181	66.3	.	.
C57BL/6J	C57BL/6J-359	Private Strain	6	117	85.5	.	.
C57BL/6J	C57BL/6J-360	Private Strain	20	572	34.8	.	.
C57BL/6J	C57BL/6J-361	Private Strain	20	632	76.7	.	.
C57BL/6J	C57BL/6J-362	Private Strain	8	314	66.6	.	.
C57BL/6J	C57BL/6J-363	Private Strain	8	293	50.5	.	.
C57BL/6J	C57BL/6J-364	Private Strain	8	283	74.9	.	.
C57BL/6J	C57BL/6J-365	Private Strain	8	211	62.6	.	.
C57BL/6J	C57BL/6J-366	Private Strain	8	322	73.6	.	.
C57BL/6J	C57BL/6J-367	Private Strain	4	320	87.5	.	.
C57BL/6J	C57BL/6J-368	Private Strain	8	344	68.3	.	.
C57BL/6J	C57BL/6J-369	Private Strain	8	241	83.0	.	.
C57BL/6J	C57BL/6J-370	Private Strain	8	204	52.9	.	.
C57BL/6J	C57BL/6J-371	Private Strain	8	302	35.1	.	.
C57BL/6J	C57BL/6J-372	Private Strain		294	50.3	.	.
C57BL/6J	C57BL/6J-373	Private Strain		202	53.5	.	.
C57BL/6J	C57BL/6J-374	Private Strain		308	20.5	.	.
C57BL/6J	C57BL/6J-375	Private Strain		303	48.5	.	.
C57BL/6J	C57BL/6J-376	Private Strain		332	56.3	.	.
C57BL/6J	C57BL/6J-377	Private Strain		333	60.1	.	.
C57BL/6J	C57BL/6J-378	Private Strain	8	253	70.4	.	.
C57BL/6J	C57BL/6J-379	Private Strain	9	316	85.1	.	.
C57BL/6J	C57BL/6J-38	Private Strain	15	347	0.0	.	.
C57BL/6J	C57BL/6J-380	Private Strain	8	197	57.9	.	.
C57BL/6J	C57BL/6J-381	Private Strain	8	407	29.5	.	.
C57BL/6J	C57BL/6J-382	Private Strain	8	242	68.2	.	.
C57BL/6J	C57BL/6J-383	Private Strain	8	317	52.7	.	.
C57BL/6J	C57BL/6J-384	Private Strain	30	1027	7.2	.	.
C57BL/6J	C57BL/6J-385	Private Strain	24	749	25.9	.	.
C57BL/6J	C57BL/6J-386	Private Strain	5	155	37.4	.	.
C57BL/6J	C57BL/6J-387	Private Strain	50	1954	58.0	.	.
C57BL/6J	C57BL/6J-388	Private Strain	20	714	70.7	44	11.4
C57BL/6J	C57BL/6J-389	Private Strain	19	465	77.0	.	.
C57BL/6J	C57BL/6J-390	Private Strain	10	196	74.5	.	.
C57BL/6J	C57BL/6J-391	Private Strain	6	139	63.3	.	.
C57BL/6J	C57BL/6J-392	Private Strain	6	144	58.3	.	.
C57BL/6J	C57BL/6J-393	Private Strain	20	596	60.7	.	.
C57BL/6J	C57BL/6J-394	Private Strain	20	667	82.3	.	.
C57BL/6J	C57BL/6J-395	Private Strain	19	384	43.0	.	.
C57BL/6J	C57BL/6J-396	Private Strain	45	848	46.9	144	8.3
C57BL/6J	C57BL/6J-397	Private Strain	126	4084	57.4	.	.
C57BL/6J	C57BL/6J-398	Private Strain	41	1121	67.8	50	28.0
C57BL/6J	C57BL/6J-399	Private Strain	13	255	48.2	.	.
C57BL/6J	C57BL/6J-400	Private Strain	10	366	36.9	.	.
C57BL/6J	C57BL/6J-401	Private Strain	10	249	44.6	.	.
C57BL/6J	C57BL/6J-402	Private Strain	19	461	66.7	.	.
C57BL/6J	C57BL/6J-403	Private Strain	20	575	86.1	.	.
C57BL/6J	C57BL/6J-404	Private Strain	14	252	68.3	.	.
C57BL/6J	C57BL/6J-405	Private Strain	30	1240	70.8	.	.
C57BL/6J	C57BL/6J-406	Private Strain	30	1128	76.2	.	.
C57BL/6J	C57BL/6J-407	Private Strain	30	885	80.0	.	.
C57BL/6J	C57BL/6J-408	Private Strain	10	246	23.6	.	.
C57BL/6J	C57BL/6J-409	Private Strain	30	795	79.1	.	.
C57BL/6J	C57BL/6J-410	Private Strain	20	521	73.5	.	.
C57BL/6J	C57BL/6J-411	Private Strain	30	1264	69.6	.	.
C57BL/6J	C57BL/6J-412	Private Strain	23	785	53.1	32	6.3
C57BL/6J	C57BL/6J-413	Private Strain	40	1593	53.6	.	.
C57BL/6J	C57BL/6J-414	Private Strain	20	744	66.1	39	7.7
C57BL/6J	C57BL/6J-415	Private Strain	20	697	68.6	42	16.7
C57BL/6J	C57BL/6J-416	Private Strain	20	566	51.8	24	33.3
C57BL/6J	C57BL/6J-417	Private Strain	20	762	68.2	.	.
C57BL/6J	C57BL/6J-418	Private Strain	27	787	40.8	.	.
C57BL/6J	C57BL/6J-419	Private Strain	25	1010	53.1	48	50.0
C57BL/6J	C57BL/6J-420	Private Strain	30	898	68.9	32	31.3
C57BL/6J	C57BL/6J-421	Private Strain	101	3295	63.7	.	.
C57BL/6J	C57BL/6J-422	Private Strain	10	398	77.9	42	28.6
C57BL/6J	C57BL/6J-423	Private Strain	20	398	78.1	.	.
C57BL/6J	C57BL/6J-424	Private Strain	10	411	74.0	36	30.6
C57BL/6J	C57BL/6J-425	Private Strain	25	792	80.1	.	.
C57BL/6J	C57BL/6J-426	Private Strain	11	354	86.7	33	36.4
C57BL/6J	C57BL/6J-427	Private Strain	20	457	85.3	.	.
C57BL/6J	C57BL/6J-428	Private Strain	10	402	64.7	19	36.8
C57BL/6J	C57BL/6J-429	Private Strain	10	329	61.1	45	37.8
C57BL/6J	C57BL/6J-430	Private Strain	32	1064	73.7	.	.

C57BL/6J	C57BL/6J-431	Private Strain	10	422	82.2	36	25.0
C57BL/6J	C57BL/6J-432	Private Strain	10	321	55.8	50	36.0
C57BL/6J	C57BL/6J-433	Private Strain	10	284	65.1	43	37.2
C57BL/6J	C57BL/6J-434	Private Strain	20	406	66.7	41	24.4
C57BL/6J	C57BL/6J-435	Private Strain	29	473	57.7	.	.
C57BL/6J	C57BL/6J-436	Private Strain	70	1990	70.5	.	.
C57BL/6J	C57BL/6J-437	Private Strain	22	345	67.8	.	.
C57BL/6J	C57BL/6J-438	Private Strain	20	410	82.7	.	.
C57BL/6J	C57BL/6J-439	Private Strain	20	628	59.4	.	.
C57BL/6J	C57BL/6J-440	Private Strain	32	1219	25.6	.	.
C57BL/6J	C57BL/6J-441	Private Strain	30	1014	53.7	.	.
C57BL/6J	C57BL/6J-442	Private Strain	6	184	48.9	.	.
C57BL/6J	C57BL/6J-443	Private Strain	40	1255	87.9	.	.
C57BL/6J	C57BL/6J-444	Private Strain	20	673	73.1	.	.
C57BL/6J	C57BL/6J-445	Private Strain	40	1298	34.7	.	.
C57BL/6J	C57BL/6J-446	Private Strain	5	113	70.8	.	.
C57BL/6J	C57BL/6J-447	Private Strain	20	795	71.9	.	.
C57BL/6J	C57BL/6J-448	Private Strain	22	534	71.7	.	.
C57BL/6J	C57BL/6J-449	Private Strain	10	178	76.4	.	.
C57BL/6J	C57BL/6J-450	Private Strain	12	347	65.4	.	.
C57BL/6J	C57BL/6J-451	Private Strain	83	2223	42.8	.	.
C57BL/6J	C57BL/6J-452	Private Strain	20	559	81.2	.	.
C57BL/6J	C57BL/6J-453	Private Strain	20	577	75.7	.	.
C57BL/6J	C57BL/6J-454	Private Strain	25	560	55.4	.	.
C57BL/6J	C57BL/6J-455	Private Strain	22	577	71.2	.	.
C57BL/6J	C57BL/6J-456	Private Strain	20	445	76.4	.	.
C57BL/6J	C57BL/6J-457	Private Strain	22	671	78.4	.	.
C57BL/6J	C57BL/6J-458	Private Strain	31	592	46.1	17	41.7
C57BL/6J	C57BL/6J-459	Private Strain	20	576	57.5	.	.
C57BL/6J	C57BL/6J-460	Private Strain	19	592	80.6	24	35.3
C57BL/6J	C57BL/6J-461	Private Strain	20	624	50.5	19	50.0
C57BL/6J	C57BL/6J-462	Private Strain	25	588	81.0	.	.
C57BL/6J	C57BL/6J-463	Private Strain	22	668	74.9	.	.
C57BL/6J	C57BL/6J-464	Private Strain	22	651	81.3	.	.
C57BL/6J	C57BL/6J-465	Private Strain	20	723	73.3	.	.
C57BL/6J	C57BL/6J-466	Private Strain	20	805	63.7	.	.
C57BL/6J	C57BL/6J-467	Private Strain	22	532	80.1	.	.
C57BL/6J	C57BL/6J-468	Private Strain	20	651	79.0	.	.
C57BL/6J	C57BL/6J-469	Private Strain	20	647	40.2	.	.
C57BL/6J	C57BL/6J-47	Private Strain	10	260	51.5	.	.
C57BL/6J	C57BL/6J-470	Private Strain	20	731	79.9	.	.
C57BL/6J	C57BL/6J-471	Private Strain	20	480	69.8	.	.
C57BL/6J	C57BL/6J-472	Private Strain	20	531	59.5	.	.
C57BL/6J	C57BL/6J-473	Private Strain	20	500	58.6	.	.
C57BL/6J	C57BL/6J-474	Private Strain	40	1034	0.5	.	.
C57BL/6J	C57BL/6J-475	Private Strain	21	497	83.1	98	10.5
C57BL/6J	C57BL/6J-476	Private Strain	20	391	66.2	.	.
C57BL/6J	C57BL/6J-477	Private Strain	20	588	82.7	.	.
C57BL/6J	C57BL/6J-478	Private Strain	20	652	71.2	.	.
C57BL/6J	C57BL/6J-479	Private Strain	25	1057	55.3	24	26.5
C57BL/6J	C57BL/6J-48	Private Strain	10	426	59.4	.	.
C57BL/6J	C57BL/6J-480	Private Strain	20	900	85.2	88	25.0
C57BL/6J	C57BL/6J-481	Private Strain	20	748	67.9	.	.
C57BL/6J	C57BL/6J-482	Private Strain	40	565	1.8	.	.
C57BL/6J	C57BL/6J-483	Private Strain	41	1160	29.7	.	.
C57BL/6J	C57BL/6J-484	Private Strain	20	604	70.2	.	.
C57BL/6J	C57BL/6J-485	Private Strain	20	778	32.1	.	.
C57BL/6J	C57BL/6J-486	Private Strain	20	886	71.3	.	.
C57BL/6J	C57BL/6J-487	Private Strain	40	1407	53.0	.	.
C57BL/6J	C57BL/6J-488	Private Strain	30	884	52.9	38	31.8
C57BL/6J	C57BL/6J-489	Private Strain	20	567	72.7	.	.
C57BL/6J	C57BL/6J-490	Private Strain	30	766	65.4	224	36.8
C57BL/6J	C57BL/6J-491	Private Strain	12	437	78.5	.	.
C57BL/6J	C57BL/6J-492	Private Strain	41	1260	54.8	.	.
C57BL/6J	C57BL/6J-493	Private Strain	20	683	57.8	.	.
C57BL/6J	C57BL/6J-494	Private Strain	20	464	67.5	.	.
C57BL/6J	C57BL/6J-495	Private Strain	25	928	57.8	.	.
C57BL/6J	C57BL/6J-496	Private Strain	70	2202	59.6	40	22.6
C57BL/6J	C57BL/6J-497	Private Strain	20	791	76.1	84	35.0
C57BL/6J	C57BL/6J-498	Private Strain	30	872	51.2	26	4.2
C57BL/6J	C57BL/6J-499	Private Strain	20	827	41.1	53	53.8
C57BL/6J	C57BL/6J-500	Private Strain	20	649	63.0	.	.
C57BL/6J	C57BL/6J-501	Private Strain	20	756	59.1	.	.
C57BL/6J	C57BL/6J-502	Private Strain	20	692	59.2	.	.
C57BL/6J	C57BL/6J-503	Private Strain	20	666	20.3	.	.
C57BL/6J	C57BL/6J-504	Private Strain	12	383	38.9	.	.
C57BL/6J	C57BL/6J-505	Private Strain	26	649	56.3	.	.
C57BL/6J	C57BL/6J-506	Private Strain	22	567	50.4	.	.
C57BL/6J	C57BL/6J-507	Private Strain	20	696	42.8	19	7.7
C57BL/6J	C57BL/6J-508	Private Strain	20	852	73.0	21	36.8
C57BL/6J	C57BL/6J-509	Private Strain	22	557	45.4	21	28.6
C57BL/6J	C57BL/6J-510	Private Strain	10	282	66.7	.	.
C57BL/6J	C57BL/6J-511	Private Strain	22	749	66.5	.	.
C57BL/6J	C57BL/6J-512	Private Strain	60	1811	8.9	.	.
C57BL/6J	C57BL/6J-513	Private Strain	20	709	68.0	25	57.1
C57BL/6J	C57BL/6J-514	Private Strain	20	704	74.9	.	.
C57BL/6J	C57BL/6J-515	Private Strain	20	651	63.4	42	20.0
C57BL/6J	C57BL/6J-516	Private Strain	20	373	59.6	.	.
C57BL/6J	C57BL/6J-517	Private Strain	10	318	65.4	.	.
C57BL/6J	C57BL/6J-518	Private Strain	20	717	79.8	.	.
C57BL/6J	C57BL/6J-519	Private Strain	21	764	36.1	48	13.6
C57BL/6J	C57BL/6J-520	Private Strain	20	675	64.3	.	.
C57BL/6J	C57BL/6J-521	Private Strain	20	613	76.7	.	.
C57BL/6J	C57BL/6J-522	Private Strain	30	845	49.6	.	.
C57BL/6J	C57BL/6J-523	Private Strain	20	543	76.2	.	.
C57BL/6J	C57BL/6J-524	Private Strain	10	203	19.2	.	.
C57BL/6J	C57BL/6J-525	Private Strain	11	347	74.1	.	.
C57BL/6J	C57BL/6J-526	Private Strain	8	136	88.2	.	.
C57BL/6J	C57BL/6J-527	Private Strain	10	288	53.5	.	.
C57BL/6J	C57BL/6J-528	Private Strain	10	397	67.0	.	.
C57BL/6J	C57BL/6J-529	Private Strain	30	980	2.6	.	.
C57BL/6J	C57BL/6J-530	Private Strain	45	1159	40.6	.	.
C57BL/6J	C57BL/6J-531	Private Strain	20	414	76.1	.	.

C57BL/6J	C57BL/6J-532	Private Strain	55	1772	16.7	.	.
C57BL/6J	C57BL/6J-533	Private Strain	20	742	49.5	.	.
C57BL/6J	C57BL/6J-534	Private Strain	20	592	27.9	.	.
C57BL/6J	C57BL/6J-535	Private Strain	23	654	51.5	.	.
C57BL/6J	C57BL/6J-536	Private Strain	10	323	56.0	.	.
C57BL/6J	C57BL/6J-537	Private Strain	31	791	59.9	.	.
C57BL/6J	C57BL/6J-538	Private Strain	10	155	84.5	.	.
C57BL/6J	C57BL/6J-539	Private Strain	10	252	63.5	.	.
C57BL/6J	C57BL/6J-540	Private Strain	21	537	65.2	.	.
C57BL/6J	C57BL/6J-541	Private Strain	39	875	77.2	.	.
C57BL/6J	C57BL/6J-542	Private Strain	20	429	48.0	.	.
C57BL/6J	C57BL/6J-543	Private Strain	25	410	50.5	.	.
C57BL/6J	C57BL/6J-544	Private Strain	20	355	66.5	.	.
C57BL/6J	C57BL/6J-545	Private Strain	20	695	65.5	.	.
C57BL/6J	C57BL/6J-546	Private Strain	20	663	34.1	.	.
C57BL/6J	C57BL/6J-547	Private Strain	20	809	69.1	.	.
C57BL/6J	C57BL/6J-548	Private Strain	20	389	23.4	.	.
C57BL/6J	C57BL/6J-549	Private Strain	21	752	52.5	.	.
C57BL/6J	C57BL/6J-56	Private Strain	6	216	77.8	.	.
C57BL/6J	C57BL/6J-78	Private Strain	10	285	85.6	.	.
C57BL/6J	2267	STOCK Bdnf<tm1Jae>/J	40	1280	39.5	78	23.1
C57BL/6J	6001	STOCK Dicer1<tm1Bdh>/J	16	657	73.4	.	.
C57BL/6J	6331	STOCK Gt(ROSA)26Sor<tm1(DTA)Jpmb>/J	10	225	72.9	.	.
C57BL/6J	6241	STOCK Hhip<tm1Amc>/J	9	302	64.9	.	.
C57BL/6J	5994	STOCK Mbtps1<tm1Jdh>/J	10	410	83.2	.	.
C57BL/6J	6951	STOCK Notch1<tm2Rko>/GridJ	33	1068	17.2	.	.
C57BL/6J	6327	STOCK Pcsk1<tm1Dfs>/J	10	248	27.8	.	.
C57BL/6J	5740	STOCK Ppid<tm1.1Mmos>/J	20	491	82.5	.	.
C57BL/6J	5737	STOCK Ppid<tm1Mmos>/J	25	958	59.5	.	.
C57BL/6J	6083	STOCK Sfpi1<tm1.3Dgt>/J	10	208	61.5	.	.
C57BL/6J	6882	STOCK Tg(ACTB-Bgeo,-AML1/ETO,-ALPP)1Lbe/J	8	297	80.8	.	.
C57BL/6J	6876	STOCK Tg(ACTB-Bgeo,-TEL/AML1,-EGFP)A6Lbe/J	12	175	45.7	.	.
C57BL/6J	6613	STOCK Tg(ACTB-Bgeo,-Tle1,-ALPP)1Lbe/J	10	304	59.5	.	.
C57BL/6J	5645	STOCK Tg(ACTB-mRFP1)1F1Hadj/J	10	225	36.4	.	.
C57BL/6J	5854	STOCK Tg(Cp-EGFP)25Gaia/J	95	1404	55.0	76	26.3
C57BL/6J	5938	STOCK Tg(Eno2-cre)39Jme/J	10	275	70.2	.	.
C57BL/6J	6334	STOCK Tg(Gad1-EGFP)94Agmo/J	15	566	78.3	.	.
C57BL/6J	6340	STOCK Tg(Gad1-EGFP)98Agmo/J	15	582	64.4	.	.
C57BL/6J	6866	STOCK Tg(Ins1-DsRed*T4)32Hara/J	8	254	71.7	.	.
C57BL/6J	6784	STOCK Tg(Ins1-ECFP)24Hara/J	8	295	17.6	.	.
C57BL/6J	6207	STOCK Tg(Pcp2-cre)1Amc/J	8	254	48.8	.	.
C57BL/6J	6395	STOCK Tg(Sim1-cre)1Lowl/J	10	312	84.3	.	.
C57BL/6J	5375	STOCK Trp53bp1<hlb543>/CloJ	50	1051	70.3	.	.
C57BLKS/J	4176	BKS.B6-Tub<tub>/Jng	30	207	1.0		
C57BLKS/J	C57BLKS/J-1	Private Strain	10	72	26.4		
C57BLKS/J	C57BLKS/J-2	Private Strain	80	706	6.0	60	45.0
DBA/2J	671	DBA/2J	19	585	90.0	96	33.3
DBA/2J	1594	DBA/2J-Dtnbp1<sdy>/J	70	1438	84.5	680	12.0
DBA/2J	548	DBA/2J-Grid2<ho-4J>/J	40	503	86.7	50	48.0
DBA/2J	DBA/2J-4	Private Strain	10	205	70.7	.	.
DBA/2J	DBA/2J-5	Private Strain	15	274	72.3	.	.
DBA/2J	DBA/2J-6	Private Strain	15	355	45.9	.	.
DBA/2J	DBA/2J-7	Private Strain	10	144	63.2	.	.
FVB/NJ	5989	129;FVB-Tg(PTH-cre)4167Slib/J	45	1017	86.1	38	31.6
FVB/NJ	5564	FVB(Cg)-Tg(Ins2-CALM1)26Ove Tg(Cryaa-TAg)1Ove/PneJ	199	3635	46.0	.	.
FVB/NJ	2899	FVB.129S2(B6)-Trp53<tm1Tyj>/J	100	1522	61.9	412	24.1
FVB/NJ	6206	FVB.129S6-Gt(ROSA)26Sor<tm1(HIF1A/luc)Kael>/J	12	88	44.3	.	.
FVB/NJ	5710	FVB.129S-Mmp13<tm1Werb>/J	15	228	89.0	.	.
FVB/NJ	6867	FVB.B6-Ins2<Akita>/MlnJ	15	255	94.1	.	.
FVB/NJ	7031	FVB.Cg-Krt8<tm1Rgo>/J	15	125	72.8	.	.
FVB/NJ	4363	FVB.Cg-Tg(MMTV-vHaras)SH1Led/J	180	2738	20.2	.	.
FVB/NJ	3170	FVB.Cg-Tg(Myh6-tTA)6Smbf/J	50	630	10.6	.	.
FVB/NJ	6222	FVB.Cg-Tg(Scgb1a1-rtTA)1Jaw/J	9	96	63.5	.	.
FVB/NJ	6225	FVB.Cg-Tg(SFTPC-rtTA)5Jaw/J	10	139	86.3	.	.
FVB/NJ	5026	FVB.Cg-Tg(SMN2)89Ahmb Tg(SMN1*A2G)2023Ahmb Smn1<tm1Msd>/J	40	538	8.7	.	.
FVB/NJ	6209	FVB.Cg-Tg(Tal1-tTA)19Dgt/J	10	177	83.6	.	.
FVB/NJ	6224	FVB.Cg-Tg(tetO-cre)1Jaw/J	10	153	91.5	.	.
FVB/NJ	2934	FVB.Cg-Tg(Wnt1)1Hev/J	65	641	77.3	682	27.6
FVB/NJ	1800	FVB/NJ	23	351	88.3	65	37.7
FVB/NJ	3640	FVB/NJ-Tg(YAC72)2511Hay/J	50	671	27.1	.	.
FVB/NJ	3753	FVB/N-Tg(Eno2CDK5R1)1Jdm/J	50	747	86.5	26	15.4
FVB/NJ	2374	FVB/N-Tg(MMTV-PyVT)634Mul/J	205	3116	90.2	3265	27.4
FVB/NJ	5942	FVB/N-Tg(Pf4-tTA/VP16)42Kra/J	60	872	87.4	84	21.4
FVB/NJ	5941	FVB/N-Tg(tetO-Aurkb,lacZ)41Kra/J	40	630	91.7	35	40.0
FVB/NJ	6202	FVB/N-Tg(tetO-BCR/ABL1)2Dgt/J	10	153	66.7	.	.
FVB/NJ	6143	FVB/N-Tg(Thy1-cre)1Vln/J	40	434	82.3	25	68.0
FVB/NJ	3127	FVB;129P2-Bcl3<tm1Ver>/J	40	686	84.8	.	.
FVB/NJ	4066	FVB;129S-Men1<tm1.1Ctre>/J	40	587	79.6	.	.
FVB/NJ	6655	FVB-Tg(ACTA1-PABPN1*A17)1Drub/J	10	118	96.6	.	.
FVB/NJ	6405	FVB-Tg(Ckmm-cre)5Khn/J	12	195	88.7	.	.
FVB/NJ	6125	FVB-Tg(H2-D-Il15)3304Clgr/J	10	138	80.4	.	.
FVB/NJ	5625	FVB-Tg(Pcp2-tTA)3Horr/J	20	212	54.3	.	.
FVB/NJ	6439	FVB-Tg(tetO/CMV-KRAS*G12C)9.1Msmi/J	10	115	65.2	.	.
FVB/NJ	FVB/NJ-17	Private Strain	65	984	66.2	78	30.8
FVB/NJ	FVB/NJ-18	Private Strain	50	730	82.3	41	22.0
FVB/NJ	FVB/NJ-19	Private Strain	50	844	89.0	38	13.2
FVB/NJ	FVB/NJ-20	Private Strain	10	120	75.0	.	.
FVB/NJ	FVB/NJ-21	Private Strain	15	229	90.8	.	.
FVB/NJ	FVB/NJ-22	Private Strain	30	326	81.9	.	.
FVB/NJ	FVB/NJ-23	Private Strain	10	126	97.6	.	.
FVB/NJ	FVB/NJ-24	Private Strain	10	100	76.0	.	.
FVB/NJ	FVB/NJ-25	Private Strain	30	339	87.0	.	.
FVB/NJ	FVB/NJ-26	Private Strain	12	118	77.1	.	.
FVB/NJ	FVB/NJ-27	Private Strain	10	146	76.7	.	.
FVB/NJ	FVB/NJ-28	Private Strain	10	165	99.4	.	.
FVB/NJ	FVB/NJ-29	Private Strain	90	1391	85.5	48	33.3
FVB/NJ	FVB/NJ-30	Private Strain	90	1313	88.1	54	48.1
FVB/NJ	FVB/NJ-31	Private Strain	44	562	27.9	43	20.9
FVB/NJ	FVB/NJ-32	Private Strain	45	596	89.4	48	20.8
FVB/NJ	FVB/NJ-33	Private Strain	20	380	93.4	.	.
FVB/NJ	FVB/NJ-34	Private Strain	45	611	91.3	34	35.3
FVB/NJ	FVB/NJ-35	Private Strain	60	875	60.1	62	19.4
FVB/NJ	FVB/NJ-36	Private Strain	45	662	77.5	38	42.1

FVB/NJ	FVB/NJ-37	Private Strain	45	504	84.7	32	50.0
FVB/NJ	FVB/NJ-38	Private Strain	45	630	79.7	36	13.9
FVB/NJ	FVB/NJ-39	Private Strain	45	606	93.6	32	37.5
FVB/NJ	FVB/NJ-40	Private Strain	44	701	80.2	30	6.7
FVB/NJ	FVB/NJ-41	Private Strain	86	1180	7.2	50	12.0
FVB/NJ	FVB/NJ-42	Private Strain	20	289	49.5	.	.
FVB/NJ	FVB/NJ-43	Private Strain	20	247	82.6	.	.
FVB/NJ	FVB/NJ-44	Private Strain	20	218	61.0	.	.
FVB/NJ	FVB/NJ-45	Private Strain	20	222	91.0	.	.
FVB/NJ	FVB/NJ-46	Private Strain	20	359	79.4	.	.
FVB/NJ	FVB/NJ-47	Private Strain	20	372	77.4	.	.
FVB/NJ	FVB/NJ-48	Private Strain	76	864	48.5	64	37.5
FVB/NJ	FVB/NJ-49	Private Strain	10	103	88.3	.	.
FVB/NJ	7029	STOCK Krt18<tm1Tmm>/J	10	101	42.6	.	.
FVB/NJ	5965	STOCK Tg(Pomc1-cre)16Lowl/J	79	1122	88.3	62	29.0
NOD/ShiLtJ	6355	NOD.129-Btla<tm1Kmm>/J	10	185	42.7		
NOD/ShiLtJ	5036	NOD.129S2(B6)-Ins2<tm1Jja>/GseJ	60	1126	49.9		
NOD/ShiLtJ	6698	NOD.Cg-Il4<tm1Lky>/JbsJ	10	168	81.0		
NOD/ShiLtJ	5853	NOD.Cg-Tg(HLA-A2/H2-K)1Scr/ShrmJ	50	1150	67.8	116	19.0
NOD/ShiLtJ	6254	NOD.Cg-Tg(Ins2-Ccl21b)2Cys/JbsJ	40	704	0.3		
NOD/ShiLtJ	6154	NOD.Cg-Tg(Ins2-Cxcl13)1Cys/JbsJ	40	960	71.5	38	36.8
NOD/ShiLtJ	5868	NOD.Cg-Tg(TcraTcrbNY8.3)1Pesa/DvsJ	40	817	68.2	38	47.4
NOD/ShiLtJ	5686	NOD.Cg-Thy1<a> Tg(TcraCl4,TcrbCl4)1Shrm/ShrmJ	40	1174	61.7		
NOD/ShiLtJ	6303	NOD.FVB-Tg(TcraBDC12-4.1)10Jos/GseJ	10	203	64.5		
NOD/ShiLtJ	6304	NOD.FVB-Tg(TcrbBDC12-4.1)82Gse/GseJ	10	212	63.2		
NOD/ShiLtJ	1976	NOD/ShiLtJ	40	1008	87.4	204	30.8
NOD/ShiLtJ	5082	NOD/ShiLt-Tg(ACTB-Ica1/EGFP)18Mdos/MdosJ	30	477	44.4		
NOD/ShiLtJ	6778	NOD/ShiLt-Tg(Gfap-Cd80)9Mdos/MdosJ	12	279	18.6		
NOD/ShiLtJ	6777	NOD/ShiLt-Tg(Ins2-Cd274)2Mdos/MdosJ	12	275	38.2		
NOD/ShiLtJ	3074	NOD/ShiLt-Tg(Ins2-GAD2)1Lt/LtJ	40	874	77.3	38	44.7
NOD/ShiLtJ	5739	NOD-Tg(H2-Ea-Ins2)1Wehi/WehiJ	30	742	81.3		
NOD/ShiLtJ	NOD/ShiLtJ-1	Private Strain	8	137	77.4		
NOD/ShiLtJ	NOD/ShiLtJ-2	Private Strain	40	714	70.9		
NOD/ShiLtJ	NOD/ShiLtJ-3	Private Strain	31	541	69.7	25	40.0
NOD/ShiLtJ	NOD/ShiLtJ-4	Private Strain	6	118	82.2		
NOD/ShiLtJ	NOD/ShiLtJ-5	Private Strain	8	93	79.6		
