# Supplementary material for: Conserving, Distributing and Managing Genetically Modified Mouse Lines by Sperm Cryopreservation
Source: PLoS One. 2008 Jul 30;3(7):e2792. doi: 10.1371/journal.pone.0002792 (PMC2453316; doi:10.1371/journal.pone.0002792)
Supplement: Table S3 — Calibration parameters used with Hamilton Thorne IVOS computerized semen analyzer (Beverly, MA). (0.03 MB DOC) [file pone.0002792.s005.doc]

**Table S3**. Calibration parameters used with Hamilton Thorne IVOS computerized semen analyzer (Beverly, MA)

Parameter Assigned Values

Apply Sort: 2

Frames acquired: 30

Frame rate: 60 Hz

Minimum contrast: 30

Minimum Cell Size: 4 pixels

Minimum Static Contrast: 15

Straightness (STR), Threshold: 50%

Low VAP Cutoff: 10.0 µm/s

Medium VAP Cutoff: 50.0 µm/s

Low VSL Cutoff: 0.0 µm/s

Head Size, Non-Motile: 13 pixels

Head Intensity, Non-Motile: 75

Static Head Size: 0.57 to 2.91

Static Head Intensity: 0.14 to 1.84

Static Elongation: 0 to 87

Slow Cells Motile: YES

Magnification: 0.82

Video Source: Camera

Video Frequency: 60

Bright Field; No

Brightness for LED: 2663

Brightness for Ident: 3000

Temperature, Set: 37°C

Cell Type: User

Cell Depth Setup: 100.0µm

Field Selection Mode: SELECT

Indent Active: NO

Ident Mode: B

Integrating Time: 1 Frames

Optics

Video Gain: Medium

Video Brightness: 2300

Video Contrast: 40

Video Sync Level: FW Sync_Level_100

Video Vertical Sync: 75

Sort # 2

Points in Track: 16 to 100
