# Supplementary material for: Conserving, Distributing and Managing Genetically Modified Mouse Lines by Sperm Cryopreservation
Source: PLoS One. 2008 Jul 30;3(7):e2792. doi: 10.1371/journal.pone.0002792 (PMC2453316; doi:10.1371/journal.pone.0002792)
Supplement: Table S4 — Data set for sperm cryopreserved and recovered using a method modified from Sztein et al. (0.02 MB PDF) [file pone.0002792.s006.pdf]

Table S4. ***Data set for sperm cryopreserved and recovered using a method modified from Sztein et al.***

The first row of the table details the data within each column. The first column depicts the inbred strain that was selected for oocyte donation. The second column represents the stock/accession number for each strain. For public strains, the stock number can be used to obtain detailed strain information by searching the JAX® Mice Database (http://jaxmice.jax.org/query/f?p=205:1:1867898574872980466). Accession numbers were randomly created for private strains, to maintain confidentiality.

The third column shows the strain name, which details the genetic background and genetic modification of the strain (http://www.informatics.jax.org/mgihome/nomen/). The fourth, fifth, and sixth columns indicate the number of females and oocytes used for in vitro fertilization and the proportion of oocytes developing into 2-cell embryos for that strain.

Oocyte Donor	Stock / Acc #	Strain Name	# females	# oocytes	% 2-cell
129S1/SvImJ	3204	129/Sv-Lyn<tm1Sor>/J	5	128	0.8
129S1/SvImJ	2485	129-Alox5<tm1Fun>/J	33	803	4.1
129S1/SvImJ	2484	129-Alpl<tm1Sor>/J	28	464	1.3
129S1/SvImJ	4374	129-Npr1<tm1Gar>/J	19	664	2.8
129S1/SvImJ	3082	129S1/SvImJ-Bcl2<tm1Mpin>/J	37.5	1019	6.3
129S1/SvImJ	2753	129S6/SvEvTac-Atm<tm1Awb>/J	6	95	4.2
129S1/SvImJ	3310	129S-Gt(ROSA)26Sor<tm1Sor>/J	24	298	2.0
129S1/SvImJ	3117	129S-Sst<tm1Ute>/J	24	584	1.8
129S1/SvImJ	129S1/SvImJ-18	Private Strain	61	1588	1.3
129S1/SvImJ	129S1/SvImJ-19	Private Strain	63	1452	3.3
129S1/SvImJ	129S1/SvImJ-20	Private Strain	10	302	3.7
129S1/SvImJ	129S1/SvImJ-21	Private Strain	18	590	1.4
129S1/SvImJ	2913	STOCK Gria2<tm1Rod>/J	26	773	10.0
B6129SF1/J	3461	B6.129P2-Thra<tm1Ven>/J	6	184	41.3
B6129SF1/J	2727	B6;129-Ahr<tm1Bra>/J	6	79	0.0
B6129SF1/J	4177	B6;129-Cd3e<tm1Lov>/J	22	500	11.9
B6129SF1/J	3536	B6;129-Cdk5<tm1Kul>/J	12	316	16.8
B6129SF1/J	4264	B6;129-Cycs<tm1Wlm>/J	20	517	17.6
B6129SF1/J	3137	B6;129-Gabrg2<tm1Geh>/J	28	709	19.7
B6129SF1/J	4425	B6;129-Gdf1<tm1Sjl>/J	6	197	37.6
B6129SF1/J	3535	B6;129-Gla<tm1Kul>/J	43.5	1293	30.8
B6129SF1/J	3121	B6;129-Grm5<tm1Rod>/J	40	1203	4.4
B6129SF1/J	3504	B6;129-Gt(ROSA)26Sor<tm1Sho>/J	6	92	7.6
B6129SF1/J	4077	B6;129-Gt(ROSA)26Sor<tm2Sho>/J	6	184	4.3
B6129SF1/J	3138	B6;129-Hprt1<tm1Detl>/J	20	625	9.6
B6129SF1/J	3900	B6;129-Irs3<tm1Lhd>/J	48	1013	13.3
B6129SF1/J	3901	B6;129-Irs4<tm1Lhd>/J	12	261	7.0
B6129SF1/J	3530	B6;129-Ltb<tm1Flv>/J	6	104	26.9
B6129SF1/J	3267	B6;129-Mar1<tm1Wtg>/J	8	148	40.5
B6129SF1/J	2398	B6;129P2-Csf3<tm1Ard>/J	18	511	3.0
B6129SF1/J	2847	B6;129P2-Fcer1g<tm1Rav>/J	18	495	9.4
B6129SF1/J	3253	B6;129P-Hbb-b1<tm1Unc> Hbb-b2<tm1Unc>/J	9	236	40.9
B6129SF1/J	2265	B6;129S2-Bcl2<tm1Sjk>/J	10	286	21.0
B6129SF1/J	2428	B6;129S2-Cd40lg<tm1Imx>/J	13	364	2.2
B6129SF1/J	2473	B6;129S2-Cdh1<tm1Kem>/J	5	174	10.3
B6129SF1/J	3263	B6;129S2-Cdkn1a<tm1Tyj>/J	6	217	17.5
B6129SF1/J	3374	B6;129S2-H2<dlAb1-Ea>/J	6	159	0.6
B6129SF1/J	3379	B6;129S2-Scarb1<tm1Kri>/J	10	388	6.9
B6129SF1/J	3366	B6;129S4-Cux1<tm1Ejn>/J	8	277	4.8
B6129SF1/J	2914	B6;129S4-Hexb<tm1Rlp>/J	36.5	1017	13.3
B6129SF1/J	3751	B6;129S4-Igh-6<tm1Che>/J	49.5	1084	6.1
B6129SF1/J	2896	B6;129S4-Soat1<tm1Far>/J	30	851	29.8
B6129SF1/J	3749	B6;129S5-Cpsf3<Gt(VICTR20)3Lex>/J	2	56	37.5
B6129SF1/J	4373	B6;129S6-Apaf1<tm1Her>/J	14	409	14.6
B6129SF1/J	3524	B6;129S6-Lrp8<tm1Her>/J	12	377	33.8
B6129SF1/J	2833	B6;129S6-Stat5a<tm1Mam>/J	16	624	7.9
B6129SF1/J	3277	B6;129S7-Acvr2a<tm1Zuk>/J	16	424	2.7
B6129SF1/J	2751	B6;129S7-Cyp7a1<tm1Rus>/J	5	107	30.8
B6129SF1/J	3283	B6;129S7-Fshb<tm1Zuk>/J	4	137	6.6
B6129SF1/J	3120	B6;129S7-L1cam<tm1Sor>/J	12	415	47.9
B6129SF1/J	2972	B6;129S7-Sod1<tm1Leb>/J	30	761	3.9
B6129SF1/J	2529	B6;129S7-Vldlr<tm1Her>/J	10	309	19.4
B6129SF1/J	2536	B6;129S-Btk<tm1Wk>/J	11	289	20.7

B6129SF1/J	4234	B6;129S-Fgfr3<tm1Dor>/J	15	394	13.1
B6129SF1/J	2642	B6;129S-Gfap<tm1Mes>/J	17	519	1.1
B6129SF1/J	4153	B6;129S-Mtap7<Gt(ROSABetageo)1Sor>/J	16	284	2.8
B6129SF1/J	3902	B6;129S-Mttp<tm2Sgy>/J	20	651	8.1
B6129SF1/J	3807	B6;129S-Sele<tm1Hyn> Sell<tm1Hyn> Selp<tm1Hyn>/J	5	171	12.3
B6129SF1/J	3728	B6;129S-Sparc<tm1Hwe>/J	17	371	18.9
B6129SF1/J	3789	B6;129S-Vmn2r10<Gt(VICTR20)23Lex>/J	0	43	72.1
B6129SF1/J	3723	B6;129X1-Il15ra<tm1Ama>/J	6	173	13.3
B6129SF1/J	3552	B6129-Tg(Wap-cre)11738Mam/J	29	935	37.3
B6129SF1/J	B6129SF1/J-1	Private Strain	7	133	5.3
B6129SF1/J	B6129SF1/J-17	Private Strain	8	208	13.5
B6129SF1/J	B6129SF1/J-18	Private Strain	21	568	6.4
B6129SF1/J	B6129SF1/J-19	Private Strain	25	512	15.4
B6129SF1/J	B6129SF1/J-20	Private Strain	8	260	20.4
B6129SF1/J	B6129SF1/J-21	Private Strain	6	114	1.8
B6129SF1/J	B6129SF1/J-22	Private Strain	19	626	16.7
B6129SF1/J	2512	STOCK Adra2c<tm1Gsb>/J	12	335	7.2
B6129SF1/J	3463	STOCK Aga<tm1Vk>/J	4	132	4.5
B6129SF1/J	3899	STOCK Cd44<tm1Hbg>/J	6	103	11.7
B6129SF1/J	2364	STOCK Cftr<tm1Unc>-Tg(FABPCFTR)1Jaw/J	14	366	11.0
B6129SF1/J	3582	STOCK Cp<tm1Hrs>/J	10	347	63.7
B6129SF1/J	3534	STOCK Inpp5d<tm1Dmt>/J	10	245	22.4
B6129SF1/J	2403	STOCK Mag<tm1Rod>/J	50	1490	18.0
B6129SF1/J	3318	STOCK Shh<tm1Amc>/J	23	676	12.3
B6129SF1/J	3773	STOCK Tg(ACTB-ECFP)CK6Nagy/J	20	453	10.6
B6129SF1/J	3551	STOCK Tg(MMTV-cre)1Mam/J	40	1310	26.5
B6129SF1/J	3102	STOCK Tgfb2<tm1Doe>/J	8	260	27.3
BALB/cBy	2088	BALB/cByJ-Herc2<J>/J	15	185	15.7
BALB/cBy	4122	BALB/cByJ-Nmf31/J	7.5	166	7.8
BALB/cBy	BALB/cByJ-26	Private Strain	12	282	13.7
BALB/cBy	BALB/cByJ-37	Private Strain	10	233	0.9
BALB/cBy	BALB/cByJ-38	Private Strain	26	395	3.3
BALB/cJ	3514	BALB/c-Il4ra<tm1Sz>/J	6	45	28.9
BALB/cJ	2420	C.129P2(B6)-B2m<tm1Unc>/J	6	54	9.3
BALB/cJ	2538	C.129P2(B6)-Igh-5<tm1Cgn>/J	10	106	17.0
BALB/cJ	2691	C.129S1(B6)-Il12a<tm1Jm>/J	6	64	6.3
BALB/cJ	3017	C.129S1-Il12rb1<tm1Jm>/J	43	488	17.4
BALB/cJ	2667	C.129S2(B6)-Cd28<tm1Mak>/J	6	59	1.7
BALB/cJ	3238	C.129S2(B6)-Ciita<tm1Ccum>/J	34	569	6.0
BALB/cJ	2327	C.129S2-Plat<tm1Mlg>/J	9	140	17.5
BALB/cJ	3830	C.129S4(B6)-Mif<tm1Dvd>/J	16	141	21.1
BALB/cJ	3145	C.129S7(B6)-Rag1<tm1Mom>/J	6	96	4.2
BALB/cJ	2955	C.129S7-Gt(ROSA)26Sor/J	14	136	11.6
BALB/cJ	3303	C.Cg-Tg(DO11.10)10Dlo/J	3	39	43.6
C57BL/6J	3916	B6(Cg)-Col2a1<sedc>/J	30	772	2.6
C57BL/6J	3716	B6.129-Abcd1<tm1Kan>/J	48	1205	3.9
C57BL/6J	3557	B6.129-Adra2c<tm1Gsb>/J	31	788	3.1
C57BL/6J	2831	B6.129-Ahr<tm1Bra>/J	9	275	1.5
C57BL/6J	3509	B6.129-Blmh<tm1Geh>/J	12	322	0.3
C57BL/6J	4152	B6.129-Ctnnb1<tm2Kem>/KnwJ	6	62	0.0
C57BL/6J	2957	B6.129-Dll1<tm1Gos>/J	37	623	2.4
C57BL/6J	4478	B6.129-Foxd1<tm1Lai>/J	12.5	464	2.7
C57BL/6J	3725	B6.129-Gabrd<tm1Geh>/J	33	867	4.4
C57BL/6J	3619	B6.129-Grm4<tm1Hpn>/J	29	891	2.1
C57BL/6J	3558	B6.129-Grm5<tm1Rod>/J	79	1924	4.5
C57BL/6J	3360	B6.129-Jup<tm1Kem>/J	30	851	3.0
C57BL/6J	2938	B6.129-Kdr<tm1Jrt>/J	24	1365	9.7
C57BL/6J	2681	B6.129P2-Agt<tm1Unc>/J	59	1853	3.4
C57BL/6J	2682	B6.129P2-Agtr1a<tm1Unc>/J	21	895	4.7
C57BL/6J	2362	B6.129P2-Camk2a<tm1Sva>/J	131	4867	11.7
C57BL/6J	2687	B6.129P2-Ccl3<tm1Unc>/J	12	326	1.2
C57BL/6J	3727	B6.129P2-Cd38<tm1Lnd>/J	42	803	4.4
C57BL/6J	2928	B6.129P2-Cd40<tm1Kik>/J	17	640	8.5
C57BL/6J	4303	B6.129P2-F9<tm1Dws>/J	53	1936	23.7
C57BL/6J	3233	B6.129P2-Fas<tm1Osa>/J	6	188	38.8
C57BL/6J	3171	B6.129P2-Fcgr3<tm1Sjv>/J	19	324	1.7
C57BL/6J	3025	B6.129P2-Fmr1<tm1Cgr>/J	71	2209	3.9
C57BL/6J	2683	B6.129P2-Hbb-b1<tm1Unc> Hbb-b2<tm1Unc>/J	46	1822	4.2
C57BL/6J	3812	B6.129P2-Hfe<tm1Gfn>/J	29	903	2.4

C57BL/6J	2251	B6.129P2-Il10<tm1Cgn>/J	6	185	9.7
C57BL/6J	2252	B6.129P2-Il2<tm1Hor>/J	9	273	1.1
C57BL/6J	2816	B6.129P2-Il2rb<tm1Mak>/J	33	2205	1.8
C57BL/6J	2253	B6.129P2-Il4<tm1Cgn>/J	9	302	5.3
C57BL/6J	2405	B6.129P2-Ncam1<tm1Cgn>/J	27	1047	8.9
C57BL/6J	2609	B6.129P2-Nos2<tm1Lau>/J	9	240	2.1
C57BL/6J	2684	B6.129P2-Nos3<tm1Unc>/J	10	272	3.6
C57BL/6J	2685	B6.129P2-Nppa<tm1Unc>/J	18	490	1.6
C57BL/6J	2829	B6.129P2-Plaur<tm1Jld>/J	34	719	3.1
C57BL/6J	3918	B6.129P2-Pltp<tm1Jia>/J	23	713	1.4
C57BL/6J	3142	B6.129P2-Prlr<tm1Cnp>/J	13	260	4.3
C57BL/6J	2120	B6.129P2-Tcrd<tm1Mom>/J	10	282	6.2
C57BL/6J	3461	B6.129P2-Thra<tm1Ven>/J	13.5	412	3.6
C57BL/6J	2880	B6.129-Penk-rs<tm1Pig>/J	10	330	0.6
C57BL/6J	3615	B6.129-Psen1<tm1Shn>/J	23	754	3.2
C57BL/6J	2725	B6.129-Ptprc<tm1Holm>/J	12	290	14.3
C57BL/6J	2693	B6.129S1-Il12b<tm1Jm>/J	6	205	5.9
C57BL/6J	2984	B6.129S1-Il12rb1<tm1Jm>/J	21	671	5.5
C57BL/6J	3248	B6.129S1-Il12rb2<tm1Jm>/J	6	183	14.8
C57BL/6J	4322	B6.129S1-Mapk10<tm1Flv>/J	14	353	6.2
C57BL/6J	4319	B6.129S1-Mapk8<tm1Flv>/J	12	404	8.6
C57BL/6J	3462	B6.129S1-Thrb<tm1Df>/J	26.5	1294	3.0
C57BL/6J	3808	B6.129S2(Cg)-Prl<tm1Hmn>/J	19	508	4.7
C57BL/6J	2777	B6.129S2-Adra2a<tm1Lel>/J	84	2201	3.6
C57BL/6J	4042	B6.129S2-Alox12<tm1Fun>/J	10	229	12.5
C57BL/6J	2778	B6.129S2-Alox15<tm1Fun>/J	6	224	3.6
C57BL/6J	2612	B6.129S2-Bmp4<tm1Blh>/J	20	617	6.2
C57BL/6J	2770	B6.129S2-Cd40lg<tm1Imx>/J	30	873	2.5
C57BL/6J	3239	B6.129S2-Ciita<tm1Ccum>/J	6	186	5.4
C57BL/6J	3114	B6.129S2-Crh<tm1Maj>/J	96	3092	3.7
C57BL/6J	3190	B6.129S2-Drd2<tm1Low>/J	76	2532	5.0
C57BL/6J	4078	B6.129S2-F5<tm1Dgi>/J	12	496	2.4
C57BL/6J	2248	B6.129S2-Gzmb<tm1Ley>/J	8	534	7.6
C57BL/6J	4513	B6.129S2-H2-DMa<tm1Doi>/J	5	99	1.0
C57BL/6J	2288	B6.129S2-Igh-6<tm1Cgn>/J	6	163	12.9
C57BL/6J	3865	B6.129S2-Itgav<tm1Hyn>/J	5	102	26.5
C57BL/6J	2817	B6.129S2-Lck<tm1Mak>/J	23	645	5.8
C57BL/6J	2258	B6.129S2-Lta<tm1Dch>/J	11	228	5.3
C57BL/6J	3191	B6.129S2-Pomc1<tm1Low>/J	16	491	41.7
C57BL/6J	2944	B6.129S2-Tap1<tm1Arp>/J	27	662	2.6
C57BL/6J	2220	B6.129S2-Tgfb1<tm1Doe>/J	85	2246	6.6
C57BL/6J	2771	B6.129S2-Tlx1<tm1Sjk>/J	9	330	20.3
C57BL/6J	2266	B6.129S4-Bdnf<tm1Jae>/J	15	535	5.4
C57BL/6J	3641	B6.129S4-C3<tm1Crr>/J	10	320	14.2
C57BL/6J	3643	B6.129S4-C4b<tm1Crr>/J	18	368	28.0
C57BL/6J	3611	B6.129S4-Cd80<tm1Shr>/J	16	559	3.0
C57BL/6J	3609	B6.129S4-Cd86<tm1Shr>/J	6	196	1.5
C57BL/6J	4163	B6.129S4-Cdk5r1<tm1Lht>/J	6	6	0.0
C57BL/6J	3824	B6.129S4-Dgat1<tm1Far>/J	15	448	3.0
C57BL/6J	2198	B6.129S4-Dnmt1<tm1Jae>/J	15	517	5.5
C57BL/6J	2958	B6.129S4-Drd3<tm1Dac>/J	32	849	25.0
C57BL/6J	4067	B6.129S4-Ep300<tm1Dli>/J	35	1063	6.9
C57BL/6J	3474	B6.129S4-Gt(ROSA)26Sor<tm1Sor>/J	6	219	33.3
C57BL/6J	2867	B6.129S4-Icam1<tm1Jcgr>/J	12	315	3.9
C57BL/6J	2952	B6.129S4-Il2ra<tm1Dw>/J	12	239	5.4
C57BL/6J	3174	B6.129S4-Il2rg<tm1Wjl>/J	16	276	2.4
C57BL/6J	3991	B6.129S4-Itgam<tm1Myd>/J	6	122	13.9
C57BL/6J	3515	B6.129S4-Lyn<tm1Sor>/J	33	798	8.1
C57BL/6J	3755	B6.129S4-Meox2<tm1(cre)Sor>/J	6	163	3.1
C57BL/6J	2986	B6.129S4-Nos1<tm1Plh>/J	85	2204	4.5
C57BL/6J	2275	B6.129S4-Ntf3<tm1Jae>/J	43	862	3.4
C57BL/6J	3541	B6.129S4-Ntf3<tm2Jae>/J	16.5	622	3.9
C57BL/6J	4272	B6.129S4-Pdyn<tm1Ute>/J	30	624	3.4
C57BL/6J	4189	B6.129S4-Prkce<tm1Msg>/J	10	282	3.2
C57BL/6J	3322	B6.129S4-Soat1<tm1Far>/J	81	2248	14.0
C57BL/6J	4185	B6.129S4-Soat2<tm1Far>/J	6	149	1.3
C57BL/6J	2719	B6.129S4-Wt1<tm1Jae>/J	18	611	3.0
C57BL/6J	4038	B6.129S4-Zp3<tm1Dean>/J	15	361	5.5
C57BL/6J	4125	B6.129S6-Abcb11<tm1Wng>/J	6	135	2.2

C57BL/6J	2515	B6.129S6-Cftr<tm1Kth>/J	21	790	4.4
C57BL/6J	4267	B6.129S6-Dnmt3l<tm1Bes>/J	22	664	1.2
C57BL/6J	3321	B6.129S6-Gba<tm1Nsb>/J	101	2301	6.1
C57BL/6J	3827	B6.129S6-Naglu<tm1Efn>/J	19	717	11.1
C57BL/6J	2646	B6.129S6-Nf1<tm1Fcr>/J	26	828	4.3
C57BL/6J	4197	B6.129S6-Rac2<tm1Mddw>/J	30	598	3.3
C57BL/6J	3863	B6.129S6-Tapbp<tm1Luc>/J	0	305	12.5
C57BL/6J	2741	B6.129S7-Alpl<tm1Sor>/J	67	1311	2.9
C57BL/6J	4142	B6.129S7-Aplp2<tm1Dbo>/J	16	334	7.5
C57BL/6J	3783	B6.129S7-Bmp7<tm1Kry>/J	4	80	0.0
C57BL/6J	3336	B6.129S7-Cdkn1c<tm1Sje>/J	40	1565	4.0
C57BL/6J	4164	B6.129S7-Chrna3<tm1Bay>/J	28	749	6.3
C57BL/6J	3232	B6.129S7-Chrna7<tm1Bay>/J	14	423	6.3
C57BL/6J	2192	B6.129S7-Gt(ROSA)26Sor/J	21	750	2.7
C57BL/6J	3288	B6.129S7-Ifngr1<tm1Agt>/J	10	313	8.0
C57BL/6J	3329	B6.129S7-Itgb2<tm2Bay>/J	107	2190	5.9
C57BL/6J	3819	B6.129S7-Per2<tm1Brd>/J	29	783	8.1
C57BL/6J	2277	B6.129S7-Src<tm1Sor>/J	28	751	1.9
C57BL/6J	3726	B6.129S-Cd14<tm1Frm>/J	6	192	1.0
C57BL/6J	3180	B6.129S-Cdh3<tm1Hyn>/J	9	277	9.1
C57BL/6J	4276	B6.129S-Fign<tm1Frk>	18	585	7.7
C57BL/6J	2463	B6.129S-Itga4<tm1Hyn>/J	22	217	2.1
C57BL/6J	2818	B6.129-Tnfrsf1a<tm1Mak>/J	12	343	5.7
C57BL/6J	2994	B6.129X1-Bax<tm1Sjk>/J	6	150	1.3
C57BL/6J	3654	B6.129X1-Gad2<tm1Bae>/J	25	603	1.2
C57BL/6J	3126	B6.129X1-Grpr<tm1Jfb>/J	38	1008	18.5
C57BL/6J	3770	B6.129X1-Trpv1<tm1Jul>/J	12	320	6.5
C57BL/6J	4341	B6.129X-Cxcr4<tm1Qma>/J	12	396	4.5
C57BL/6J	4361	B6.129-Xrcc5<tm1Nus>/J	30	885	14.5
C57BL/6J	2651	B6.C3(Cg)-Rora<sg>/J	6	197	0.5
C57BL/6J	3479	B6.C3-Tg(Fos-luc)1Rnd/J	24.5	894	9.4
C57BL/6J	3904	B6.CBA-Tg(CETP)5203Tall/J	6	172	0.6
C57BL/6J	4275	B6.Cg-Fign<fi>/Frk	15	487	3.7
C57BL/6J	3574	B6.Cg-Tg(Alb-cre)21Mgn/J	9	394	6.3
C57BL/6J	3538	B6.Cg-Tg(APOC2)2Bres/J	5	61	3.3
C57BL/6J	4459	B6.Cg-Tg(BAC54)36Jt/J	6	119	0.8
C57BL/6J	2319	B6.Cg-Tg(BCL2)22Wehi/J	44	1013	1.6
C57BL/6J	2320	B6.Cg-Tg(BCL2)25Wehi/J	15	355	4.8
C57BL/6J	2321	B6.Cg-Tg(BCL2)36Wehi/J	24	783	1.0
C57BL/6J	3139	B6.Cg-Tg(DBHn-lacZ)8Rpk/J	15	362	1.9
C57BL/6J	3767	B6.Cg-Tg(Eno2tTA)5021Nes/J	17	627	2.9
C57BL/6J	3763	B6.Cg-Tg(Eno2tTA)5030Nes/J	31	901	1.6
C57BL/6J	3573	B6.Cg-Tg(Ins2-cre)25Mgn/J	15	412	3.2
C57BL/6J	3802	B6.Cg-Tg(Lck-cre)548Jxm/J	19.5	745	1.7
C57BL/6J	2210	B6.Cg-Tg(Mt1)174Bri/J	25.5	1040	3.5
C57BL/6J	3556	B6.Cg-Tg(Mx1-cre)1Cgn/J	6	192	2.1
C57BL/6J	3771	B6.Cg-Tg(Nes-cre)1Kln/J	18	478	1.0
C57BL/6J	3967	B6.Cg-Tg(Rbp3-cre)528Jxm/J	66	1636	4.8
C57BL/6J	2298	B6.Cg-Tg(SOD1)2Gur/J	18	475	1.8
C57BL/6J	2299	B6.Cg-Tg(SOD1*G93A)<dl>1Gur/J	29	969	7.3
C57BL/6J	4435	B6.Cg-Tg(SOD1*G93A)1Gur/J	12	393	0.7
C57BL/6J	3966	B6.Cg-Tg(Syn1-cre)671Jxm/J	47	1087	12.0
C57BL/6J	4128	B6.Cg-Tg(Tek-cre)12Flv/J	6	157	5.7
C57BL/6J	3762	B6.Cg-Tg(tetFosb)4468Nes/J	15	153	9.6
C57BL/6J	3710	B6.Cg-Tg(Thy1-CFP)23Jrs/J	4	153	5.2
C57BL/6J	3709	B6.Cg-Tg(Thy1-YFP)16Jrs/J	12	348	0.8
C57BL/6J	3782	B6.Cg-Tg(Thy1-YFPH)2Jrs/J	15	639	2.2
C57BL/6J	3563	B6.Cg-Tg(tTALap)5Bjd/J	29	841	6.3
C57BL/6J	4039	B6.Cg-Zp1<tm1Dean>/J	18	696	2.2
C57BL/6J	3380	B6.FVB-Tg(C3-1-TAg)cJeg/J	12	209	2.8
C57BL/6J	3951	B6.P2-P2rx3<tm1Ckn>/J	37	694	2.3
C57BL/6J	3138	B6;129-Hprt1<tm1Detl>/J	10	205	2.6
C57BL/6J	3901	B6;129-Irs4<tm1Lhd>/J	6	130	19.2
C57BL/6J	2461	B6;129P2-Cbs<tm1Unc>/J	10	181	3.9
C57BL/6J	2963	B6;129-Pcsk2<tm1Dfs>/J	10	575	3.6
C57BL/6J	4313	B6;129-Ppt1<tm1Hof>/J	6	127	6.3
C57BL/6J	4314	B6;129-Ppt2<tm1Hof>/J	6	247	8.1
C57BL/6J	3263	B6;129S2-Cdkn1a<tm1Tyj>/J	7	245	7.3
C57BL/6J	3379	B6;129S2-Scarb1<tm1Kri>/J	3	117	0.9

C57BL/6J	2914	B6;129S4-Hexb<tm1Rlp>/J	5	133	2.3
C57BL/6J	3751	B6;129S4-Igh-6<tm1Che>/J	15	452	9.1
C57BL/6J	2751	B6;129S7-Cyp7a1<tm1Rus>/J	4	16	0.0
C57BL/6J	3283	B6;129S7-Fshb<tm1Zuk>/J	10	383	9.4
C57BL/6J	3120	B6;129S7-L1cam<tm1Sor>/J	4	41	0.0
C57BL/6J	3696	B6;129S-Thbs3<Gt(VICTR20)14Lex>/J	12	210	11.4
C57BL/6J	3694	B6;129S-Vamp8<Gt(VICTR20)17Lex>/J	56.5	1146	5.5
C57BL/6J	3789	B6;129S-Vmn2r10<Gt(VICTR20)23Lex>/J	0	109	31.2
C57BL/6J	4728	B6;129X1-Prdx6<tm1Pgn>/Pgn	5	138	37.7
C57BL/6J	3466	B6;D2-Tg(Sycp1-cre)4Min/J	22	832	74.4
C57BL/6J	3734	B6;FVB-Tg(GZMB-cre)1Jcb/J	53	1078	14.4
C57BL/6J	2621	B6;SJL-Tg(tetop-lacZ)2Mam/J	6	166	5.4
C57BL/6J	3299	B6;SWJ-Tg(TIMP3-lacZ)7Jeb/J	32	939	5.8
C57BL/6J	2300	B6SJL-Tg(SOD1*G93A)<dl>1Gur/J	12	139	30.2
C57BL/6J	4176	BKS.B6-Tub<tub>/Jng	13	271	2.6
C57BL/6J	3175	C57BL/6-Il5<tm1Kopf>/J	51	1257	11.7
C57BL/6J	664	C57BL/6J	45	1932	1.8
C57BL/6J	4764	C57BL/6J-Cdh23<v-8J>/J	10	222	1.8
C57BL/6J	2923	C57BL/6J-Clock<m1Jt>/J	45	1047	0.7
C57BL/6J	4109	C57BL/6J-Glra1<nmf11>/J	9	311	1.6
C57BL/6J	4517	C57BL/6J-hlb156/J	6	228	2.6
C57BL/6J	4811	C57BL/6J-nmf110/J	24	303	1.7
C57BL/6J	4747	C57BL/6J-nmf118/J	6	139	0.7
C57BL/6J	4110	C57BL/6J-nmf12/J	6	222	1.4
C57BL/6J	4085	C57BL/6J-nmf4/J	8	342	5.0
C57BL/6J	4442	C57BL/6J-nmf62/J	21	767	1.6
C57BL/6J	4468	C57BL/6J-nmf63/J	5	184	4.3
C57BL/6J	4470	C57BL/6J-nmf65/J	12	258	2.4
C57BL/6J	4472	C57BL/6J-nmf67/J	4	114	0.0
C57BL/6J	4107	C57BL/6J-nmf9/J	9	245	4.5
C57BL/6J	4156	C57BL/6J-Pcdh15<av-5J>/J	33	785	1.1
C57BL/6J	4766	C57BL/6J-Pde6b<rd1-2J>/J	5	146	1.4
C57BL/6J	4102	C57BL/6J-Scn8a<4J>/J	23	446	9.7
C57BL/6J	4105	C57BL/6J-Scn8a<5J>/J	28	553	8.1
C57BL/6J	4587	C57BL/6J-Szt1/FrkJ	6	209	1.9
C57BL/6J	2226	C57BL/6J-Tg(Alb1HBV)44Bri/J	21.5	524	4.6
C57BL/6J	2230	C57BL/6J-Tg(LckIl4)1315Dbl/J	22	502	4.6
C57BL/6J	2233	C57BL/6J-Tg(SV)7Bri/J	8	316	5.7
C57BL/6J	2500	C57BL/6J-Tg(WapIgf1)39Dlr/J	22	593	2.4
C57BL/6J	2499	C57BL/6J-Tg(WapIGFBP3)67Dlr/J	20	290	5.0
C57BL/6J	2835	C57BL/6-Relb<Tg(H2-K1/GH1)106Bri>/J	12.5	808	7.7
C57BL/6J	3475	C57BL/6-Tg(HLA-A2.1)1Enge/J	11	344	4.3
C57BL/6J	2595	C57BL/6-Tg(IghelMD4)4Ccg/J	20	590	1.1
C57BL/6J	2598	C57BL/6-Tg(KLK4mHEL)6Ccg/J	24	803	16.3
C57BL/6J	3567	C57BL/6-Tg(LCK-NFKBIA)5Dwb/J	38	881	21.1
C57BL/6J	3185	C57BL/6-Tg(PRG1)18Wlad/J	11	239	10.7
C57BL/6J	3186	C57BL/6-Tg(PRG3)9Wlad/J	18	363	4.2
C57BL/6J	2628	C57BL/6-Tg(SOD1)10Cje/J	13	285	5.6
C57BL/6J	2629	C57BL/6-Tg(SOD1)3Cje/J	25	387	1.6
C57BL/6J	3831	C57BL/6-Tg(TcraTcrb)1100Mjb/J	6	280	6.8
C57BL/6J	3333	C57BL/6-Tg(TF-CAT)48Gsa/J	8	314	0.3
C57BL/6J	3135	C57BL/6-Tg(TRAMP)8247Ng/J	6	271	0.7
C57BL/6J	3394	C57BL/6-Tg(Zp3-cre)3Mrt/J	15	268	10.1
C57BL/6J	3651	C57BL/6-Tg(Zp3-cre)93Knw/J	0	225	4.9
C57BL/6J	3242	C57BL/6-Tnfrsf1a<tm1Imx>/J	6	102	14.7
C57BL/6J	3581	CBy.129S4-Dab1<tm1Cpr>/J	8	275	3.6
C57BL/6J	C57BL/6J-490	Private Strain	10	435	8.7
C57BL/6J	C57BL/6J-491	Private Strain	4	125	4.0
C57BL/6J	C57BL/6J-550	Private Strain	35	992	5.4
C57BL/6J	C57BL/6J-551	Private Strain	24	492	3.1
C57BL/6J	C57BL/6J-552	Private Strain	10	199	0.5
C57BL/6J	C57BL/6J-553	Private Strain	19	498	4.7
C57BL/6J	C57BL/6J-554	Private Strain	24	672	3.2
C57BL/6J	C57BL/6J-555	Private Strain	25	660	1.6
C57BL/6J	C57BL/6J-556	Private Strain	10	391	2.7
C57BL/6J	C57BL/6J-557	Private Strain	15	492	3.3
C57BL/6J	C57BL/6J-558	Private Strain	33	636	5.0
C57BL/6J	C57BL/6J-559	Private Strain	5	95	2.1
C57BL/6J	C57BL/6J-560	Private Strain	6	123	0.0

C57BL/6J	C57BL/6J-561	Private Strain	10	271	9.6
C57BL/6J	C57BL/6J-562	Private Strain	6	57	7.0
C57BL/6J	C57BL/6J-563	Private Strain	1139	34800	4.2
C57BL/6J	C57BL/6J-564	Private Strain	10	295	3.4
C57BL/6J	C57BL/6J-565	Private Strain	19	446	0.8
C57BL/6J	C57BL/6J-566	Private Strain	15	593	1.9
C57BL/6J	C57BL/6J-567	Private Strain	11	236	8.7
C57BL/6J	C57BL/6J-568	Private Strain	9	264	2.2
C57BL/6J	C57BL/6J-569	Private Strain	10	216	0.5
C57BL/6J	C57BL/6J-570	Private Strain	10	302	7.3
C57BL/6J	C57BL/6J-571	Private Strain	14	344	3.2
C57BL/6J	C57BL/6J-572	Private Strain	27	511	2.4
C57BL/6J	C57BL/6J-573	Private Strain	27	699	1.2
C57BL/6J	C57BL/6J-574	Private Strain	24	609	3.5
C57BL/6J	C57BL/6J-575	Private Strain	15	491	7.9
C57BL/6J	C57BL/6J-576	Private Strain	15	385	2.6
C57BL/6J	C57BL/6J-577	Private Strain	9	254	0.4
C57BL/6J	C57BL/6J-578	Private Strain	15	482	9.8
C57BL/6J	C57BL/6J-579	Private Strain	35	816	1.6
C57BL/6J	C57BL/6J-580	Private Strain	20	603	1.1
C57BL/6J	C57BL/6J-581	Private Strain	9	304	2.0
C57BL/6J	C57BL/6J-582	Private Strain	9	313	3.5
C57BL/6J	C57BL/6J-583	Private Strain	36	527	1.7
C57BL/6J	C57BL/6J-584	Private Strain	12	335	5.7
C57BL/6J	C57BL/6J-585	Private Strain	15	462	4.4
C57BL/6J	C57BL/6J-586	Private Strain	15	567	2.0
C57BL/6J	C57BL/6J-587	Private Strain	19	671	2.2
C57BL/6J	C57BL/6J-588	Private Strain	21.5	763	1.4
C57BL/6J	C57BL/6J-589	Private Strain	18	533	1.3
C57BL/6J	C57BL/6J-590	Private Strain	15	469	2.5
C57BL/6J	C57BL/6J-591	Private Strain	6	187	11.2
C57BL/6J	C57BL/6J-592	Private Strain	16.5	498	7.5
C57BL/6J	C57BL/6J-593	Private Strain	6	100	9.0
C57BL/6J	C57BL/6J-594	Private Strain	5	151	4.6
C57BL/6J	C57BL/6J-595	Private Strain	0	217	0.9
C57BL/6J	C57BL/6J-596	Private Strain	4	125	4.8
C57BL/6J	C57BL/6J-597	Private Strain	11	273	3.7
C57BL/6J	C57BL/6J-598	Private Strain	6	139	4.3
C57BL/6J	C57BL/6J-599	Private Strain	6	126	0.0
C57BL/6J	C57BL/6J-600	Private Strain	6	201	21.4
C57BL/6J	C57BL/6J-601	Private Strain	27	995	3.9
C57BL/6J	C57BL/6J-602	Private Strain	8	310	3.9
C57BL/6J	C57BL/6J-603	Private Strain	10	276	0.7
C57BL/6J	C57BL/6J-604	Private Strain	26	856	1.7
C57BL/6J	C57BL/6J-605	Private Strain	15	390	2.5
C57BL/6J	C57BL/6J-606	Private Strain	14	523	0.4
C57BL/6J	C57BL/6J-607	Private Strain	18	607	2.6
C57BL/6J	C57BL/6J-608	Private Strain	11	445	1.3
C57BL/6J	C57BL/6J-609	Private Strain	16	498	2.7
C57BL/6J	C57BL/6J-610	Private Strain	11	376	1.1
C57BL/6J	C57BL/6J-611	Private Strain	23	270	1.5
C57BL/6J	C57BL/6J-612	Private Strain	5	142	33.8
C57BL/6J	C57BL/6J-613	Private Strain	5	210	4.3
C57BL/6J	C57BL/6J-614	Private Strain	16	474	21.6
C57BL/6J	C57BL/6J-615	Private Strain	4	140	32.9
C57BL/6J	C57BL/6J-616	Private Strain	16	418	15.6
C57BL/6J	C57BL/6J-617	Private Strain	16	529	33.8
C57BL/6J	C57BL/6J-618	Private Strain	4	87	8.0
C57BL/6J	C57BL/6J-619	Private Strain	8	375	2.3
C57BL/6J	C57BL/6J-620	Private Strain	4	158	11.4
C57BL/6J	C57BL/6J-621	Private Strain	4	120	7.5
C57BL/6J	C57BL/6J-622	Private Strain	4	151	7.9
C57BL/6J	C57BL/6J-623	Private Strain	4	159	0.0
C57BL/6J	C57BL/6J-624	Private Strain	8	224	2.5
C57BL/6J	C57BL/6J-625	Private Strain	8	282	4.8
C57BL/6J	C57BL/6J-626	Private Strain	12	473	12.9
C57BL/6J	C57BL/6J-627	Private Strain	10	309	9.4
C57BL/6J	C57BL/6J-628	Private Strain	10	336	25.3
C57BL/6J	C57BL/6J-629	Private Strain	5	113	8.0

C57BL/6J	2267	STOCK Bdnf<tm1Jae>/J	20	639	2.5
C57BL/6J	4711	STOCK Ednrb<s-52Pub>	6	121	34.7
C57BL/6J	3820	STOCK Fbn2<fp-3J>	8	161	8.7
C57BL/6J	3304	STOCK Rb(16.17)7Bnr-Myo15<sh2-2J>/J	24	539	2.1
C57BL/6J	3318	STOCK Shh<tm1Amc>/J	9	249	2.4
C57BL/6J	3919	STOCK Tg(ACTB-Bgeo/ALPP)1Lbe/J	26	641	14.3
C57BL/6J	3275	STOCK Tg(tetL)1Bjd/J	40	1273	1.8
C57BL/6J	3274	STOCK Tg(tetNZL)2Bjd/J	55	1825	12.7
C57BL/6J	3549	STOCK Tg(TTR-GLVP)1074Tsa/J	24	763	5.1
C57BL/6J	4184	STOCK Tg(Wap-HRAS)69Lln Chr Y<SJL>-Edaradd<cr-3J>/J	15	448	3.3
DBA/2J	4798	D.B10	5	123	72.4
DBA/2J	DBA/2J-10	Private Strain	18	523	57.9
DBA/2J	DBA/2J-11	Private Strain	5	109	20.2
DBA/2J	DBA/2J-12	Private Strain	15	217	76.5
DBA/2J	DBA/2J-13	Private Strain	12	345	79.7
DBA/2J	DBA/2J-14	Private Strain	6	115	93.9
DBA/2J	DBA/2J-15	Private Strain	10	191	80.6
DBA/2J	DBA/2J-16	Private Strain	0	516	65.7
DBA/2J	DBA/2J-9	Private Strain	25	379	86.0
FVB/NJ	2539	FVB.129P2-Abcb4<tm1Bor>/J	79	1093	34.2
FVB/NJ	2935	FVB.129S2(B6)-Ccnd1<tm1Wbg>/J	52	416	15.4
FVB/NJ	2899	FVB.129S2(B6)-Trp53<tm1Tyj>/J	46	503	36.3
FVB/NJ	3502	FVB.129S6-Stat5a<tm1Mam>/J	20	299	29.5
FVB/NJ	3516	FVB.Cg-Tg(ACTB-EGFP)B5Nagy/J	5	92	9.8
FVB/NJ	2953	FVB.Cg-Tg(MMTVTGFA)254Rjc/J	31	458	56.2
FVB/NJ	3170	FVB.Cg-Tg(Myh6-tTA)6Smbf/J	84	1162	2.1
FVB/NJ	3739	FVB.Cg-Tg(Pbsn-Cdc37)1Stp/J	22	279	28.2
FVB/NJ	2934	FVB.Cg-Tg(Wnt1)1Hev/J	26	446	8.6
FVB/NJ	3487	FVB/NJ-Tg(XGFAP-lacZ)3Mes/J	10	188	4.8
FVB/NJ	3640	FVB/NJ-Tg(YAC72)2511Hay/J	10	164	34.7
FVB/NJ	3314	FVB/N-Tg(EIIa-cre)C5379Lmgd/J	6	75	0.0
FVB/NJ	3257	FVB/N-Tg(GFAPGFP)14Mes/J	9	181	18.8
FVB/NJ	2374	FVB/N-Tg(MMTV-PyVT)634Mul/J	73	759	10.7
FVB/NJ	2421	FVB/N-Tg(MtTGFA)100Lmb/J	10	75	10.0
FVB/NJ	3315	FVB/N-Tg(tetORo1-lacZ)3Conk/J	24	380	18.7
FVB/NJ	2856	FVB/N-Tg(TIE2-lacZ)182Sato/J	14	199	46.1
FVB/NJ	2659	FVB/N-Tg(Trp53R172H)8512Jmr/J	58	646	75.5
FVB/NJ	2677	FVB/N-Tg(WapMyc)212Bri/J	10	110	6.4
FVB/NJ	2755	FVB/N-Tg(WapNotch4)10Rnc/J	6	279	43.3
FVB/NJ	4066	FVB;129S-Men1<tm1.1Ctre>/J	15	231	22.4
FVB/NJ	3078	FVB-Tg(WapIgf1)39Dlr/J	22	125	0.0
FVB/NJ	3528	STOCK Tg(GFAP-TVA)5Hev/J	17	189	82.0
FVB/NJ	3529	STOCK Tg(NES-TVA)12Hev/J	12	175	12.7
FVB/NJ	3658	STOCK Tg(TIE2GFP)287Sato/J	20	235	14.0
NOD/ShiLtJ	2309	NOD.129P2(B6)-B2m<tm1Unc>/J	60	1248	45.8
NOD/ShiLtJ	3729	NOD.129S7(B6)-Rag1<tm1Mom>/J	6	77	6.5
NOD/ShiLtJ	3653	NOD.129X1-Gad2<tm1Bae>/J	61	1234	16.3
NOD/ShiLtJ	2570	NOD.Cg-Prkdc<scid> B2m<tm1Unc>/J	15	262	59.9
NOD/ShiLtJ	1289	NOD/ShiLt	1.5	49	65.3
NOD/ShiLtJ	NOD/ShiLtJ-7	Private Strain	9	106	12.3
